# Supplementary material for: Space ionizing radiation triggers the formation of peptides and organophosphates on olivine surfaces
Source: Nat Commun. 2026 Feb 23;17:3210. doi: 10.1038/s41467-026-69575-x (PMC13057002; doi:10.1038/s41467-026-69575-x)
Supplement: Supplementary file 1 — Supplementary Information [file 41467_2026_69575_MOESM1_ESM.pdf]

Supplementary Information

Space ionizing radiation triggers the formation of peptides and organophosphates on olivine surfaces

Ruiwen Ding [a], Shiwen Qiu [a], Xiaofan Guo [a], Min Zhang [a], Zhiyun Zou [a], Yan Liu [a*], Jianxi Ying [b], Meng Zhang [c], Bingquan Zhang [d], Yufen Zhao [a,b,e]

[a] *Department of Chemical Biology, Fujian Key Laboratory of Chemical Biology (Xiamen University), College of Chemistry and Chemical Engineering, Xiamen University, Xiamen, Fujian, 361005, RP China.*

[b] *Institute of Drug Discovery Technology, Ningbo University, Ningbo, Zhejiang, 315221, RP China.*

[c] *College of Environmental Sciences and Engineering, Dalian Maritime University, Dalian, Liaoning, 116000, RP China.*

[d] *National Space Science Center of the Chinese Academy of Sciences, Beijing 100190, PR China*

[e] *Department of Chemistry, Tsinghua University, Beijing, 100084, PR China.*

*Corresponding authors: stacyliu@xmu.edu.cn (Yan Liu)

Supplementary Information

Catalogue

1. The sample unit for Space Radiobiological Exposure Facility on the China Space Station (CSS) 5

2. The standard protocols for the radiation experiments 6

2.1 The minerals processing process 6

2.2 The mixed amino acids reaction system 7

2.3 The mixed amino acids reaction system in the presence of nucleosides and sodium trimetaphosphate 7

2.4 The single amino acid reaction system in the presence of nucleosides and sodium trimetaphosphate 8

2.5 The setting of the experimental groups 8

2.6 Ground radiation experiment of the control group 8

2.7 Pretreatment methods for sample analysis following the completion of reactions 9

2.8 General nucleotide amidates (*N*-Phe-AMP and *N*-Ile-AMP) preparations in an alkaline aqueous solution. 9

3. Analysis methods 9

3.1 HPLC analysis 9

3.2 MS analysis 11

3.3 X-Ray Diffraction (XRD) analysis 11

3.4 X-Ray Photoelectron Spectroscopy (XPS) analysis 11

3.5 NMR analysis 12

3.6 pH Measurement of Mineral-Reaction System Mixtures 12

4. Environmental parameters of the experiment systems onboard the CSS 12

5. Powder XRD patterns analysis of the forsterite used in the experiment systems 14

6. Powder XPS patterns of forsterite used in the experiments 15

7. MS analysis results of the related dipeptide products in the mixed amino acid reaction systems of space radiation condition and ground control 16

8. MS analysis of NMP standard substance 33

9. MS analysis of NMP in the mixed amino acid, nucleosides and P_3m reaction system under the space radiation condition in C3 Unit of TZ 6 36

10. MS analysis of the dipeptide and NMP product formed in the reaction system containing the mixed amino acid, nucleosides and P_3m after space radiation exposure in B3 Unit of TZ 6 44

11. MS analysis of the products formed in the reaction system containing Phe, nucleosides (A and U) and P_3m under the ground radiation conditions 56

12. MS analysis of the products formed in the reaction system containing Phe, nucleosides (C and G), and P_3m with forsterite on the ground radiation condition 64

13. MS analysis in the mixed Phe and P_3m reaction system of the ground condition 70

14. MS analysis in the Phe reaction system without P_3m under the ground condition 72

15. MS analysis of the reaction system containing the mixed amino acid, nucleosides and P_3m after space ionizing radiation exposure in A2 Unit of TZ 7 .. 75

16. Characterization of the compound *N*-Phe-AMP obtained through two different abiotic synthesis strategies by UPLC-HRMS 79

16.1 Formation of *N*-Phe-AMP from a reaction mixture of Phe, adenosine and P_3m with forsterite under radiation 79

16.2 Formation of *N*-Phe-AMP from a reaction mixture of Phe, adenosine and P_3m in an alkaline aqueous solution (pH 11) 81

17. Characterization of the compound *N*-Ile-AMP obtained through two different abiotic synthesis strategies by UPLC-HRMS 83

17.1 Formation of *N*-Ile-AMP from a reaction mixture of Ile, adenosine and P_3m with forsterite under radiation 83

17.2 Formation of *N*-Ile-AMP from a reaction mixture of Ile, adenosine and P_3m in an alkaline aqueous solution (pH 11) 85

18. Radical quenching experiment and the role of magnesium ion in the reaction systems 88

19. ^{31}P NMR tracking the ring-opening reaction of P_3m under X-ray radiation 92

20. MS analysis in the mixed reaction system with Phe and hydroxyapatite under the ground radiation condition 92

20.1 MS analysis in the Phe and hydroxyapatite reaction system under the ground condition 92

20.2 MS analysis of the products formed in the reaction system containing Phe, nucleosides (A and U), and hydroxyapatite under the ground radiation condition 95

20.3 MS analysis of the products formed in the reaction system containing Phe, nucleosides (C and G), and hydroxyapatite under the ground radiation conditions 97

20.4 Quantitative analysis of products formed in the Phe and hydroxyapatite system under the ground radiation conditions 99

21. Calibration curves of dipeptide or nucleotide using AB SCIEX QTrap™ 5500 LC–MS instrument 100

22. Radiolytic yields (G value) 106

1. The sample unit for Space Radiobiological Exposure Facility on the China Space Station (CSS)

The sample unit is made of polyimide, and its individual container and modular design are shown in Figure S1. The experimental samples are loaded into sealed vials with double-threaded caps and securely placed on threaded sample fixing disks. Five sample disks loading with 80 independent samples form an independent sample unit box. The sample unit was placed in the Space Radiobiological Exposure Facility (SREF). The radiation dose was mainly monitored by the radiation measurement subsystem. The total radiation dose received by each unit was determined as the average of the measured values from predeployed lithium 6 or 7 passive detection chips, including the doses received while standing inside the cabin. A comprehensive characterization of SREF on the China Space Station has been documented in the published literature (Binquan Zhang*, et al. Astrobiology*, 2025, **25**, 32-41.). The radiation absorbed dose obtained in this experiment was measured by a radiation thermoluminescence detector and mainly consisted of radiation with LET values less than 10 keV/μm, including protons, neutrons and some heavy ion radiation.

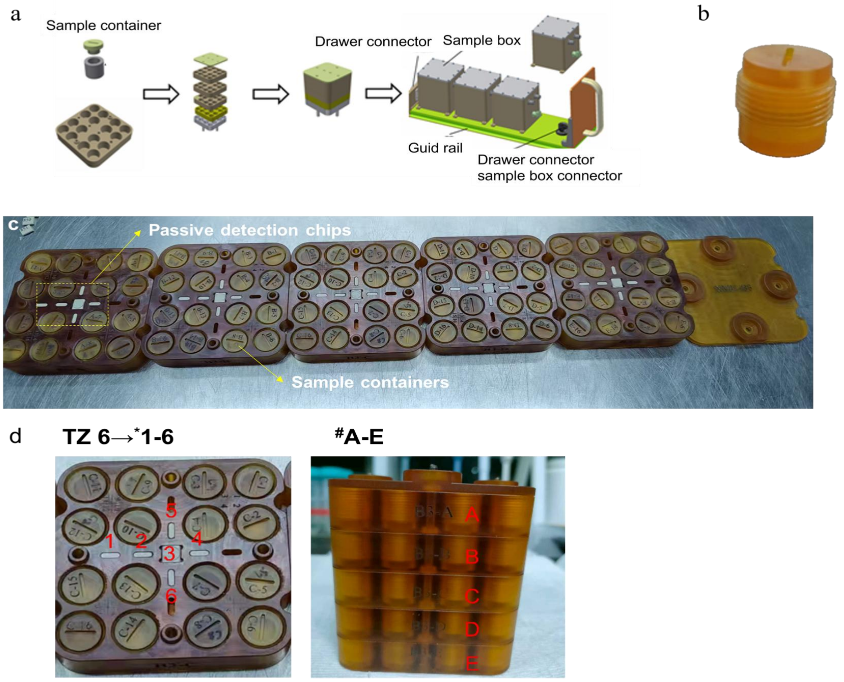

Supplementary Figure 1. The schematic diagram of the Space Radiobiological Exposure Facility.

a. The overall scheme of the experiment device; b. The single sample container (Polyimide material); c. The layout of passive detection chips in the sample unit. d. The layout of each layer of the polyimide sample box (right) and the passive detection chips (left). Their corresponding irradiation values are summarized in Supplementary Table 1.

Supplementary Table 1. The irradiation measurement detected by passive detection chips

Radiation Dose (mGy) / Layer of Sample Box / Detection Chip		1	2	3	4	5	6
TZ-6	B3-A#	78.9	73.2	73.4	71.2	73.2	71.5
	B3-B#	82.8	78.3	74.7	74.6	76.3	77.1
	B3-C#	74.1	81.4	78.9	75.8	84.8	81.3
	B3-D#	95.6	86.7	83.7	85.9	88.8	81.2
	B3-E#	103.9	105.9	98.8	105.9	98.8	98.0
	C3-A#	33.3	31.6	36.0	35.6	33.7	31.4
	C3-B#	31.7	27.7	32.6	37.6	32.9	32.5
	C3-C#	35.4	28.5	35.4	37.5	35.3	34.0
	C3-D#	40.9	33.1	41.6	39.4	40.1	41.1
	C3-E#	44.5	37.8	39.5	45.4	48.7	47.9

Notes: Numbers from 1 to 6 are the serial numbers of the passive detection chips in each layer (Supplementary Figure 1d (left)). #: The serial numbers of each layer of the sample box.

2. The standard protocols for the radiation experiments

2.1 The minerals processing process

The minerals were manually ground into a fine powder with a mortar and ground to pass a 320-mesh sieve. Then, forsterite was cleaned to remove organic contamination according to the following methods: 1) deionized water, sonication for 0.5 h; 2) methanol, sonication for 0.5 h; 3) methylene chloride, sonication for 0.5 h; 4) methanol, sonication for 0.5 h; 5) deionized water, sonication for 0.5 h (*Proc. Natl. Acad. Sci.,* 2001, **98**, 5487-5490; *Front. Astron. Space Sci.,* 2020, **7**, 539289). We performed alternating extraction steps with centrifugation, separation of supernatant, and freeze-drying. Subsequently, the forsterite and Martian soil simulant were

oven-dried at 100°C for 2 hours.

2.2 The mixed amino acids reaction system

The Phe, Ala, and Arg solutions (30 mmol/L each) were individually sampled in equi-volume proportions and thoroughly mixed in a 1.5 mL EP tube to ensure thorough mixing. The above amino acid mixing solution was divided into two parts and put into the two sample containers of the experiment unit made from polyimide, ensuring a total solution volume of 200 μL for each one. One sample container was prefilled with forsterite 50 mg as the experimental group, another one was taken as the blank control group without minerals. The above samples were freeze-dried in preparation for the subsequent radiation experiment. Three parallel experimental groups are set up for each group of samples.

2.3 The mixed amino acids reaction system in the presence of nucleosides and sodium trimetaphosphate

The amino acid solution (Phe, Ala or Arg) with a concentration of 30 mmol/L, the sodium trimetaphosphate (P_3m) solution with a concentration of 30 mmol/L, and the nucleoside (A/U or C/G) solution with a concentration of 15 mmol/L were individually sampled in equi-volume proportions and thoroughly mixed in a 1.5 mL EP tube.

The above mixing solution was divided into two parts and put into the two sample containers of the experiment unit made from polyimide, ensuring a total solution volume of 200 μL for each one. One sample container was prefilled with 50 mg of forsterite as the experimental group, and another was taken as the blank control group without minerals. The above samples were freeze-dried in preparation for the subsequent radiation experiment.

2.4 The single amino acid reaction system in the presence of nucleosides and sodium trimetaphosphate

The Phe solution with a concentration of 30 mmol/L, the P_3m solution with a concentration of 30 mmol/L, and the nucleoside (A/U or C/G) solution with a concentration of 15 mmol/L were individually sampled in equi-volume proportions and thoroughly mixed in a 1.5 mL EP tube. Then, the final concentration of Phe in the above mixing solution is 10 mmol/L.

The above mixing solution was divided into two parts and put into the two sample containers, ensuring a total solution volume of 200 μL for each one. One sample container was prefilled with 50 mg of forsterite as the experimental group, and another was taken as the blank control group without minerals. The above samples were freeze-dried in preparation for the subsequent radiation experiment.

2.5 The setting of the experimental groups

Multiple experimental groups were established for each reaction system mentioned above, with one group designated as the experimental group aboard the China Space Station. Correspondingly, the remaining groups served as control groups on the ground. All the experiments described herein were replicated thrice.

2.6 Ground radiation experiment of the control group

The control group samples were irradiated using the RS2000 X-ray biological irradiator (Rad Source Technologies, Inc., America). The simulated space radiation dose (63.63 mGy) was obtained using exposure parameters of 160 kV, 5 mA, and 17 s and a custom-made copper filter measuring 0.3 mm thick. The radiation dose of 1Gy was exposed to factors of 160 kV, 25 mA, and 52 s, and the custom-made filter was made of 0.3 mm copper. The radiation dose of 20 Gy was exposure factors of 160 kV, 25 mA, and 240 s without a 0.3 mm copper filter. The detailed information is illustrated in the Supporting Information section.

2.7 Pretreatment methods for sample analysis following the completion of reactions

Samples containing minerals were initially dissolved in a 20 mmol/L NH_4HCO_3 solution, followed by centrifugation to separate the supernatant after vortexing. The resulting precipitate was then treated with a mixture of 50% ethanol solution, followed by another round of centrifugation to obtain the supernatant. All collected supernatants were combined and subsequently freeze-dried using a freeze-dryer.

Eventually, all the experimental and control group samples were redissolved with 5% acetonitrile for the following analysis by spectroscopy analysis.

2.8 General nucleotide amidates (*N*-Phe-AMP and *N*-Ile-AMP) preparations in an alkaline aqueous solution.

0.1 mmol sodium trimetaphosphate (P_3m) and 0.1 mmol amino acid (Phe or Ile) were dissolved and well-mixed with 0.1 mmol adenosine in 1 mL water, respectively. The pH of the reaction mixture solution was adjusted to 11.7 using 10 M NaOH. Then, the reaction mixture solution was placed at 45°C for 5 h incubation.

3. Analysis methods

3.1 HPLC analysis

For the *L*-Phe reaction system, the HPLC separation of the related products was conducted using an ACE Excel 3 AQ column (particle size: 3.0 μm, dimensions: 3×100 mm) at room temperature. Gradient elution was programmed using two mobile phases; solvent A was 0.1% formic acid water, and solvent B was 0.1% formic acid acetonitrile. The gradient elution conditions were 0-4 min, 2-30% B; 4-8 min, 30-40% B; 8-9 min, 40-100% B; 9-12 min, 100-2% B; 12-17 min, 2% B. The flow rate was 0.4 $mL \cdot min^{-1}$, and the DAD detector was 256 nm.

Arg, Ala and Phe mixed reaction systems analyzed the resultant products with a

Poroshell 120 HILIC column (particle size: 1.9 μm, dimensions: 2.1×150 mm) at room temperature. The gradient elution conditions were 0-20 min, 80% B. The flow rate was 0.2 mL·min^{-1}.

For the *N*-Phe-AMP and *N*-Ile-AMP, the HPLC was fitted with an Agilent TC-C18, 5 μm, 4.6 mm × 150 mm column. The solvent A was 5 mM ammonium acetate, and solvent B was acetonitrile. The HPLC flow rate was 0.8 mL· min^{-1} with TC-C18, and the column temperature was maintained at room temperature.

For NMP, HPLC analysis was performed on the Thermo Ultimate 3000 HPLC system and fitted with an Agilent TC-C18, 5 μm, 4.6 mm × 150 mm column or Phenomenex Luna C18 column (5 μm, 4.6 mm × 250 mm) with the DAD detector at 210 nm. Solvent A was 0.1% formic acid water, and solvent B was 0.1% formic acid acetonitrile. The HPLC flow rate was 0.8 mL·min^{-1}, and the column temperature was maintained at room temperature. The gradient elution conditions: 0-5 min, 1% B; 5-20 min, 1-80% B; 20-22 min, 80% B; 22-24 min, 80-1% B; 24-30 min, 1% B. The structure of NMP was determined on a Thermo Ultimate 3000 HPLC system with an ACE Excel 3 AQ, 3 μm, 2.1 mm × 150 mm column. Solvent A was 0.1% formic acid water, and solvent B was 0.1% formic acid acetonitrile. The HPLC flow rate was 0.2 mL·min^{-1}, and the column temperature was maintained at room temperature. The gradient elution conditions: 0-2 min, 0% B; 2-12 min, 0-100% B; 12-13 min, 100-0% B; 13-18 min, 0% B.

For *L*-Phe reaction system quantitative analysis, HPLC analysis was performed on the ACQUITY UPLC I-Class PLUS system and fitted with an ACE Excel 3 AQ, 3 μm, 2.1 mm × 150 mm column. Solvent A was 0.1% formic acid water, and solvent B was 0.1% formic acid acetonitrile. The HPLC flow rate was 0.2 mL·min^{-1}, and the column temperature was maintained at room temperature. The gradient elution conditions: 0-2 min, 0% B; 2-12 min, 0-100% B; 12-13 min, 100-0% B; 13-20 min, 0% B.

3.2 MS analysis

For dipeptide, *N*-aa-NMP, MS and MS^2 analysis were performed on the Thermo Q Exactive parameters were as follows: positive mode; scan range, *m/z* 50-1000; dry gas flow rate, 4 $L·min^{-1}$; Spray voltage, 3.5 kV; and capillary temperature, 180°C.

For NMP, MS and MS^2 analysis were performed on the Thermo Scientific Q-Exactive™ Plus system in positive mode. The MS instrument parameters were: spray voltage of 3800 V, sheath gas flow rate of 3 $L·min^{-1}$, and capillary temperature of 320 °C. The mass spectra were acquired from *m/z* 60 to 900 in the scan range.

For quantitative analysis were performed on the QTrap™ 5500 (MRM mode, AB SCIEX) parameters were as follows: positive mode; scan range, *m/z* 50-1000; nitrogen was used for the curtain gas (set to 35), gas1 (set to 55) and gas 2 (set to 60), ion spray voltage (set to 5500V).

3.3 X-Ray Diffraction (XRD) analysis

XRD patterns were recorded on a Rigaku-Ultima IV XRD (Japan) diffractometer with Cu Kα radiation using a generator voltage of 40 kV and a generator current of 40 mA. The specimens were investigated from 30° and 80° (2θ) with a scan rate of 3°(2θ) /min.

3.4 X-Ray Photoelectron Spectroscopy (XPS) analysis

The forsterite samples were subjected to X-ray photoelectron spectroscopy analysis using a Shimadzu KRATOS AXIS SUPRA+ (England) diffractometer in an ultra-high vacuum chamber equipped with a hemispherical electron analyzer. An Al K_α X-ray source (1486.6 eV) with an aperture of 300 μm × 700 μm was employed. The base pressure in the chamber was 5×10^{-8} mbar, and the experiments were performed at room temperature. The XPS peak decomposition of different components was shaped, following background subtraction, as a convolution of Lorentzian and Gaussian curves, subsequently, the curves were analyzed using the

XPS Fitting software.

3.5 NMR analysis

All the ^{31}P NMR studies were carried out on a Bruker AVANCE 500 MHz spectrometer at room temperature. The samples were reconstituted in pure water and transferred to NMR tubes for ^{31}P NMR analysis.

3.6 pH Measurement of Mineral-Reaction System Mixtures

The pH values for all solutions were determined with a METTLER TOLED pH meter. After thorough mixing of the sample with the minerals, the mixture was centrifuged. The supernatant was then collected, and its pH was measured.

4. Environmental parameters of the experiment systems onboard the CSS

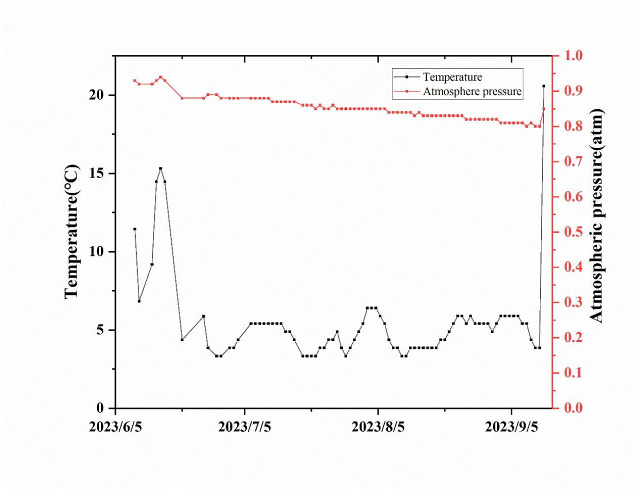

Supplementary Figure 2. The on-orbit temperature and atmosphere pressure of the C3 sample box (TZ 6 Mission, polyimide material, extravehicular exposure for 97 days).

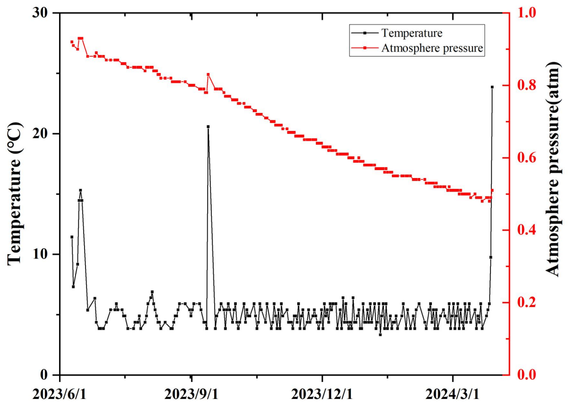

Supplementary Figure 3. The on-orbit temperature and atmosphere pressure of the B3 sample box (TZ 6 Mission, polyimide material, extravehicular exposure for 294 days).

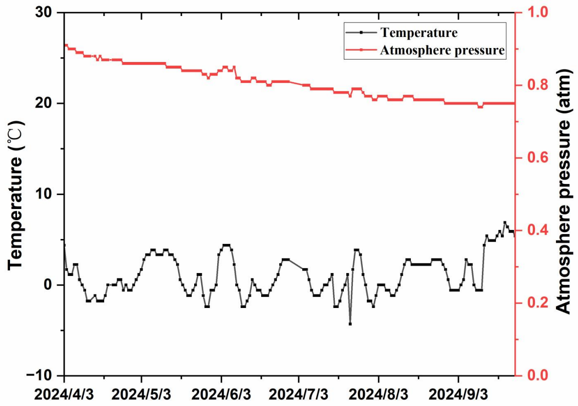

Supplementary Figure 4. The on-orbit temperature and atmosphere pressure of the A2 sample box (TZ 7 Mission, polyimide material, argon atmosphere, extravehicular exposure for 174 days).

5. Powder XRD patterns analysis of the forsterite used in the experiment systems

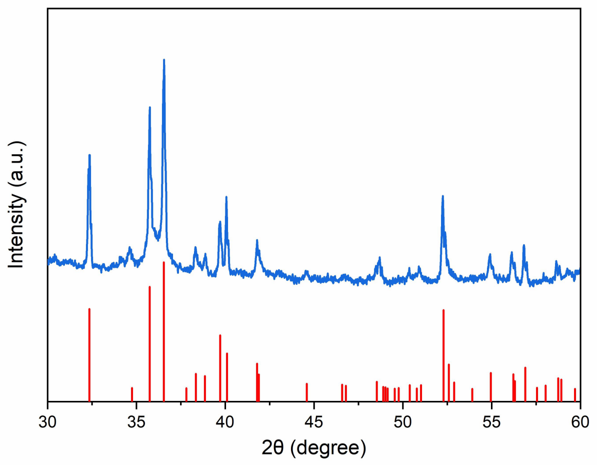

Supplementary Figure 5. The XRD pattern analysis of the forsterite used in the experiment systems (Blue: The sample of forsterite for experiments; Red: The standard forsterite). The XRD patterns comparative analysis results of raw material forsterite and the standard card indicate that the purchased forsterite is consistent with standard forsterite.

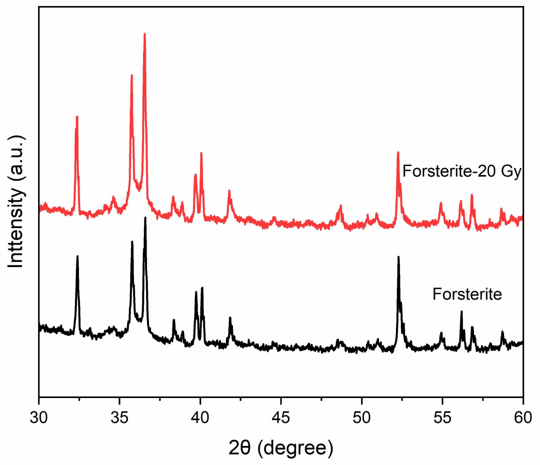

Supplementary Figure 6. The XRD pattern of the forsterite after radiation (X-ray 20 Gy, 240s).

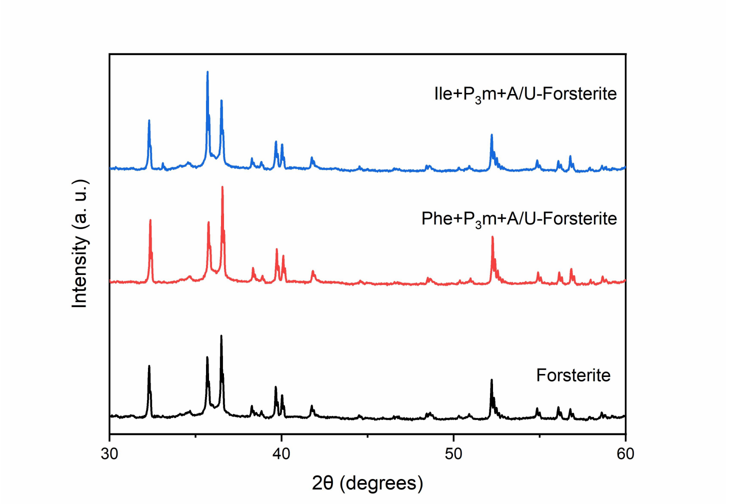

Supplementary Figure 7. The experiment system's XRD pattern after radiation contains amino acid (Ile/Phe), P3m, A/U and forsterite.

6. Powder XPS patterns of forsterite used in the experiments

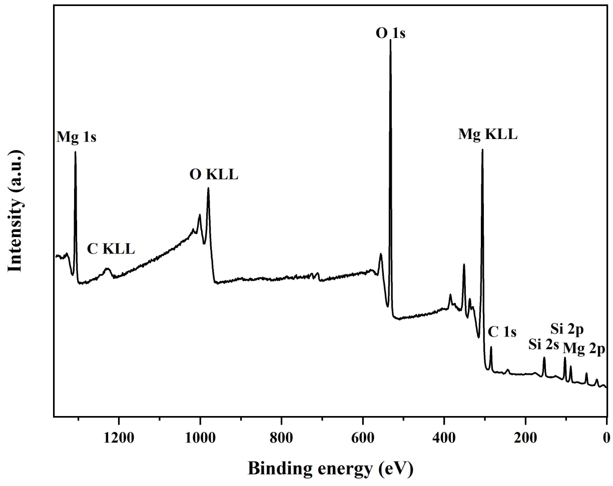

Supplementary Figure 8. The XPS broad scan spectrum of the raw material forsterite used in the experiments.

Supplementary Table 2. Chemical composition (atom %) of the forsterite based on the high-resolution XPS analysis.

Element	Pos.	FWHM	Atom%
O 1s	527.6	3.55	66.73
Si 2p	98.8	3.48	17.44
Mg 2s	84.4	3.40	15.82

7. MS analysis results of the related dipeptide products in the mixed amino acid reaction systems of space radiation condition and ground control

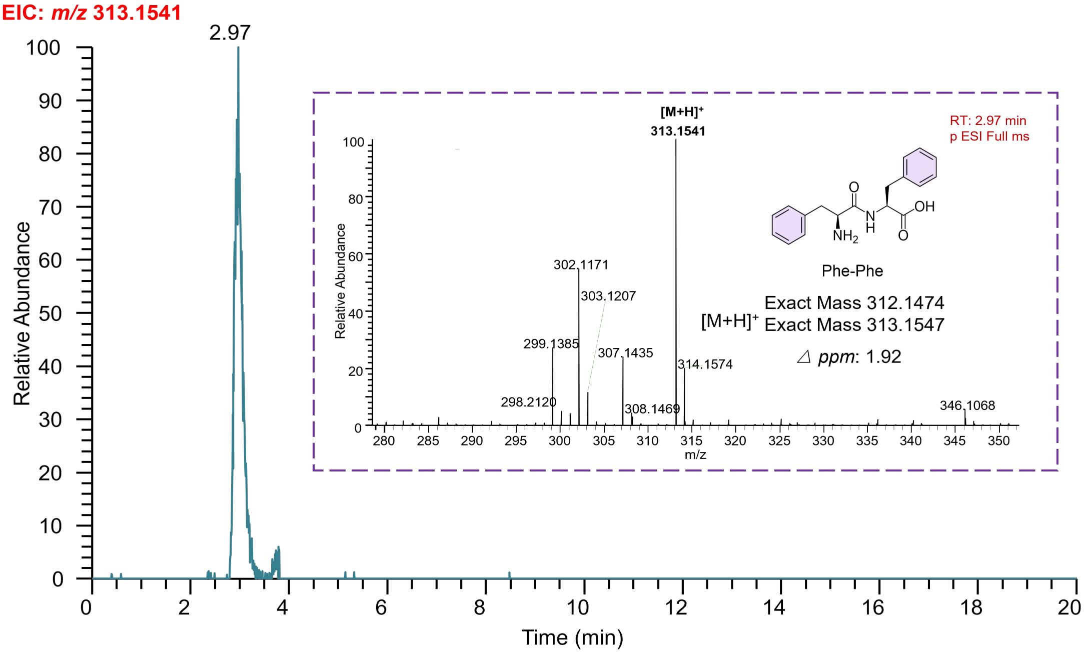

Supplementary Figure 9. The extracted ion chromatogram (EIC) and MS spectrum of Phe-Phe (*m/z* 313) dipeptide formed in the mixed amino acid (Ala, Phe and Arg) reaction systems with forsterite set up into Unit C3 with a total dose of 37.75 mGy onboard the CSS.

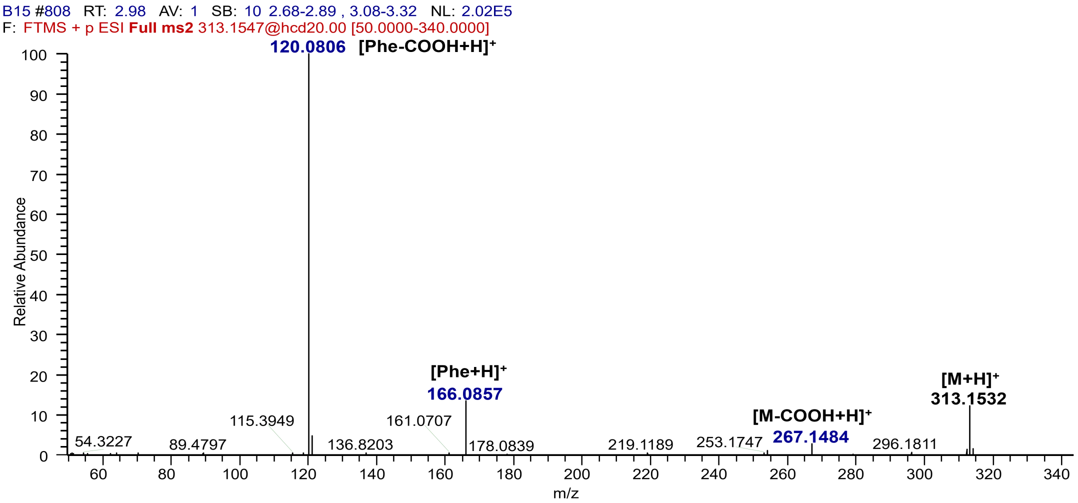

Supplementary Figure 10. MS^2 spectrum of the Phe-Phe dipeptide product. The

dipeptide sequence is assigned by employing a comparative analysis of b/y ion pairs and characteristic fragments.

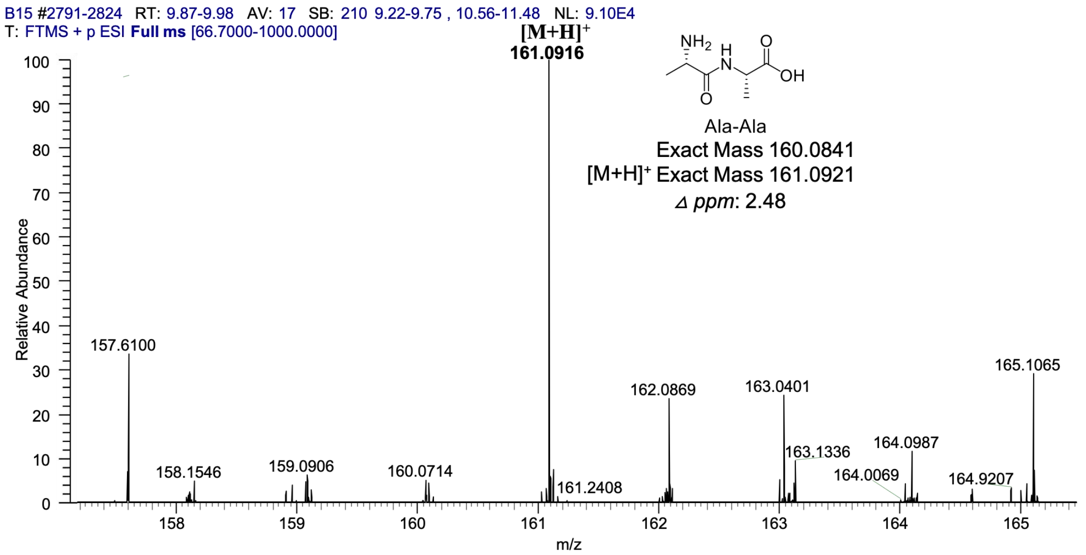

Supplementary Figure 11. The MS spectrum of Ala-Ala (*m/z* 161) dipeptide formed in the mixed amino acid (Ala, Phe and Arg) reaction systems with forsterite set up into Unit C3 with a total dose of 37.75 mGy onboard the CSS.

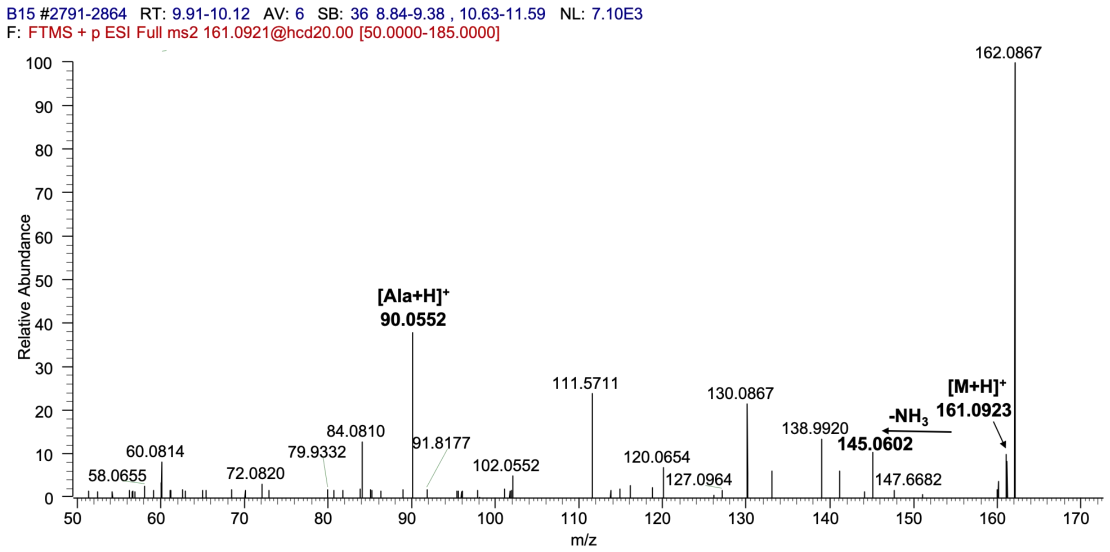

Supplementary Figure 12. MS^2 spectrum of the Ala-Ala dipeptide product. The dipeptide sequence is assigned by employing a comparative analysis of b/y ion pairs and characteristic fragments.

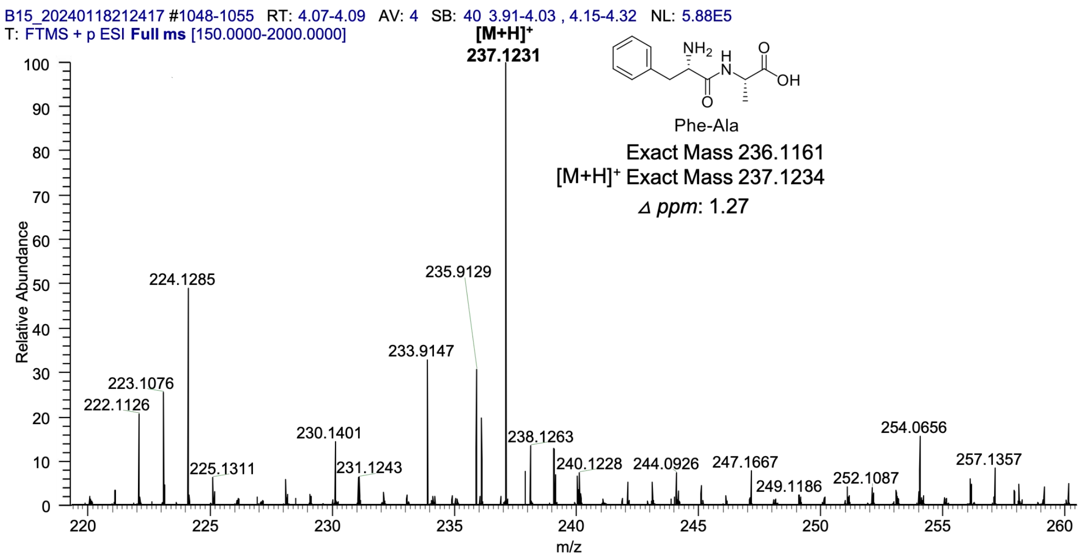

Supplementary Figure 13. The MS spectrum of Phe-Ala (*m/z* 237) dipeptide formed in the mixed amino acid (Ala, Phe and Arg) reaction systems with forsterite set up into Unit C3 with a total dose of 37.75 mGy onboard the CSS.

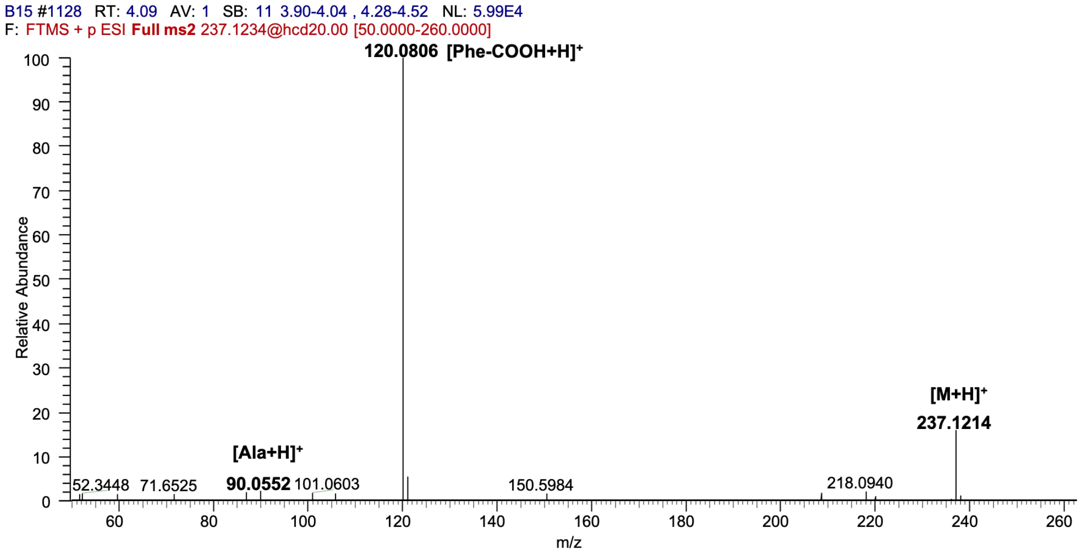

Supplementary Figure 14. The MS^2 spectrum of Phe-Ala dipeptide product. The dipeptide sequence is assigned by employing a comparative analysis of b/y ion pairs and characteristic fragments.

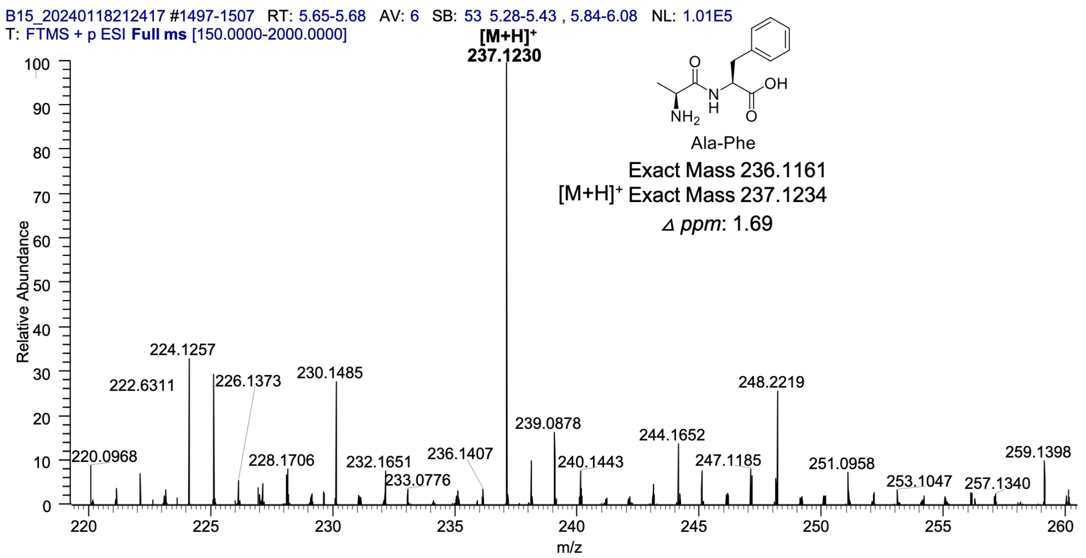

Supplementary Figure 15. The MS spectrum of Ala-Phe (*m/z* 237) dipeptide formed in the mixed amino acid (Ala, Phe and Arg) reaction systems with forsterite set up into Unit C3 with a total dose of 37.75 mGy onboard the CSS.

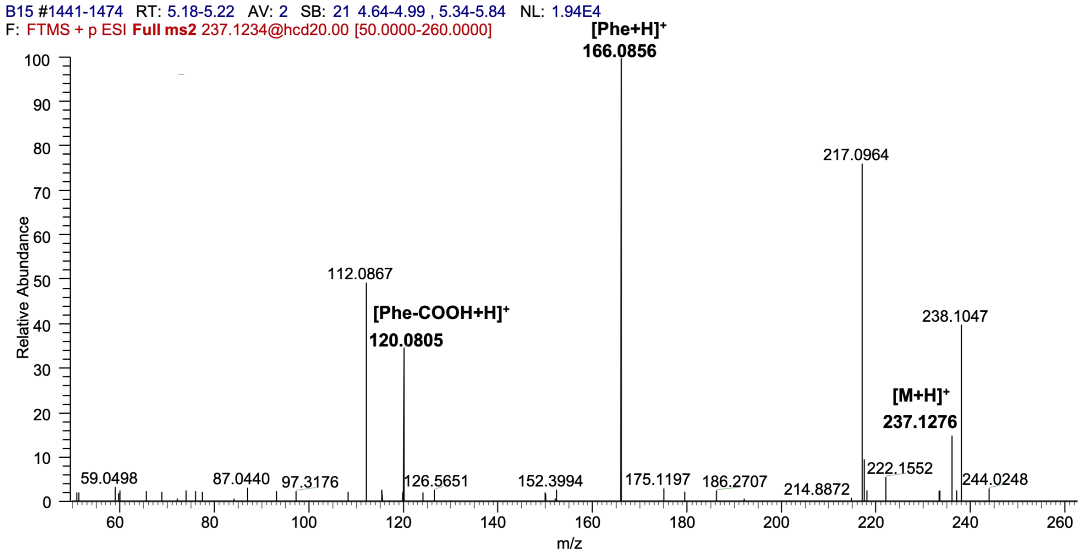

Supplementary Figure 16. The MS^2 spectrum of Ala-Phe dipeptide product. The dipeptide sequence is assigned by employing a comparative analysis of b/y ion pairs and characteristic fragments.

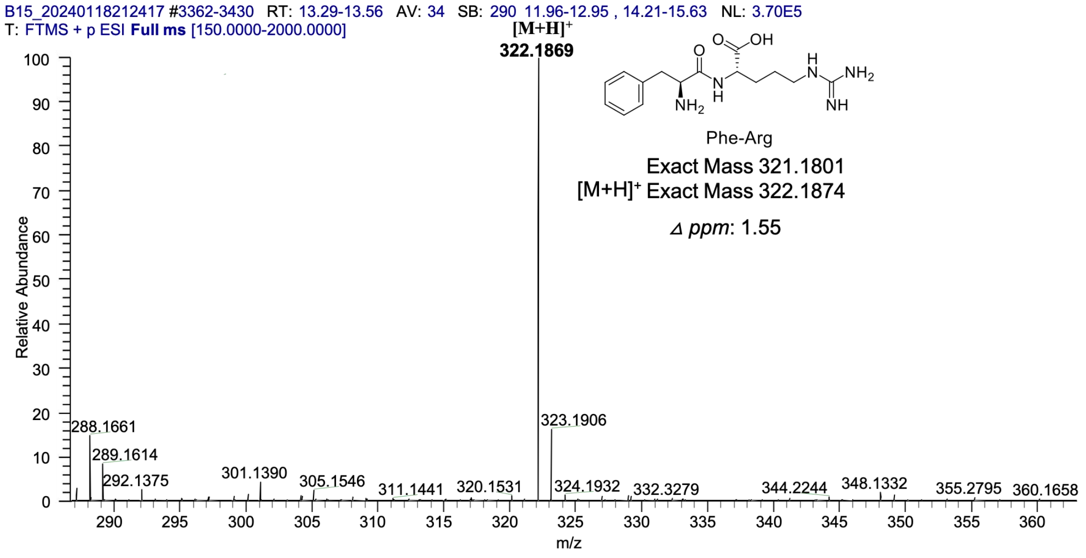

Supplementary Figure 17. The MS spectrum of Phe-Arg (*m/z* 322) dipeptide formed in the mixed amino acid (Ala, Phe and Arg) reaction systems with forsterite set up into Unit C3 with a total dose of 37.75 mGy onboard the CSS.

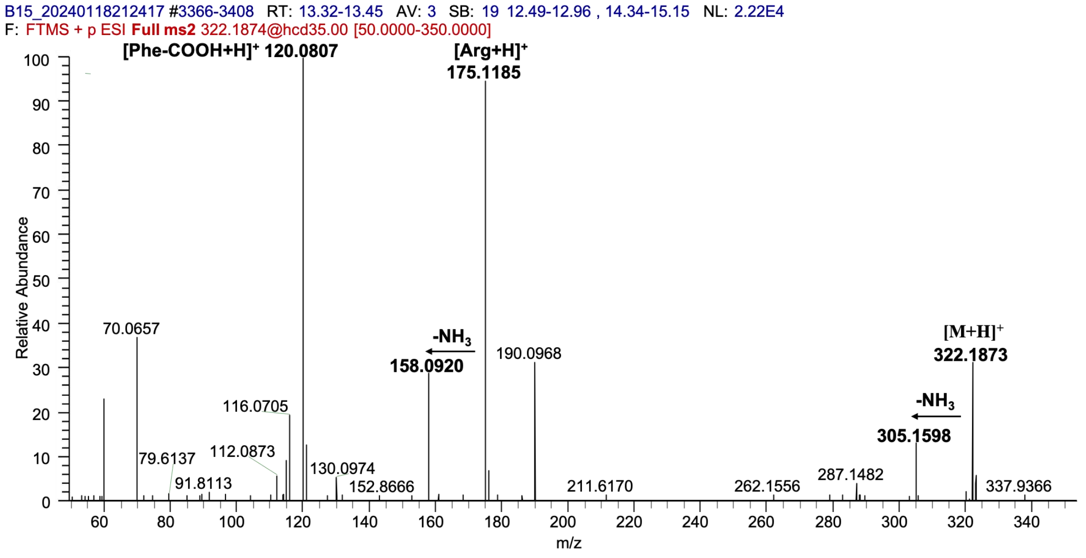

Supplementary Figure 18. The MS^2 spectrum of Phe-Arg. The dipeptide sequence is assigned by employing a comparative analysis of b/y ion pairs and characteristic fragments.

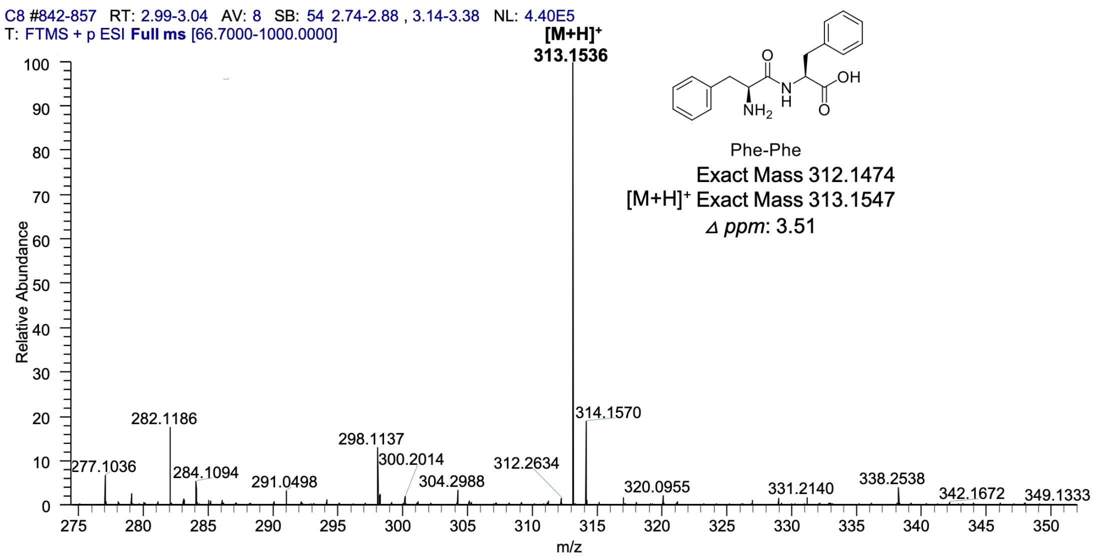

Supplementary Figure 19. The MS spectrum of Phe-Phe (*m/z* 313) dipeptide formed in the mixed amino acid (Ala, Phe and Arg) reaction systems without forsterite set up into Unit C3 with a total dose of 37.75 mGy onboard the CSS.

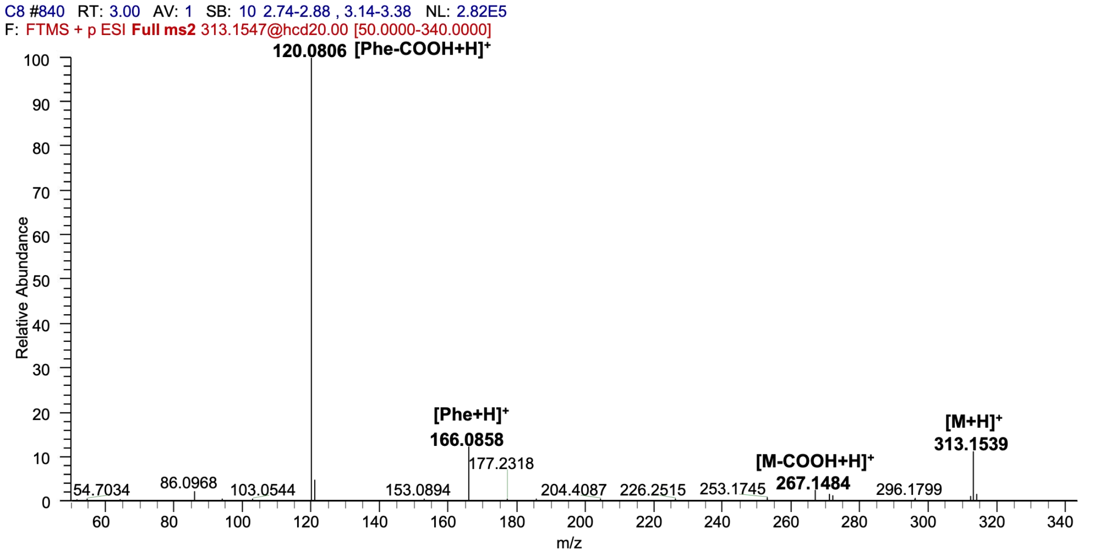

Supplementary Figure 20. The MS^2 spectrum of the Phe-Phe dipeptide product. The dipeptide sequence is assigned by employing a comparative analysis of b/y ion pairs and characteristic fragments.

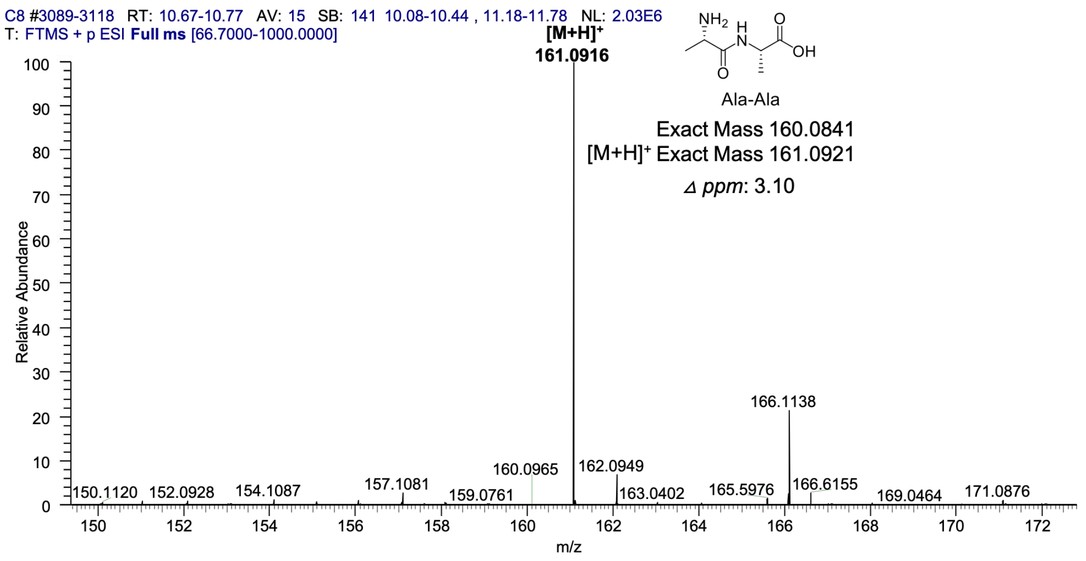

Supplementary Figure 21. The MS spectrum of Ala-Ala (*m/z* 161) dipeptide formed in the mixed amino acid (Ala, Phe and Arg) reaction systems without forsterite set up into Unit C3 with a total dose of 37.75 mGy onboard the CSS.

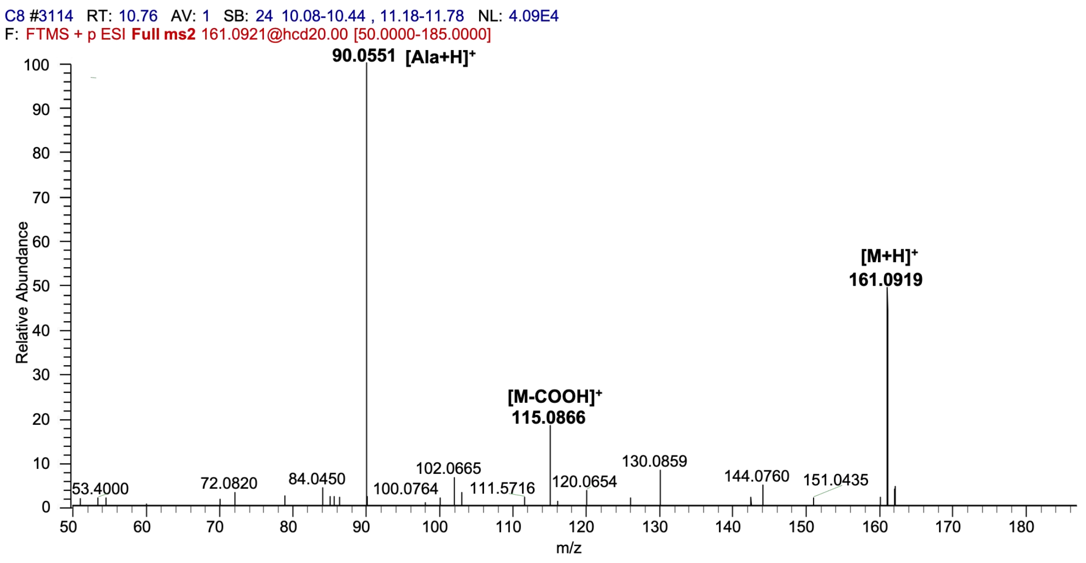

Supplementary Figure 22. The MS^2 spectrum of the Ala-Ala dipeptide product. The dipeptide sequence is assigned by employing a comparative analysis of b/y ion pairs and characteristic fragments.

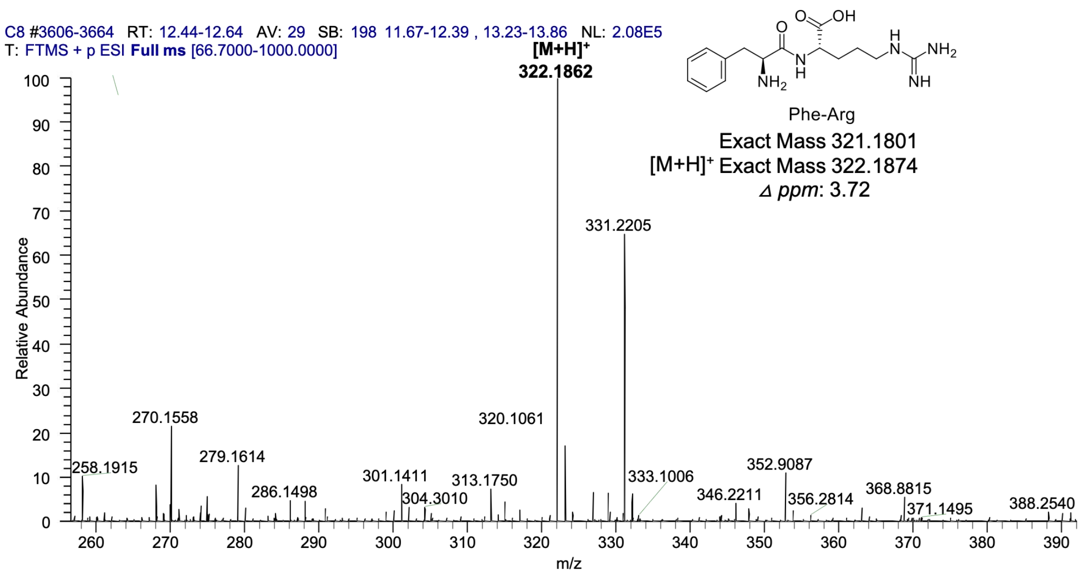

Supplementary Figure 23. The MS spectrum of Phe-Arg (*m/z* 322) dipeptide formed in the mixed amino acid (Ala, Phe and Arg) reaction systems without forsterite set up into Unit C3 with a total dose of 37.75 mGy onboard the CSS. (Blank control).

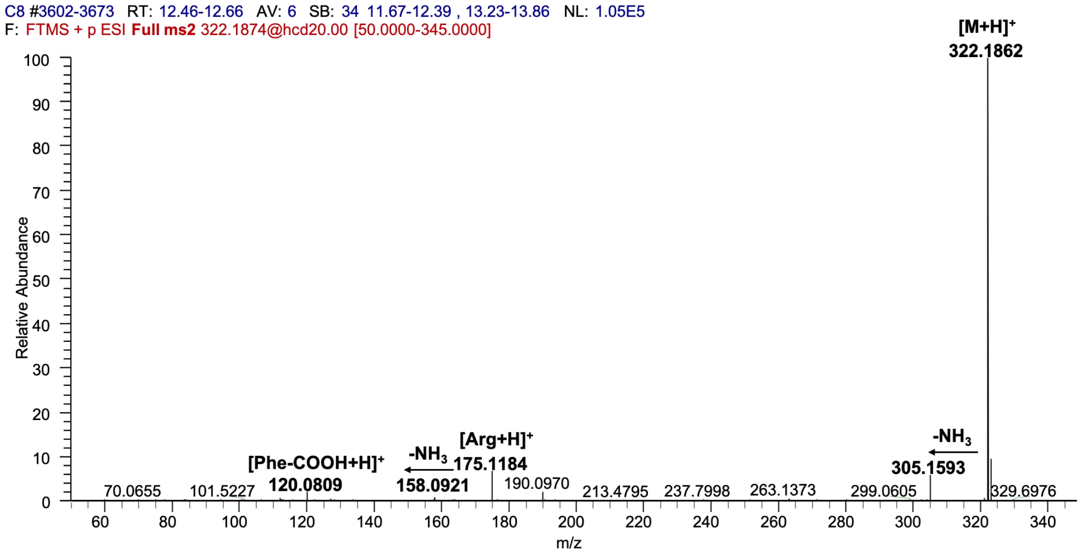

Supplementary Figure 24. The MS^2 spectrum of Phe-Arg dipeptide product. The dipeptide sequence is assigned by employing a comparative analysis of b/y ion pairs and characteristic fragments.

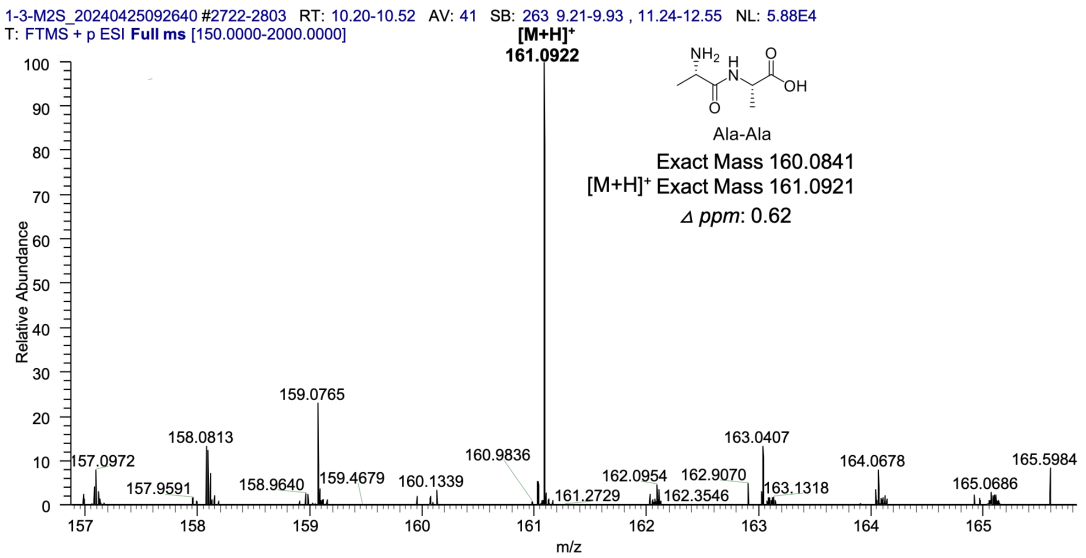

Supplementary Figure 25. The MS spectrum of Ala-Ala (*m/z* 161) product obtained in the mixed amino acid reaction system (Ala, Phe and Arg) under a radiation dose of 63.63 mGy on the ground using forsterite as an aid (Control 2).

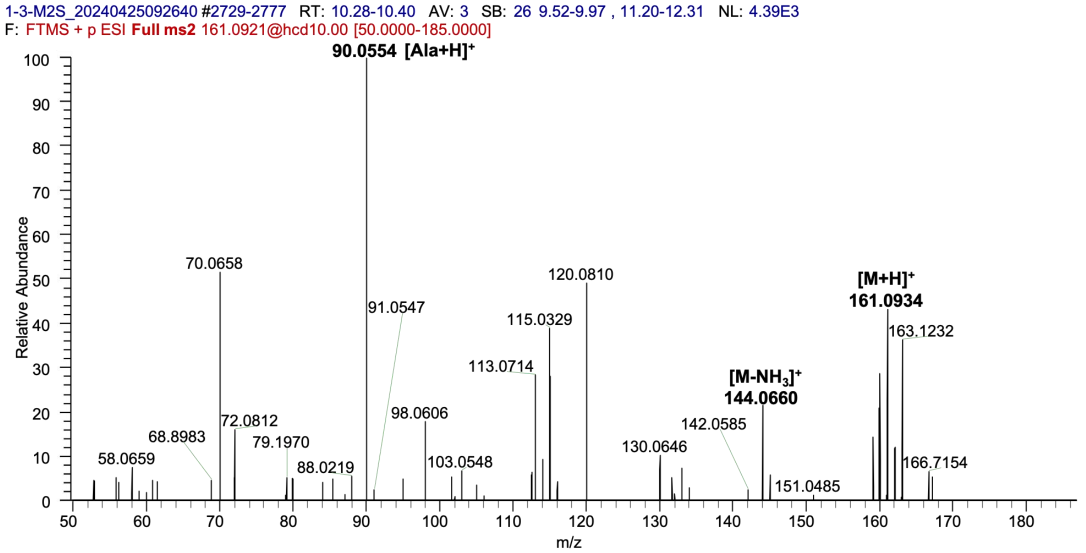

Supplementary Figure 26. The MS^2 spectrum of the Ala-Ala $[M+H]^+$ ion (*m/z* 161.09) in **Supplementary Figure 25.**

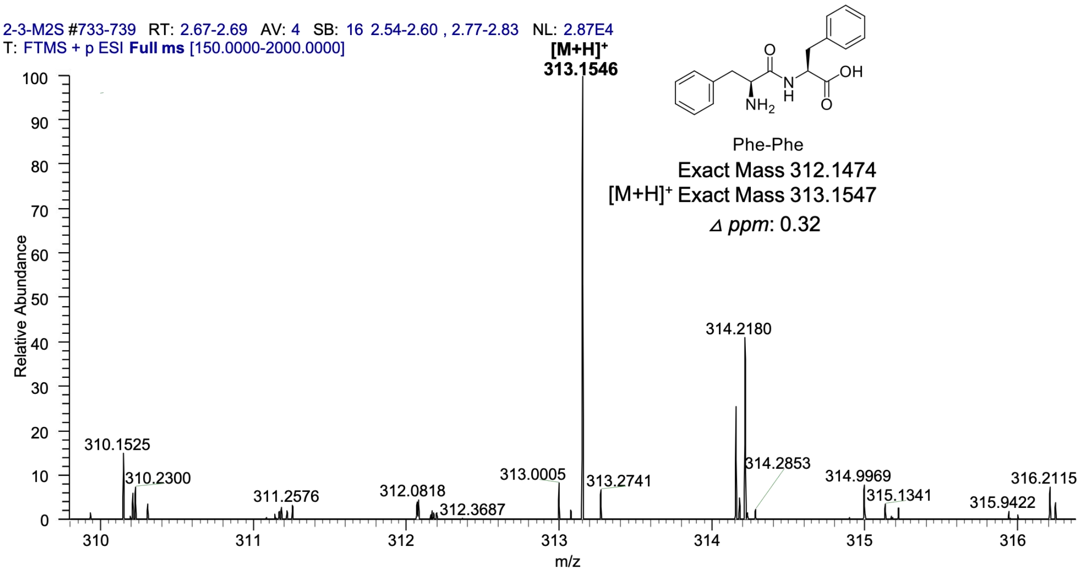

Supplementary Figure 27. The MS spectrum of the Phe-Phe (*m/z* 313) product obtained in the mixed amino acid reaction system (Ala, Phe and Arg) under a radiation dose of 1 Gy on the ground using forsterite as an aid (Control 3).

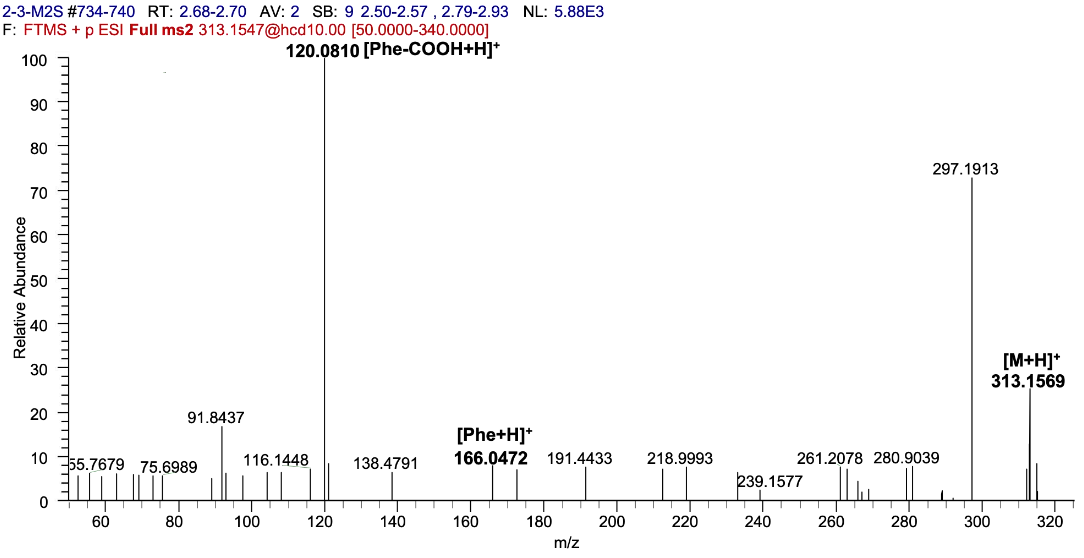

Supplementary Figure 28. The MS^2 spectrum of Phe-Phe $[M+H]^+$ ion (*m/z* 313.15) in **Supplementary Figure 27**.

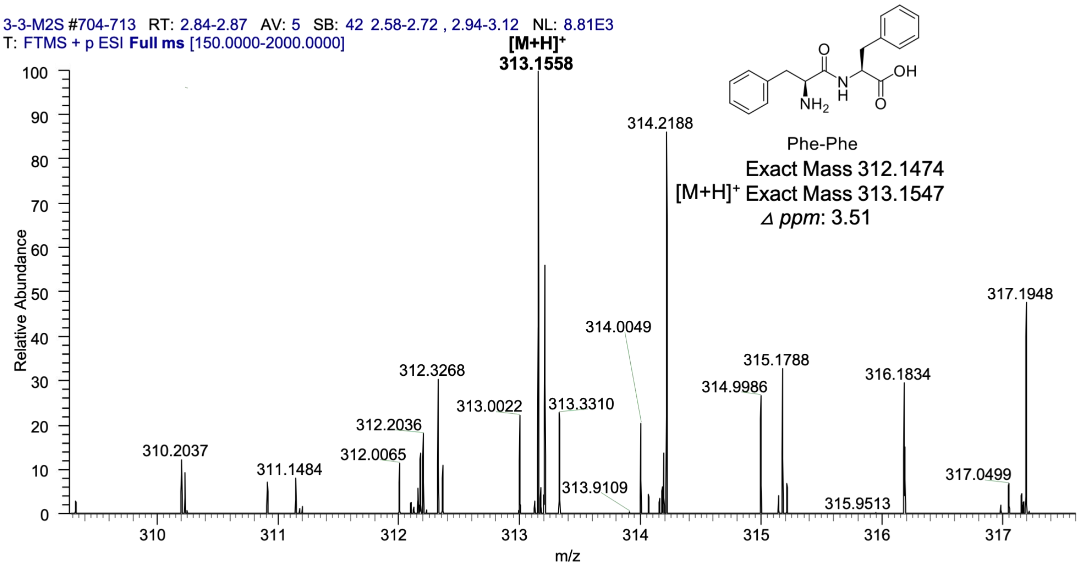

Supplementary Figure 29. The MS spectrum of the Phe-Phe (*m/z* 313) product obtained in the mixed amino acid reaction system (Ala, Phe and Arg) under a radiation dose of 10 Gy on the ground using forsterite as an aid (Control 4).

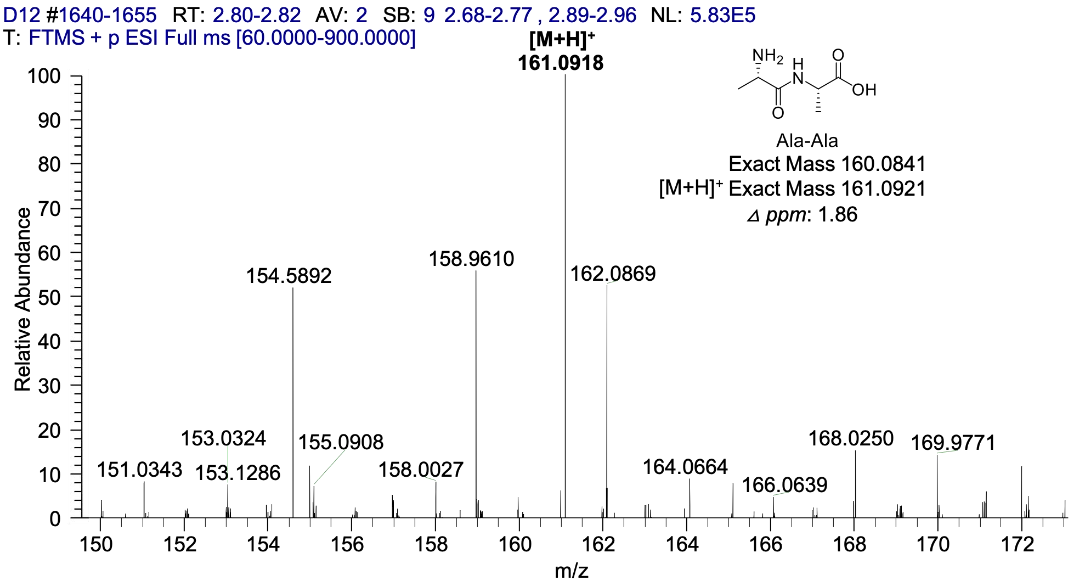

Supplementary Figure 30. The MS spectrum of Ala-Ala (*m/z* 161) dipeptide formed in the mixed amino acid (Ala, Phe and Arg) reaction systems with forsterite set up into Unit B3 with a total dose of 101.08 mGy onboard the CSS.

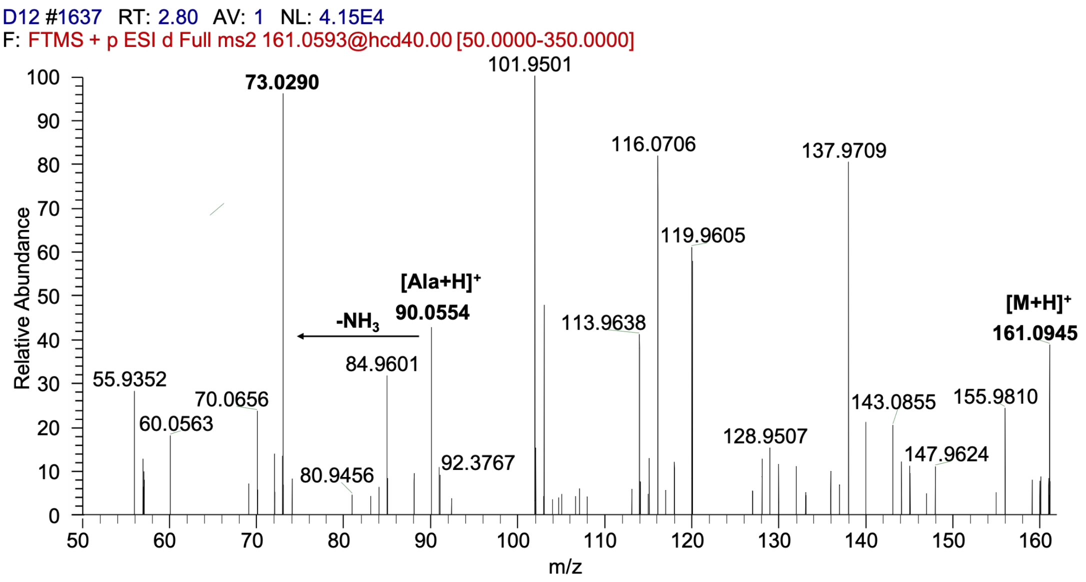

Supplementary Figure 31. The MS^2 spectrum of Ala-Ala $[M+H]^+$ ion (*m/z* 161.09) in **Supplementary Figure 30**.

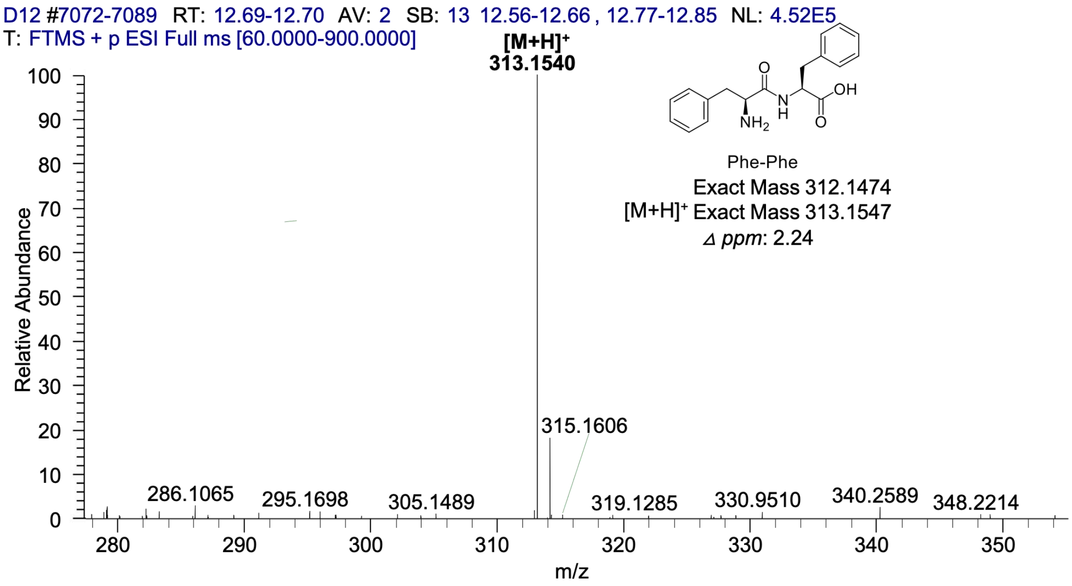

Supplementary Figure 32. The MS spectrum of Phe-Phe (*m/z* 313) dipeptide formed in the mixed amino acid (Ala, Phe and Arg) reaction systems with forsterite set up into Unit B3 with a total dose of 101.08 mGy onboard the CSS.

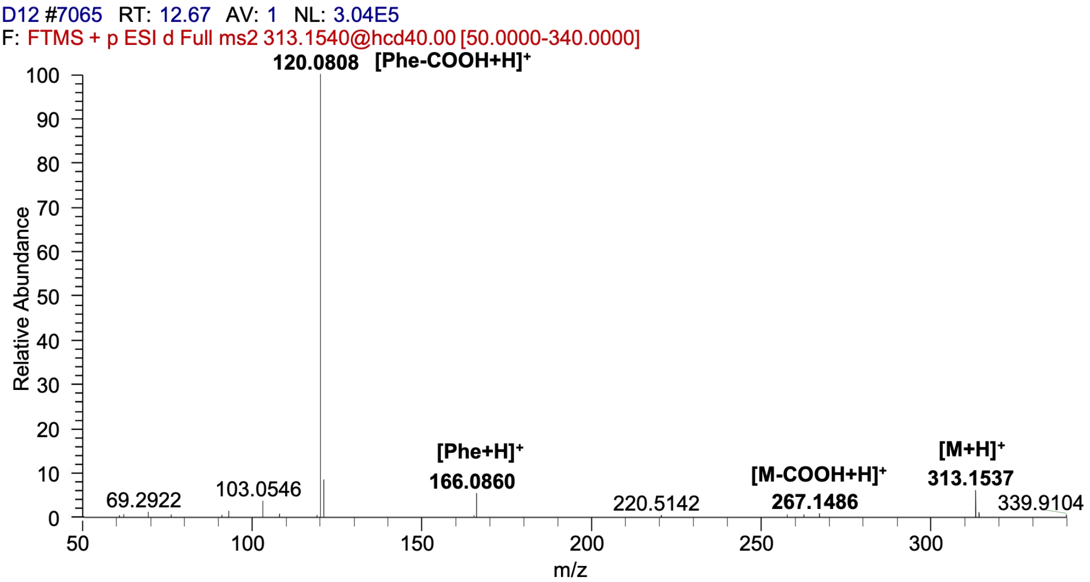

Supplementary Figure 33. The MS^2 spectrum of Phe-Phe $[M+H]^+$ ion (*m/z* 313.15) in **Supplementary Figure 32**.

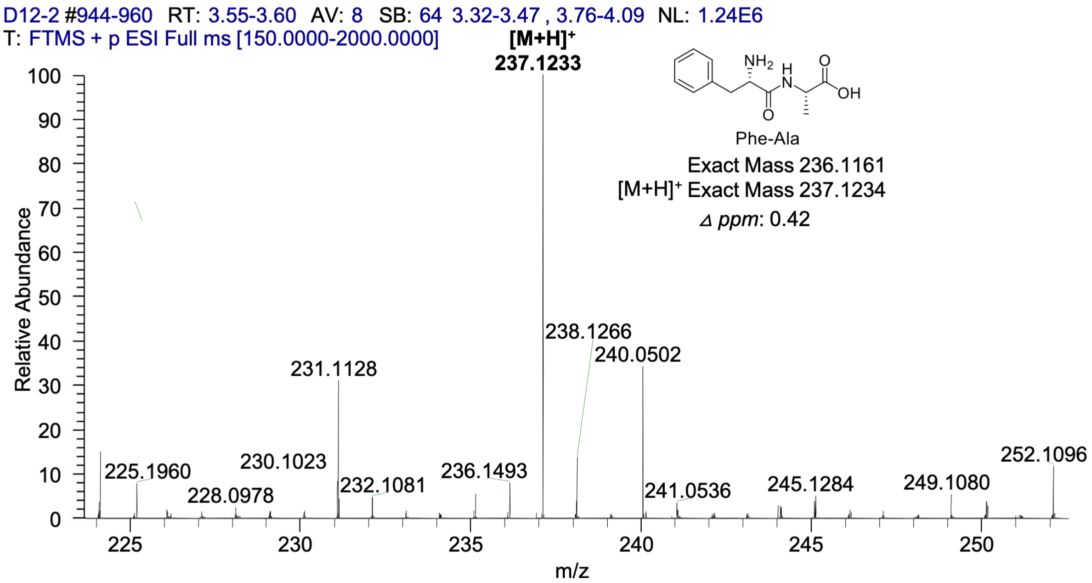

Supplementary Figure 34. The MS spectrum of Phe-Ala (*m/z* 237) dipeptide formed in the mixed amino acid (Ala, Phe and Arg) reaction systems with forsterite set up into Unit B3 with a total dose of 101.08 mGy onboard the CSS.

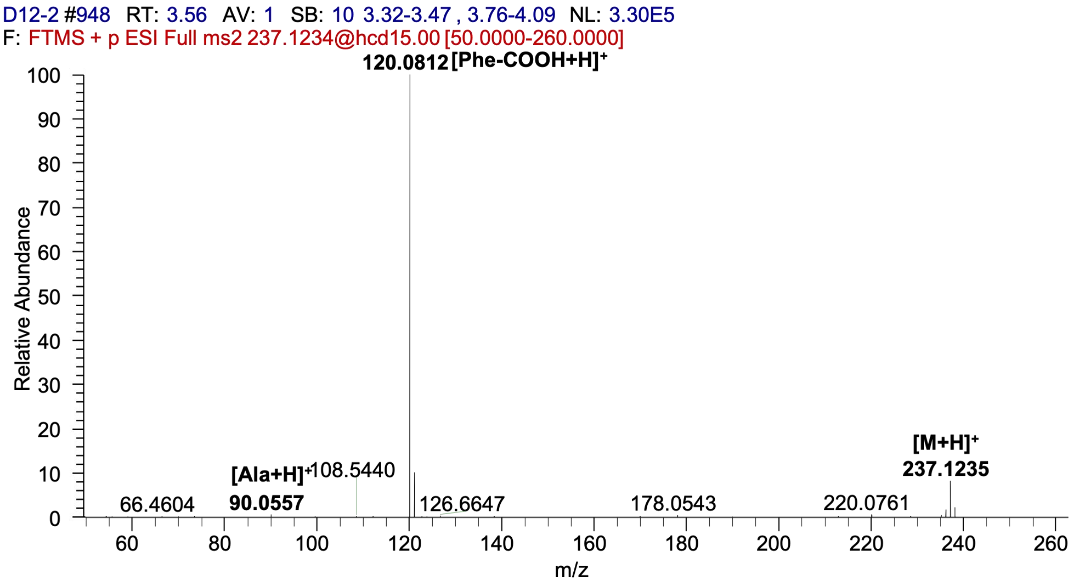

Supplementary Figure 35. The MS^2 spectrum of Phe-Ala $[M+H]^+$ ion (*m/z* 237.12) in **Supplementary Figure 34**.

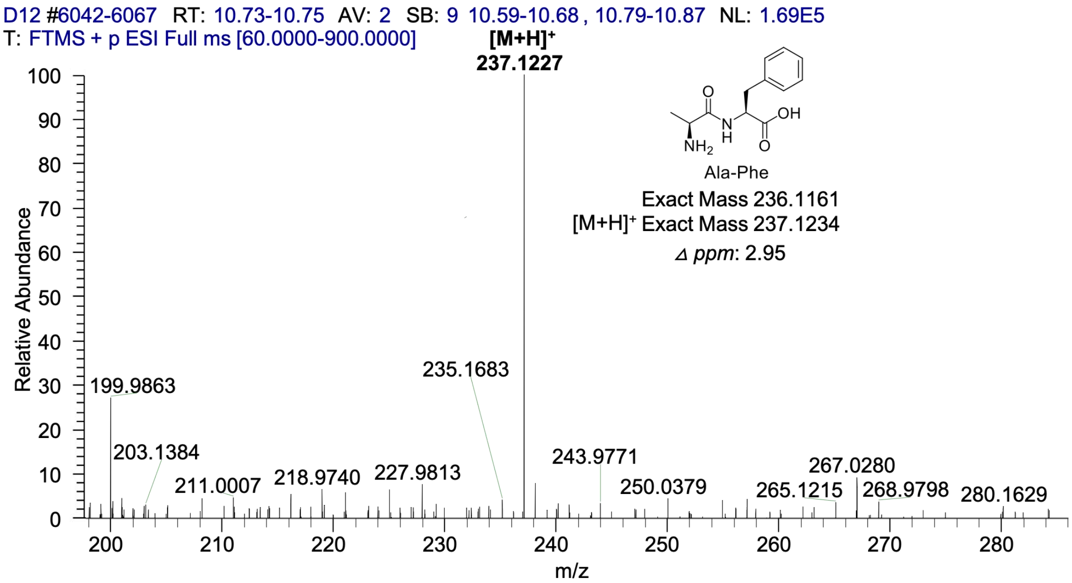

Supplementary Figure 36. The MS spectrum of Ala-Phe (*m/z* 237) dipeptide formed in the mixed amino acid (Ala, Phe and Arg) reaction systems with forsterite set up into Unit B3 with a total dose of 101.08 mGy onboard the CSS.

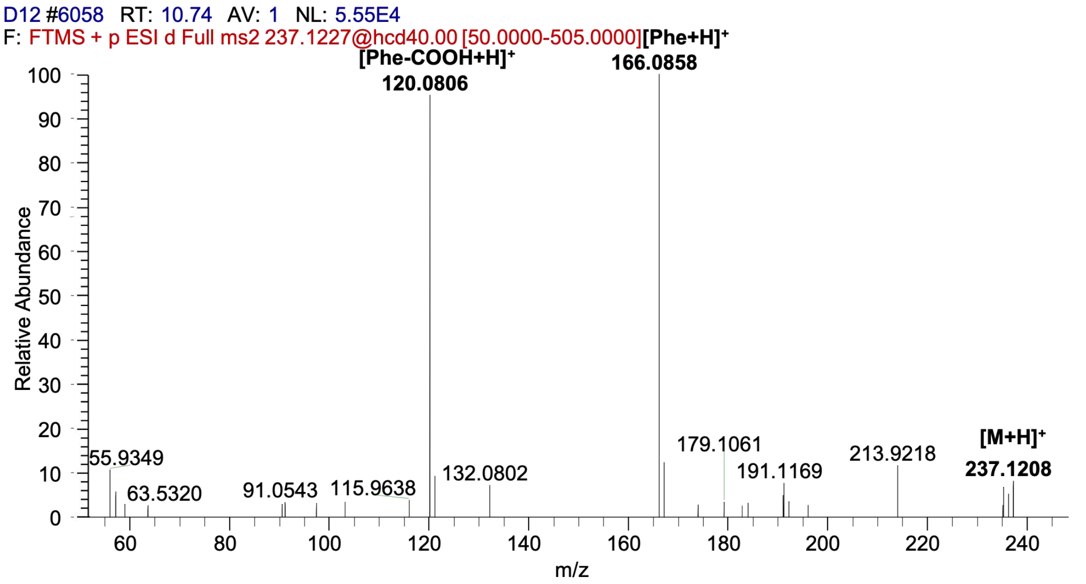

Supplementary Figure 37. The MS^2 spectrum of Ala-Phe $[M+H]^+$ ion (*m/z* 237.12) in **Supplementary Figure 36**.

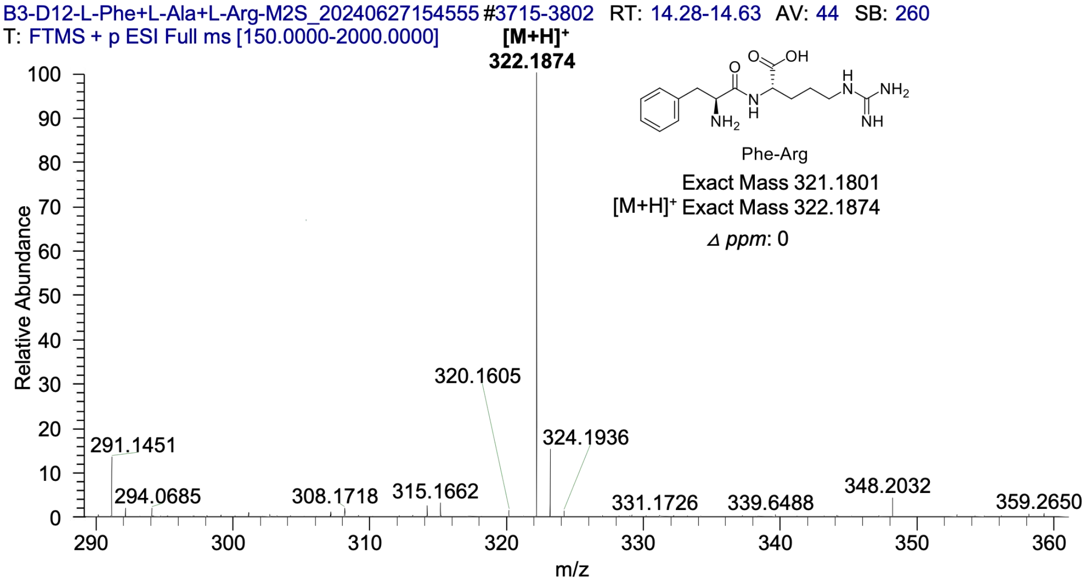

Supplementary Figure 38. The MS spectrum of Phe-Arg (*m/z* 322) dipeptide formed in the mixed amino acid (Ala, Phe and Arg) reaction systems with forsterite set up into Unit B3 with a total dose of 101.08 mGy onboard the CSS.

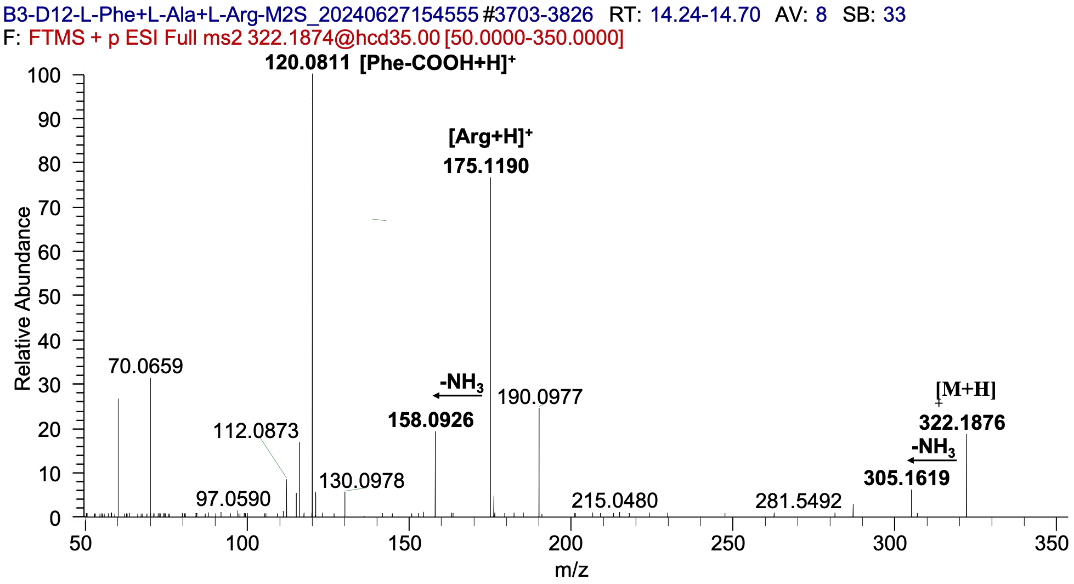

Supplementary Figure 39. The MS^2 spectrum of Phe-Arg $[M+H]^+$ ion (*m/z* 322.18) in **Supplementary Figure 38**.

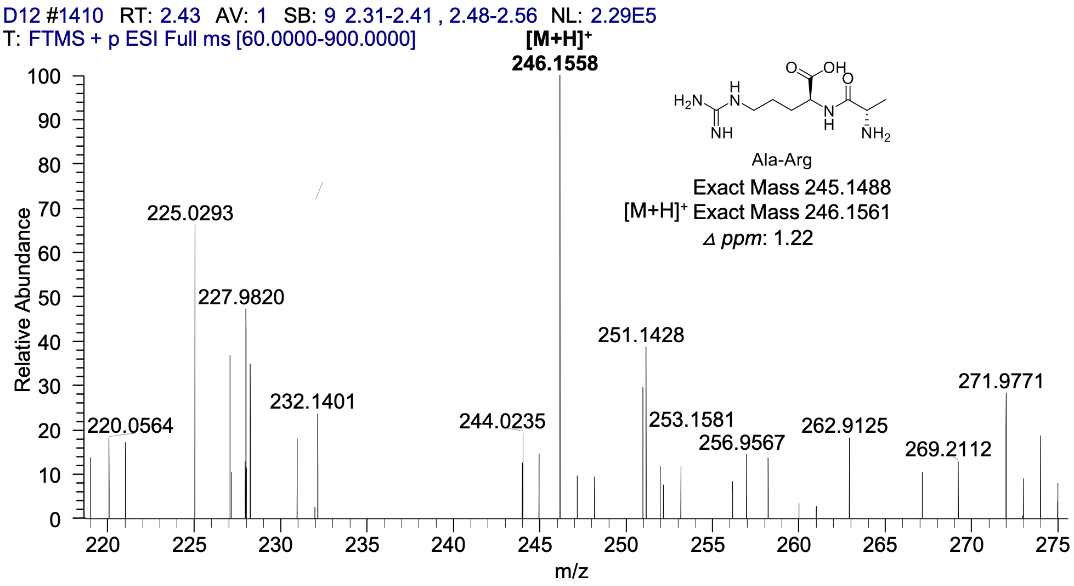

Supplementary Figure 40. The MS spectrum of Ala-Arg (*m/z* 246) dipeptide formed in the mixed amino acid (Ala, Phe and Arg) reaction systems with forsterite set up into Unit B3 with a total dose of 101.08 mGy onboard the CSS.

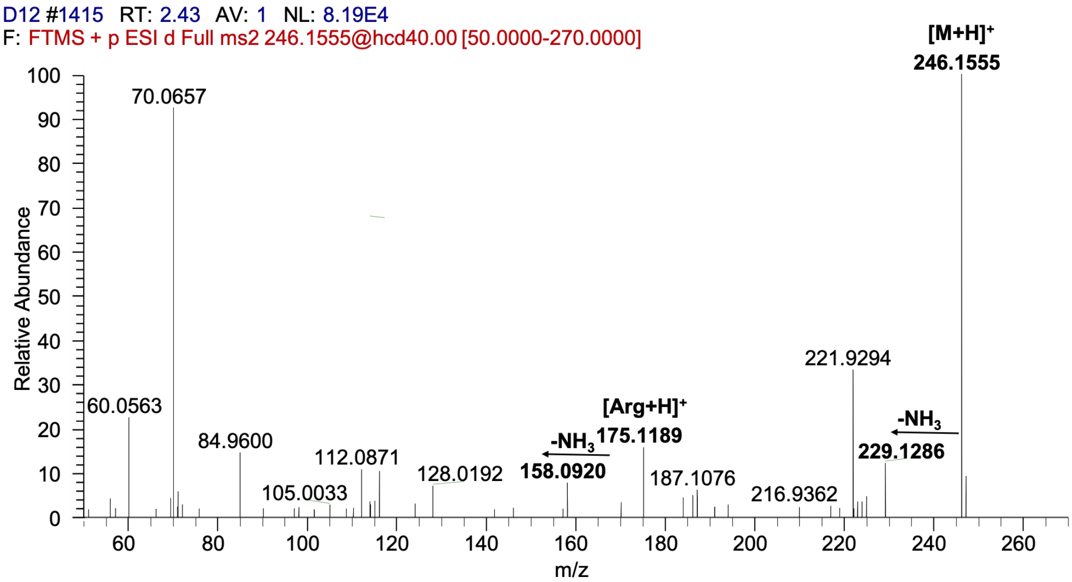

Supplementary Figure 41. The MS^2 spectrum of Ala-Arg $[M+H]^+$ ion (*m/z* 246.15) in **Supplementary Figure 40**.

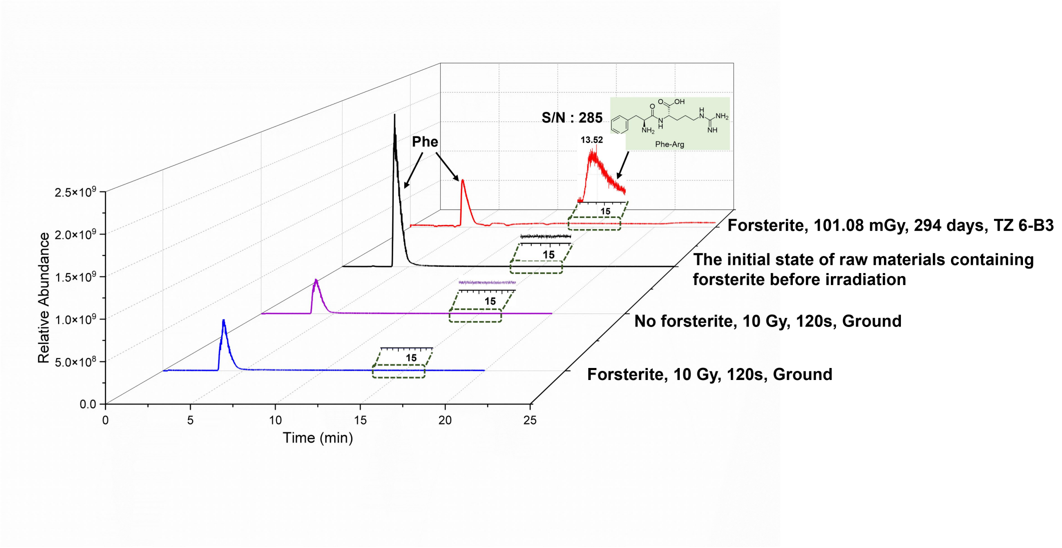

Supplementary Figure 42. The extracted ion chromatogram (EIC) of reactant raw materials (Phe and Ala) and reaction product (e.g., Phe-Arg) dipeptide formed in the mixed amino acid (Ala, Phe, and Arg) reaction systems setting up in Unit B3 with the assistance of forsterite and a total dose of 101.08 mGy onboard the CSS, with the assistance of forsterite and a total dose of 10 Gy on the ground, without forsterite and a total dose of 10 Gy on the ground and with the assistance of forsterite but without radiation on the ground. The relevant products were detectable in all ground samples, irrespective of the presence of radiation or forsterite.

8. MS analysis of NMP standard substance

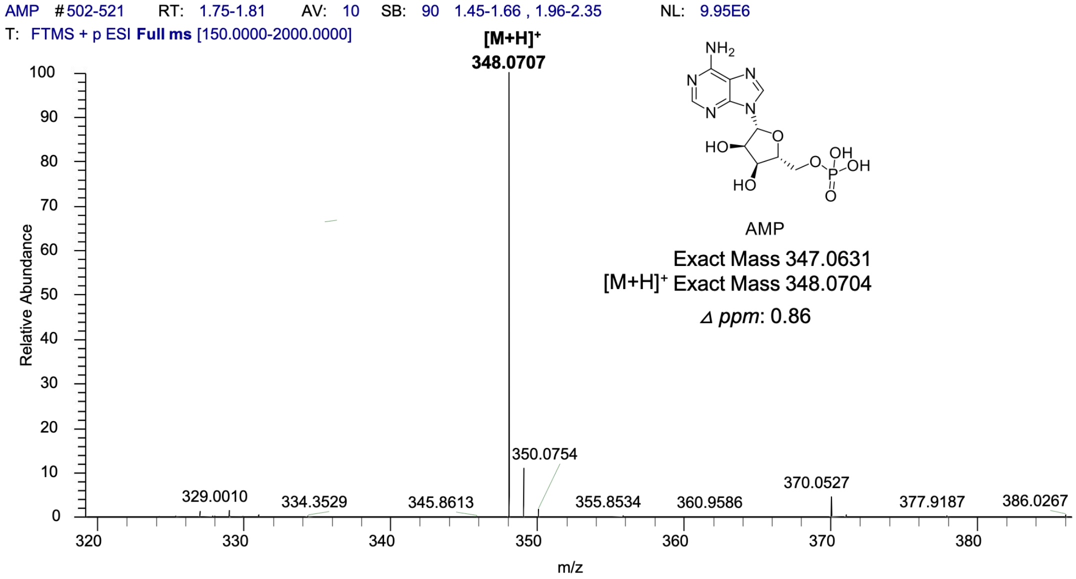

Supplementary Figure 43. The MS spectrum of 5'-AMP standard substance (*m/z* 348.07).

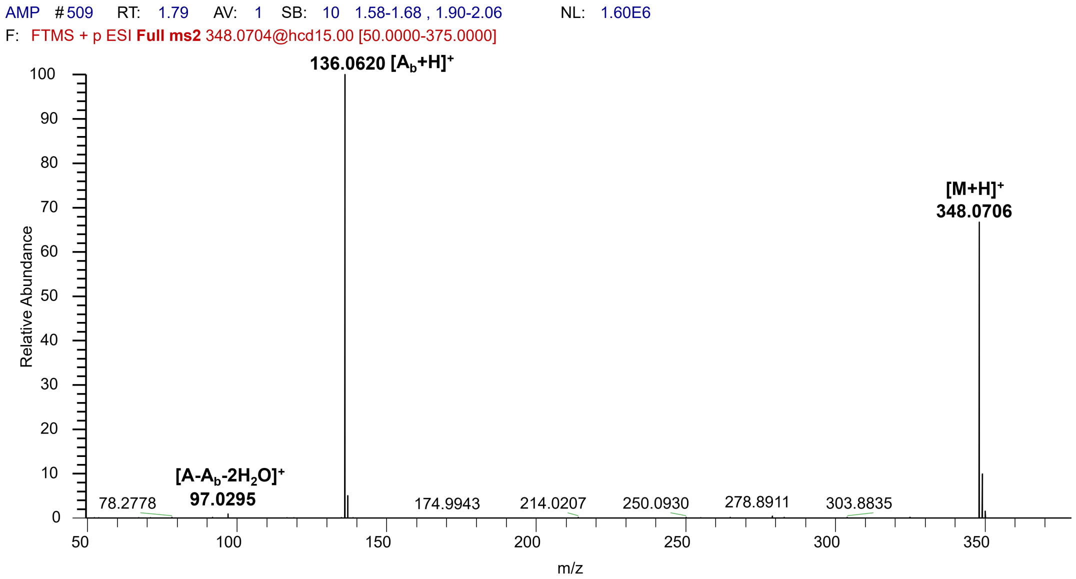

Supplementary Figure 44. The MS^2 spectrum of 5'-AMP standard substance (*m/z* 348.07). A: Adenosine; A_b: The base adenine.

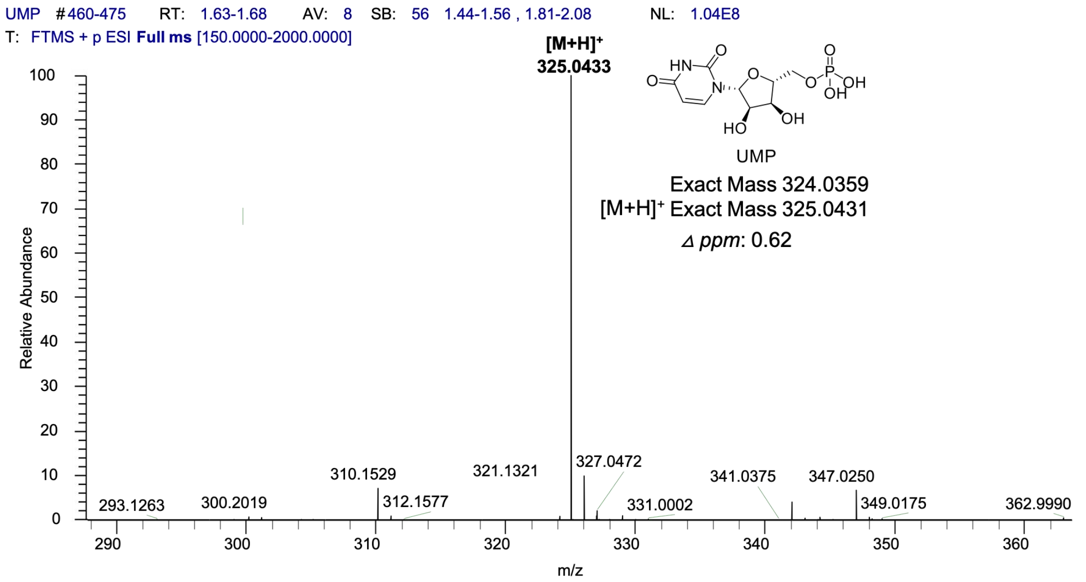

Supplementary Figure 45. The MS spectrum of 5'-UMP standard substance (*m/z* 325.04).

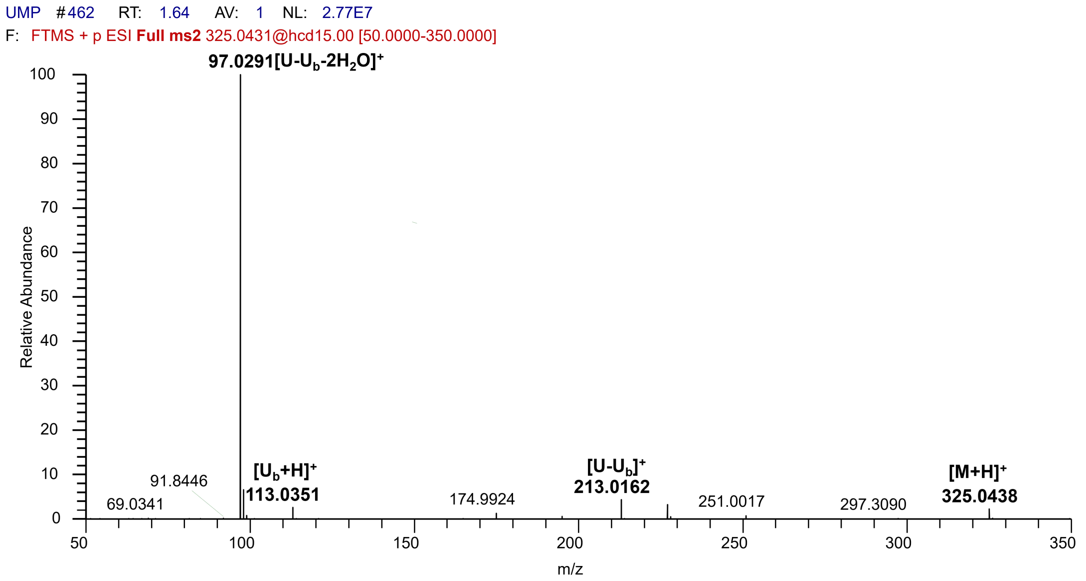

Supplementary Figure 46. The MS^2 spectrum of 5'-UMP standard substance (*m/z* 325.04). U: Uridine; U_b: The base uracil.

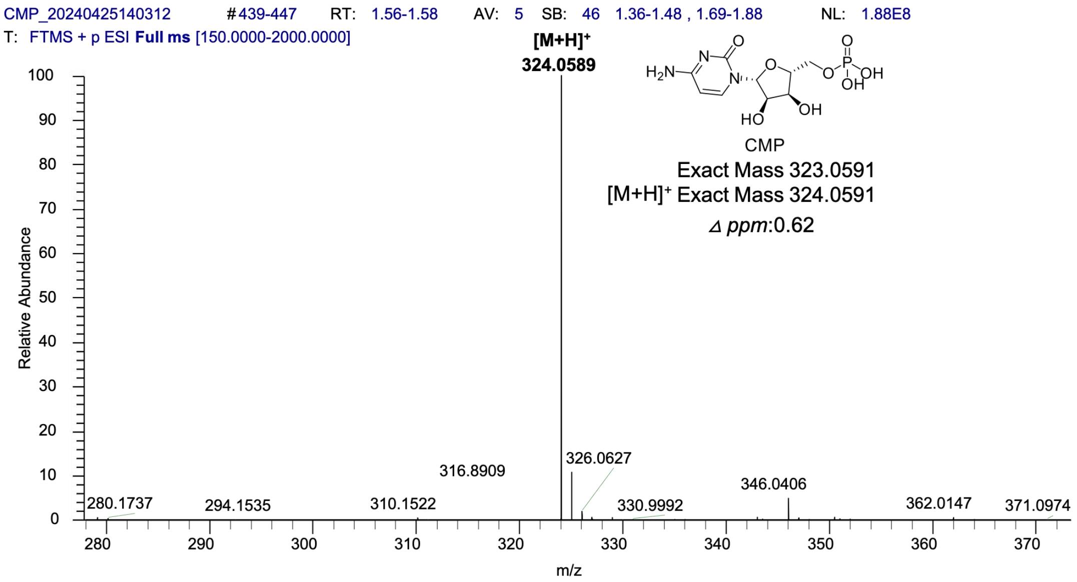

Supplementary Figure 47. The MS spectrum of 5'-CMP standard substance (*m/z* 324.06).

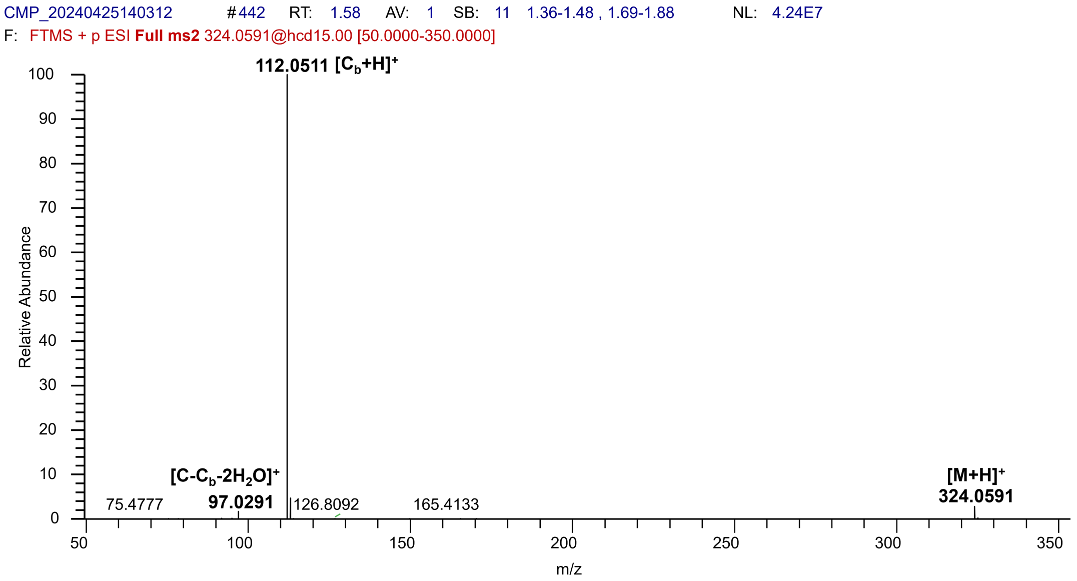

Supplementary Figure 48. The MS^2 spectrum of 5'-CMP standard substance (*m/z* 324.06). C: Cytidine; C_b: The base cytosine.

9. MS analysis of NMP in the mixed amino acid, nucleosides and P3m reaction system under the space radiation condition in C3 Unit of TZ 6

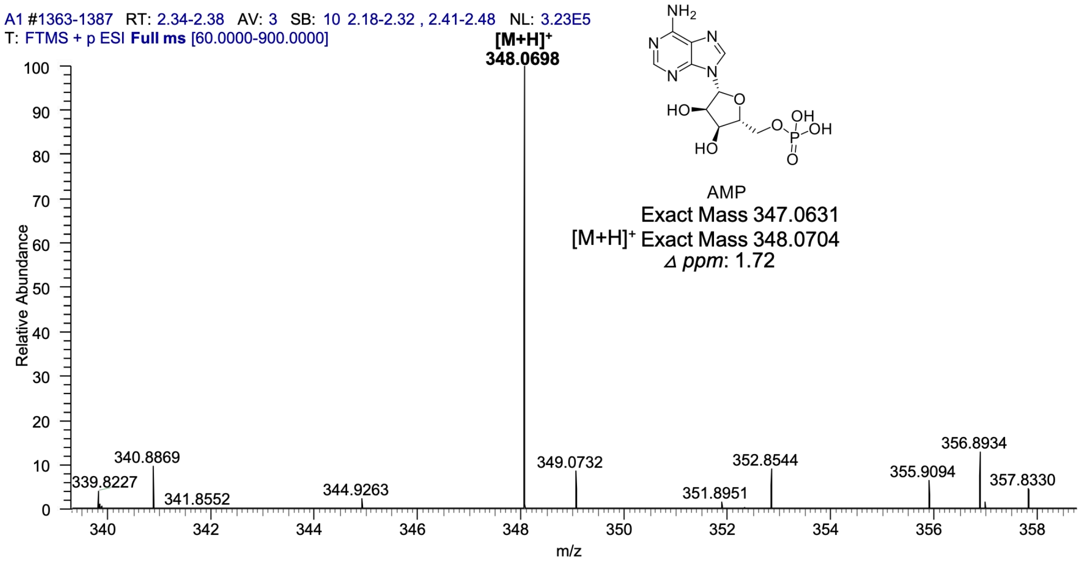

Supplementary Figure 49. The MS spectrum of 5'-AMP (*m/z* 348) product formed from the reaction system containing Phe, nucleosides (A and U) and P3m without minerals under the CSS radiation with a total dose of 37.75 mGy in C3 Unit.

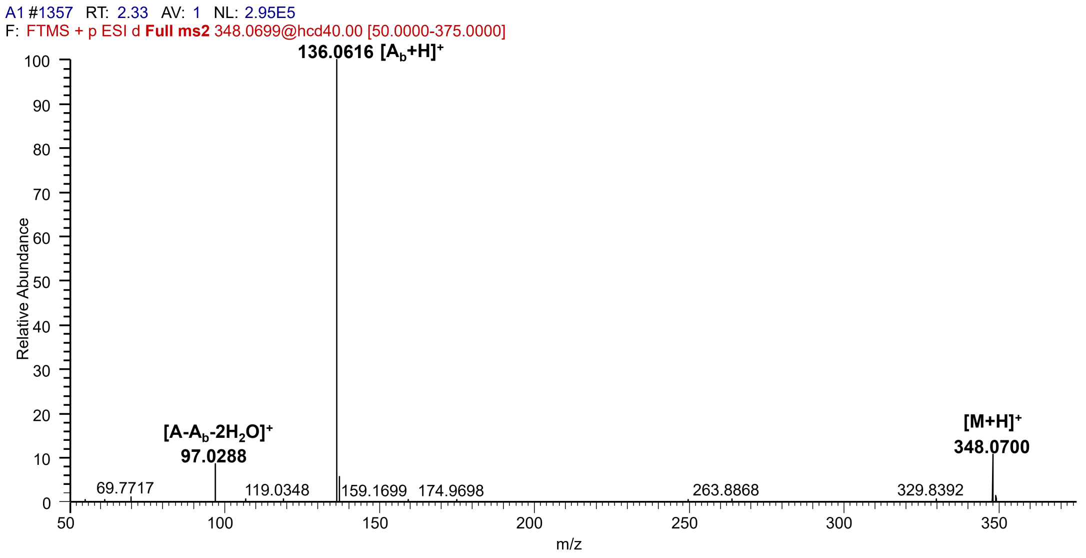

Supplementary Figure 50. The MS^2 spectrum of 5'-AMP $[M+H]^+$ ion (*m/z* 348.07) in **Supplementary Figure 49**. A: Adenosine; A_b: The base adenine.

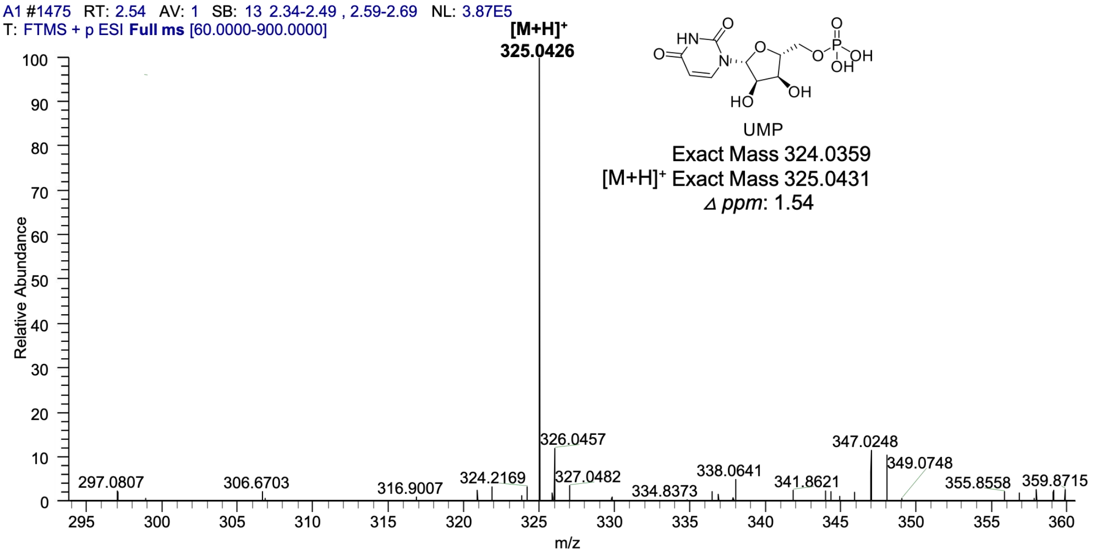

Supplementary Figure 51. The MS spectrum of 5'-UMP (*m/z* 325) product formed from the reaction system containing Phe, nucleosides (A and U) and P_3m without minerals under the CSS radiation, resulting in a total dose of 37.75 mGy in C3 Unit.

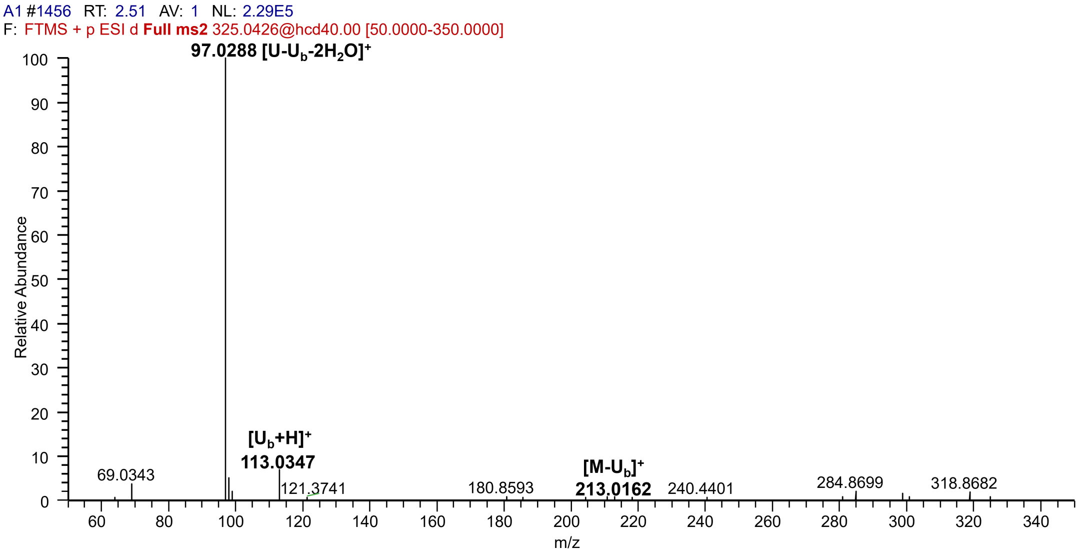

Supplementary Figure 52. The MS^2 spectrum of 5'-UMP $[M+H]^+$ ion (*m/z* 325.04) in **Supplementary Figure 51**. U: Uridine; U_b: The base uracil.

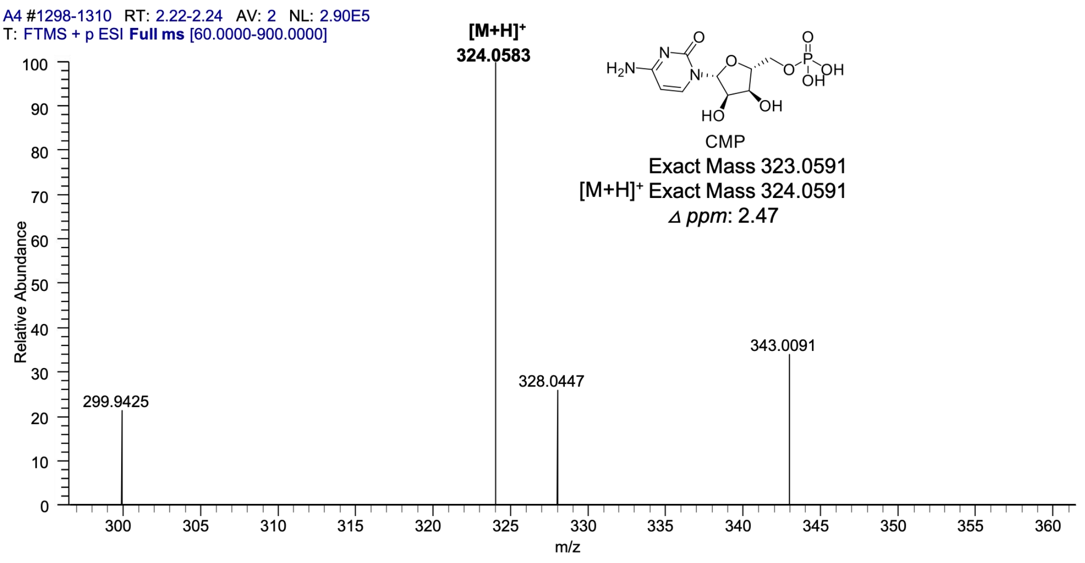

Supplementary Figure 53. The MS spectrum of 5'-CMP (*m/z* 324.06) product formed from the reaction system containing Phe, nucleosides (C and G) and P3m without minerals under the CSS radiation with a total dose of 37.75 mGy in C3 Unit.

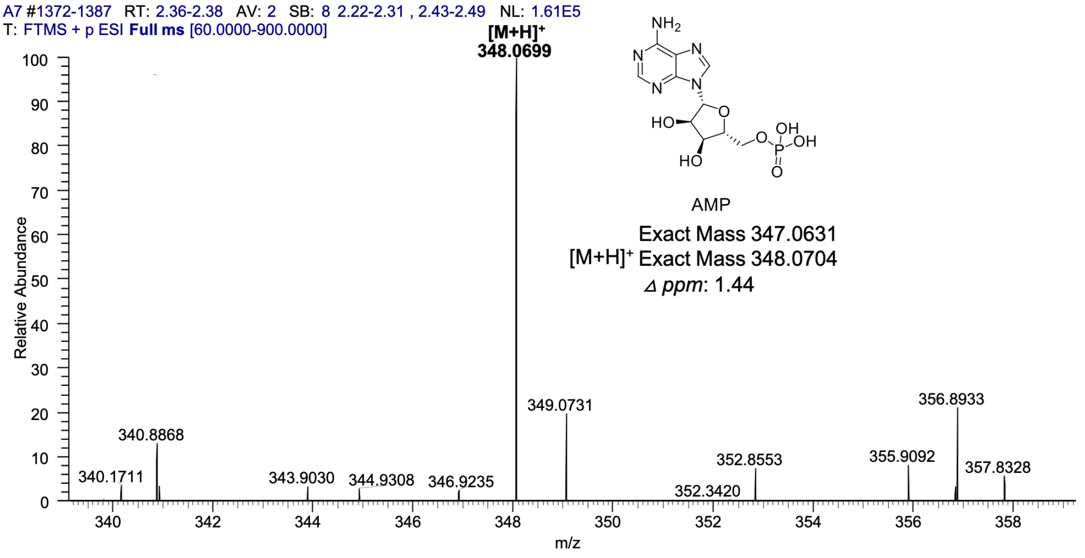

Supplementary Figure 54. The MS spectrum of 5'-AMP (*m/z* 348.07) product formed from the reaction system containing Ala, nucleosides (A and U) and P3m without minerals under the CSS radiation with a total dose of 37.75 mGy in C3 Unit.

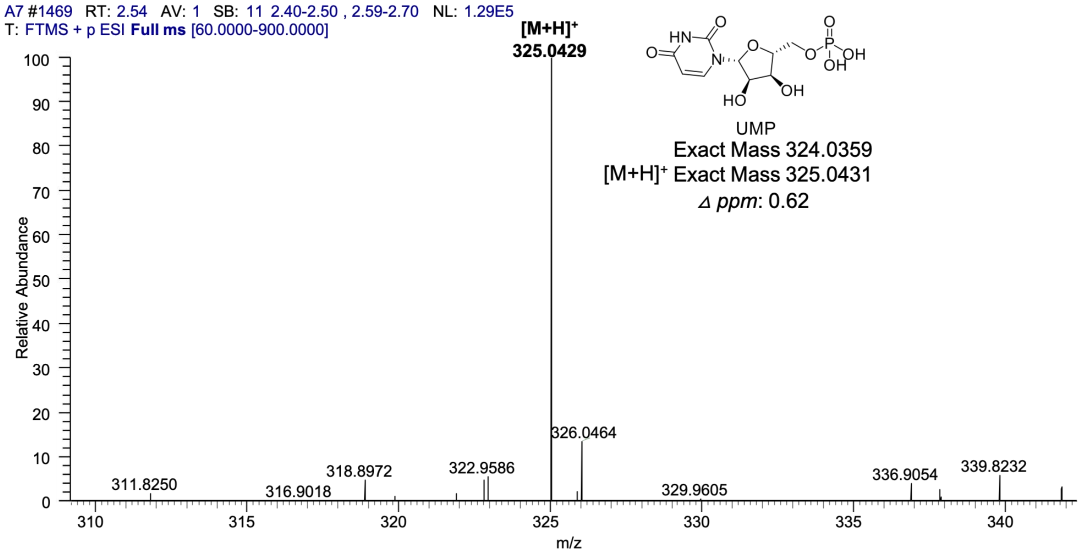

Supplementary Figure 55. The MS spectrum of 5'-UMP (*m/z* 325.04) product formed from the reaction system containing Ala, nucleosides (A and U) and P3m without minerals under the CSS radiation resulting in a total dose of 37.75 mGy in C3 Unit.

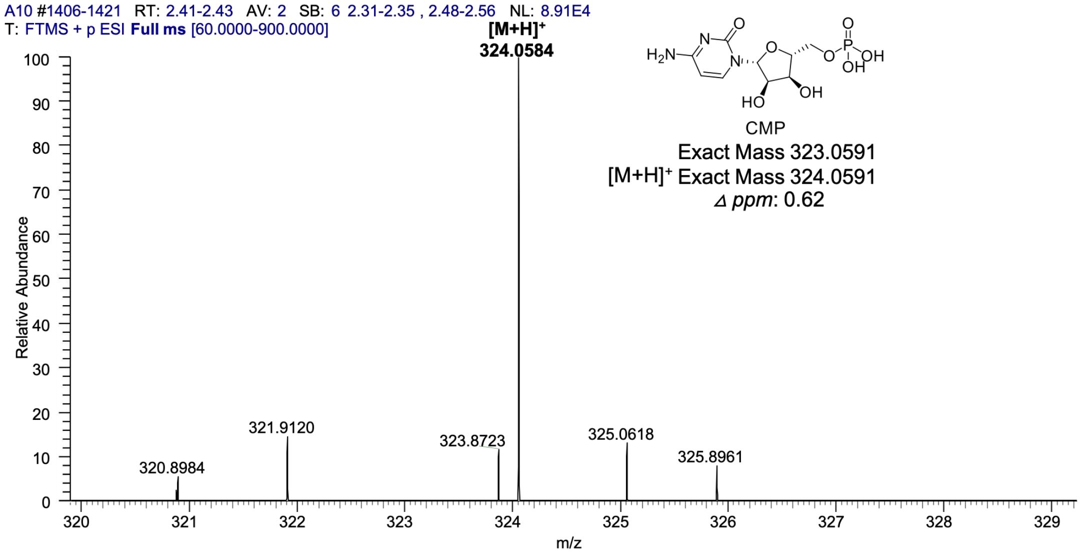

Supplementary Figure 56. The MS spectrum of 5'-CMP (*m/z* 324.06) product formed from the reaction system containing Ala, nucleosides (C and G) and P3m without minerals under the CSS radiation with a total dose of 37.75 mGy in C3 Unit.

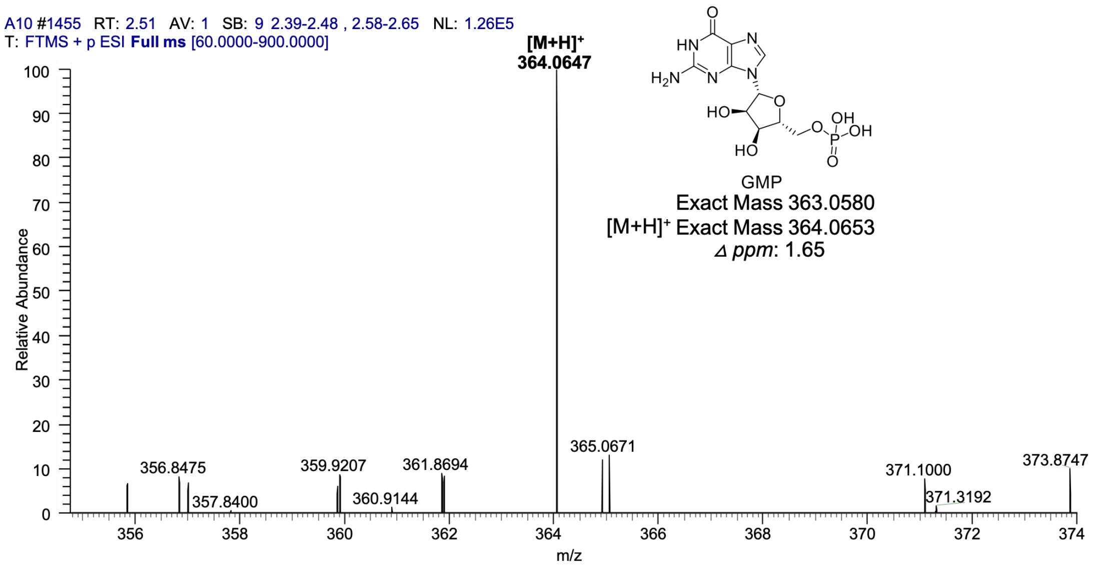

Supplementary Figure 57. The MS spectrum of 5’-GMP (*m/z* 364.06) product formed from the reaction system containing Ala, nucleosides (C and G) and P3m without minerals under the CSS radiation with a total dose of 37.75 mGy in C3 Unit.

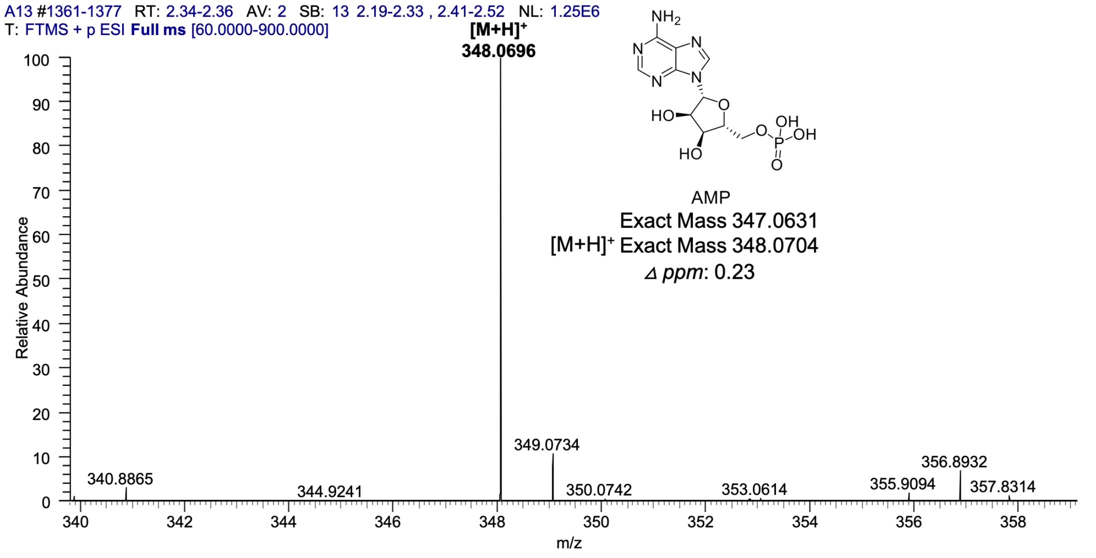

Supplementary Figure 58. The MS spectrum of 5’-AMP (*m/z* 348.07) product formed in the reaction system containing Arg, nucleosides (A and U) and P3m without minerals under the CSS radiation with a total dose of 37.75 mGy in C3 Unit.

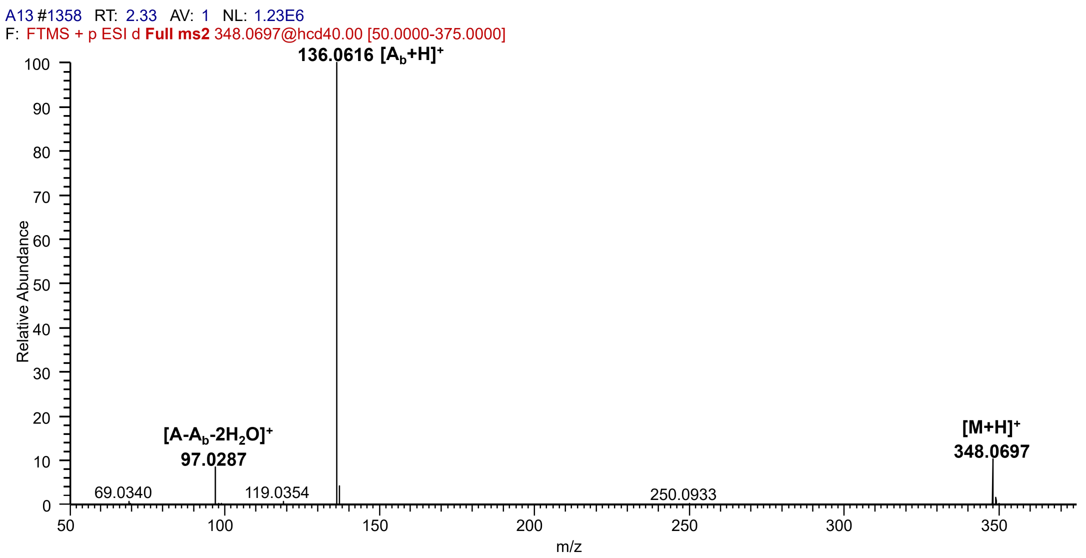

Supplementary Figure 59. The MS^2 spectrum of 5'-AMP $[M+H]^+$ ion (*m/z* 348.07) in **Supplementary Figure 58**. A: Adenosine; A_b: The base adenine.

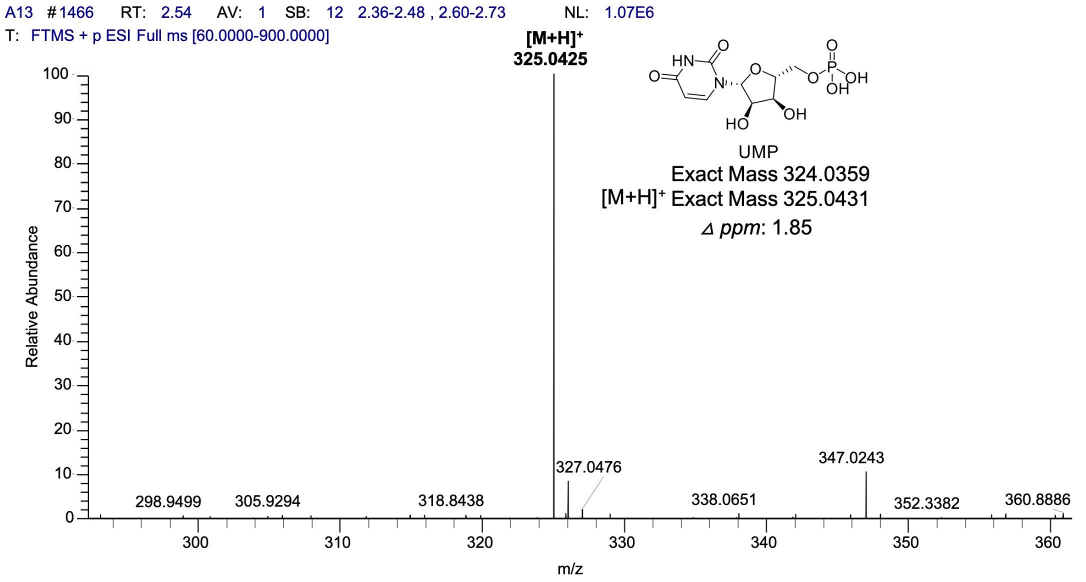

Supplementary Figure 60. The MS spectrum of 5'-UMP (*m/z* 325.04) product formed from the reaction system containing Arg, nucleosides (A and U) and P_3m without minerals under the CSS radiation with a total dose of 37.75 mGy in C3 Unit.

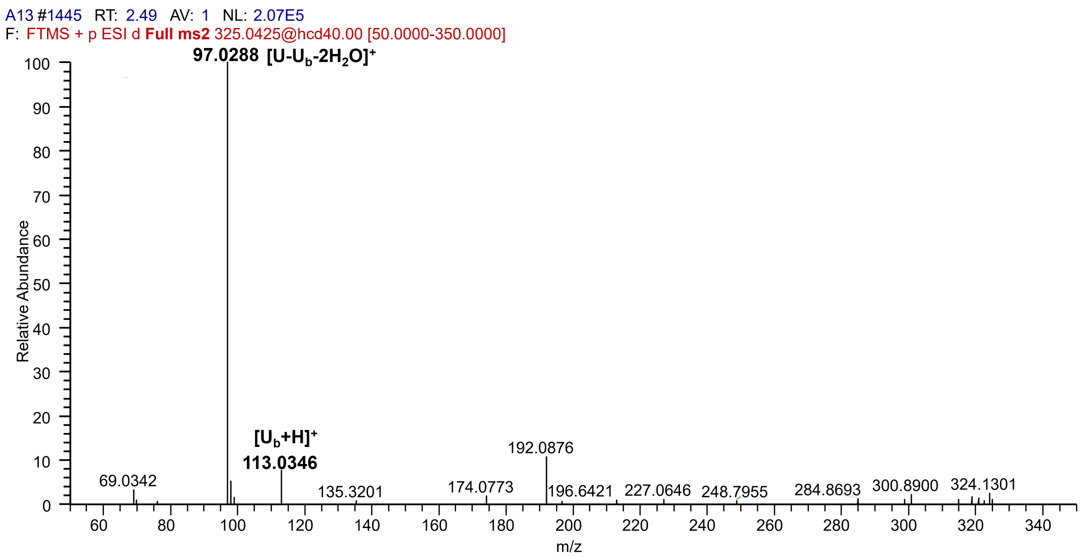

Supplementary Figure 61. The MS^2 spectrum of 5'-UMP [M+H]$^+$ ion (*m/z* 325.04) in **Supplementary Figure 60**. U: Uridine; U$_b$: The base uracil.

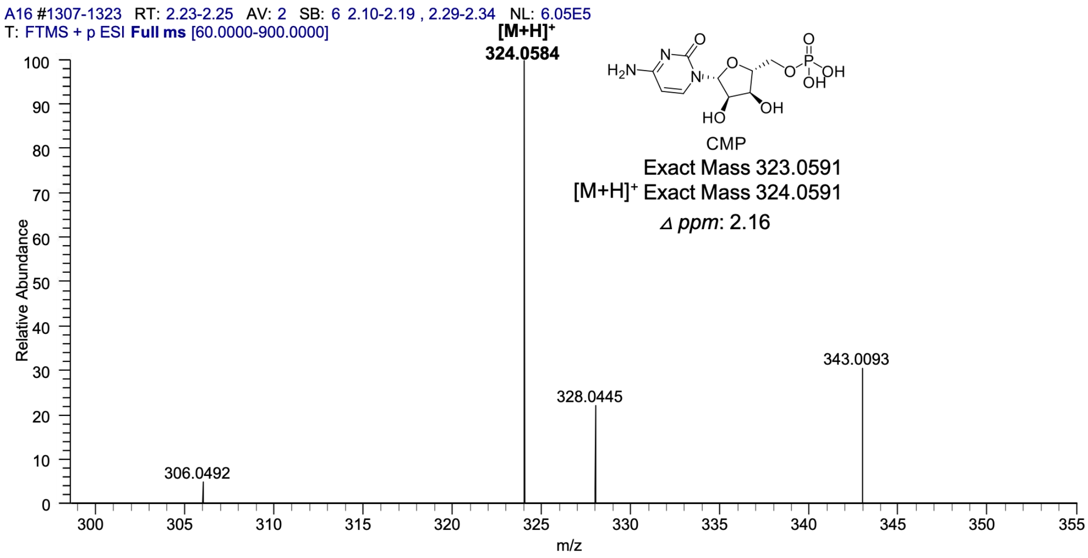

Supplementary Figure 62. The MS spectrum of 5'-CMP (*m/z* 324.06) product formed from the reaction system containing Arg, nucleosides (C and G) and P$_3$m without minerals under the CSS radiation with a total dose of 37.75 mGy in C3 Unit.

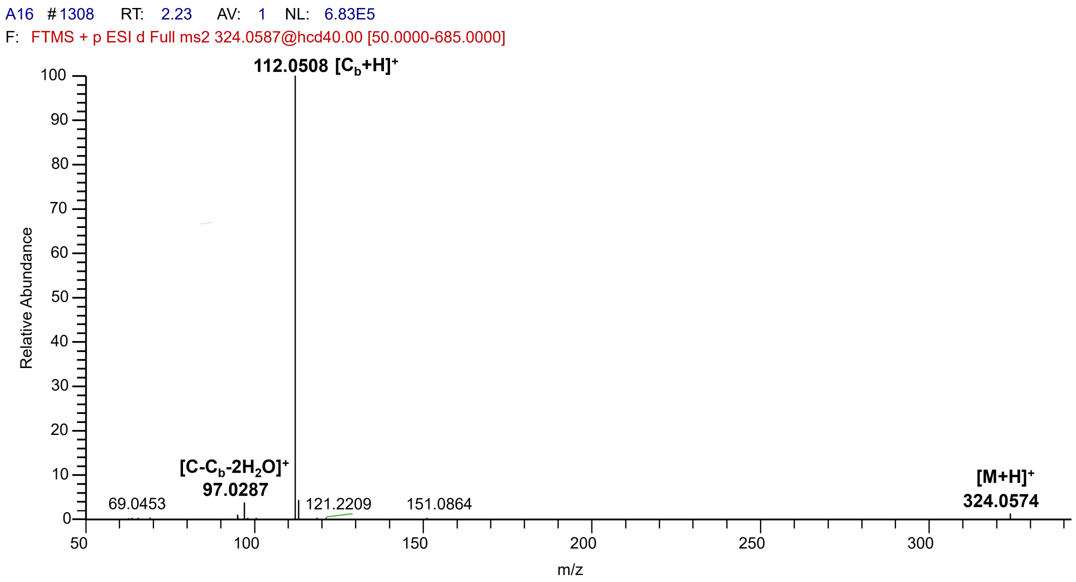

Supplementary Figure 63. The MS^2 spectrum of 5'-CMP $[M+H]^+$ ion (*m/z* 324.06) in **Supplementary Figure 62**. C: Cytidine; C_b: The base cytosine.

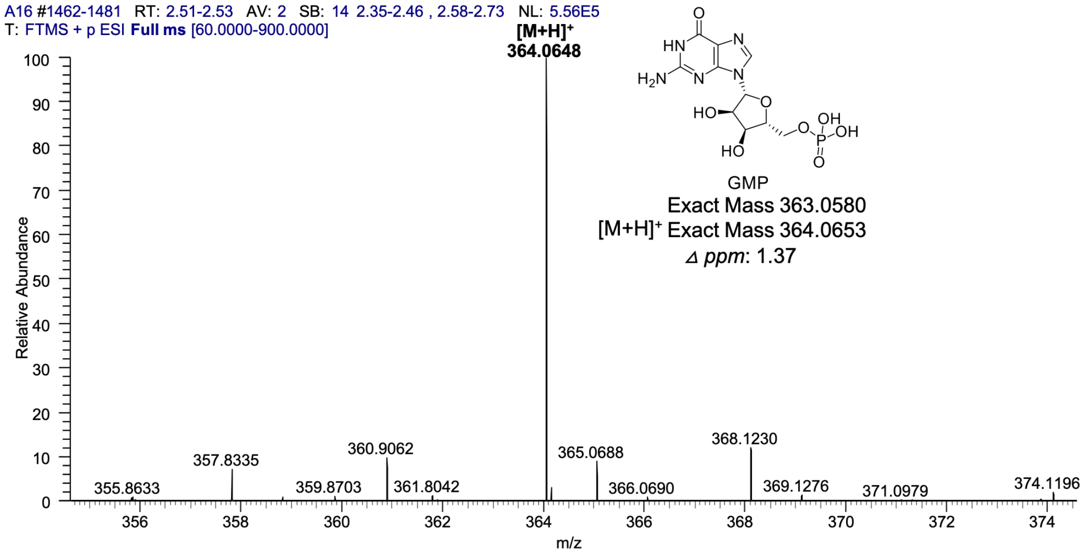

Supplementary Figure 64. The MS spectrum of 5'-GMP (*m/z* 364.06) product formed from the reaction system containing Arg, nucleosides (C and G) and P_3m without minerals under the CSS radiation with a total dose of 37.75 mGy in C3 Unit.

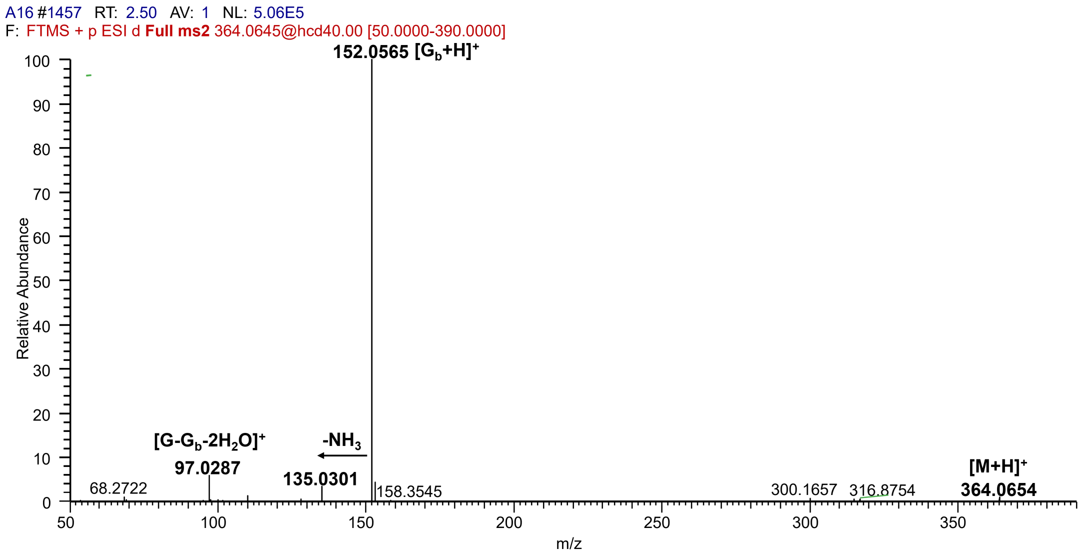

Supplementary Figure 65. The MS^2 spectrum of 5'-GMP ion (*m/z* 364.06) in **Supplementary Figure 64**. G: Guanosine; G_b: The base guanine.

10. MS analysis of the dipeptide and NMP product formed in the reaction system containing the mixed amino acid, nucleosides and P_3m after space radiation exposure in B3 Unit of TZ 6

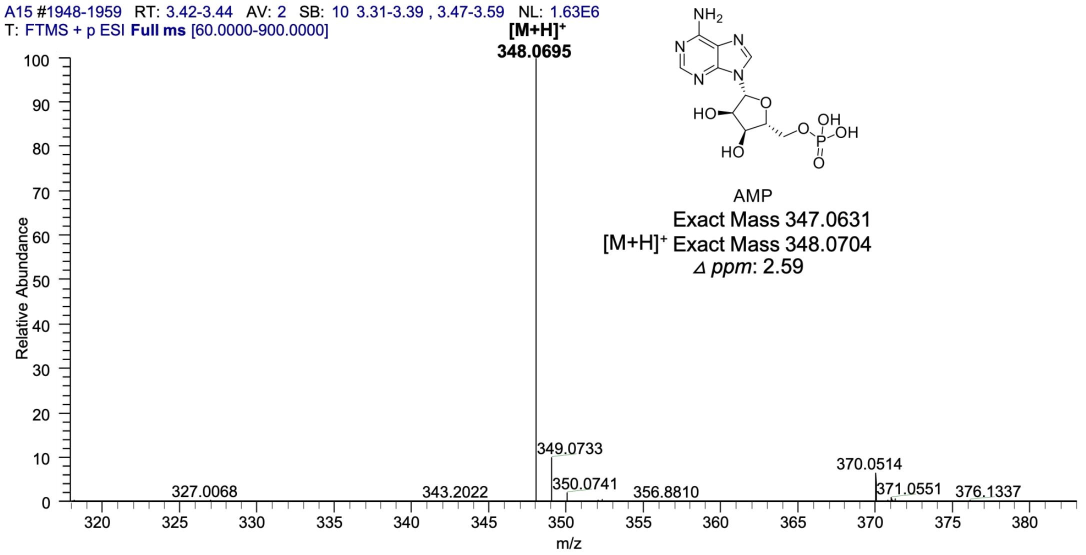

Supplementary Figure 66. The MS spectrum of 5'-AMP (*m/z* 348.07) product formed from the reaction system containing Phe, nucleosides (A and U) and P_3m without minerals under the CSS radiation with a total dose of 101.08 mGy in B3 Unit.

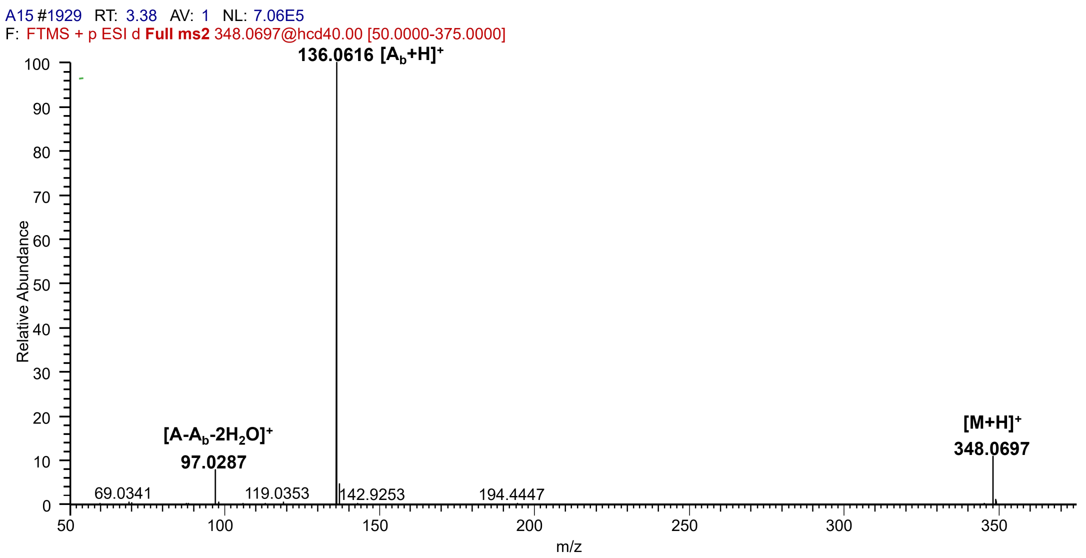

Supplementary Figure 67. The MS^2 spectrum of 5'-AMP $[M+H]^+$ ion (*m/z* 348.07) in **Supplementary Figure 66**. A: Adenosine; A_b: The base adenine.

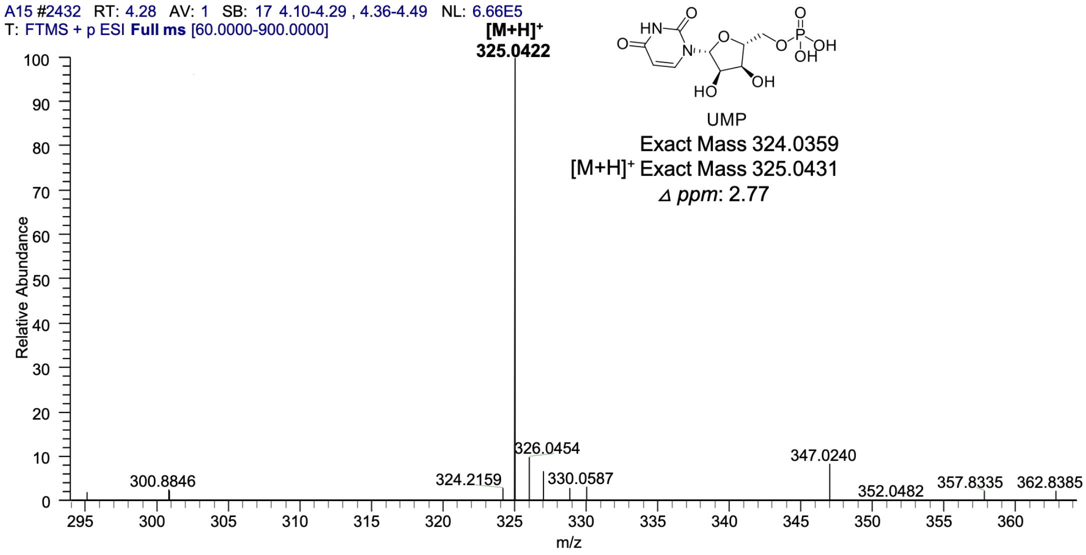

Supplementary Figure 68. The MS spectrum of 5'-UMP (*m/z* 325.04) product formed from the reaction system containing Phe, nucleosides (A and U) and P_3m without minerals under the CSS radiation with a total dose of 101.08 mGy in B3 Unit.

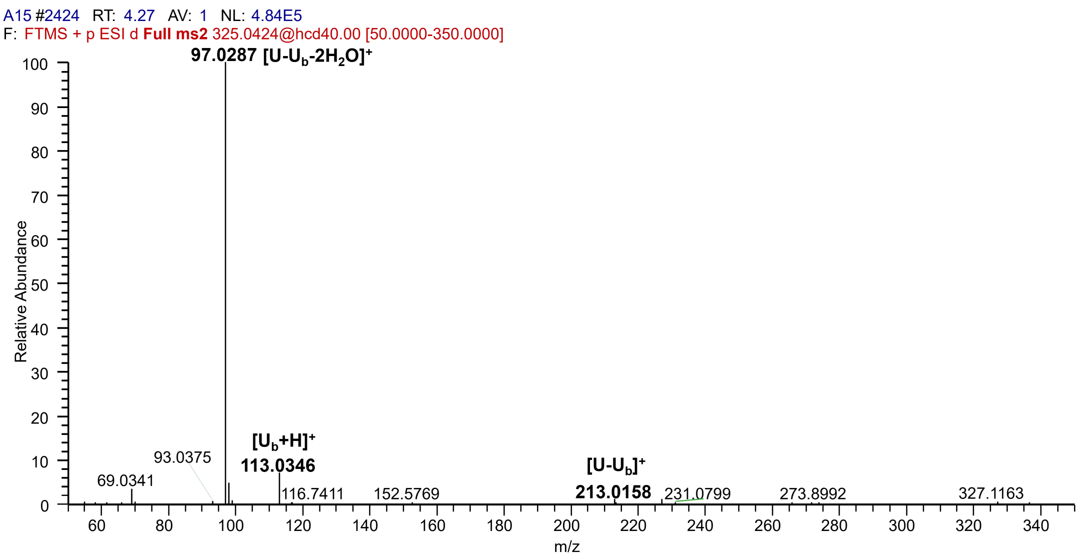

Supplementary Figure 69. The MS^2 spectrum of 5'-UMP ion (*m/z* 325.04) in **Supplementary Figure 68**. U: Uridine; U_b: The base uracil.

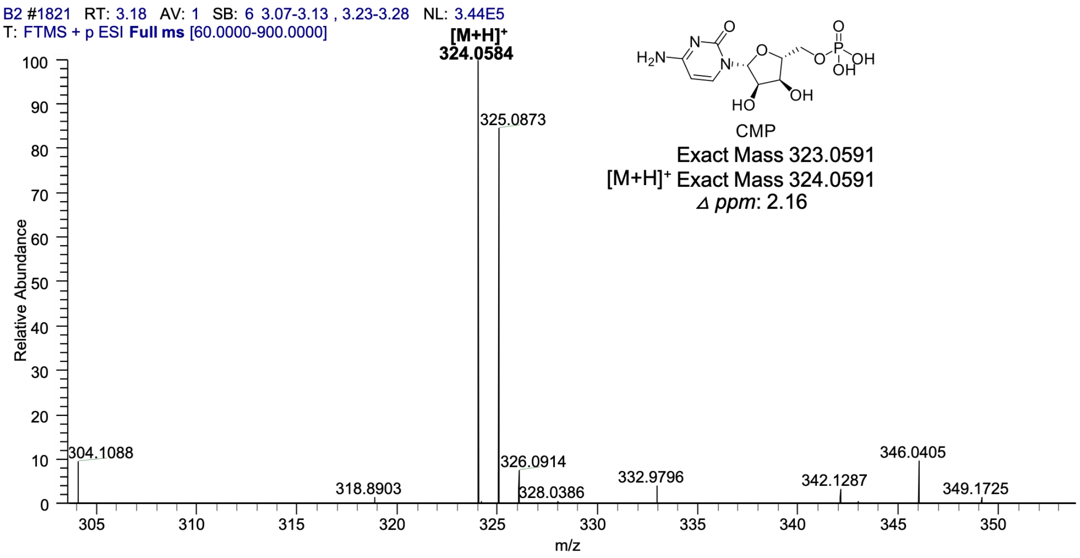

Supplementary Figure 70. The MS spectrum of 5'-CMP (*m/z* 324.05) product formed from the reaction system containing Phe, nucleosides (C and G) and P_3m without minerals under the CSS radiation with a total dose of 101.08 mGy in B3 Unit.

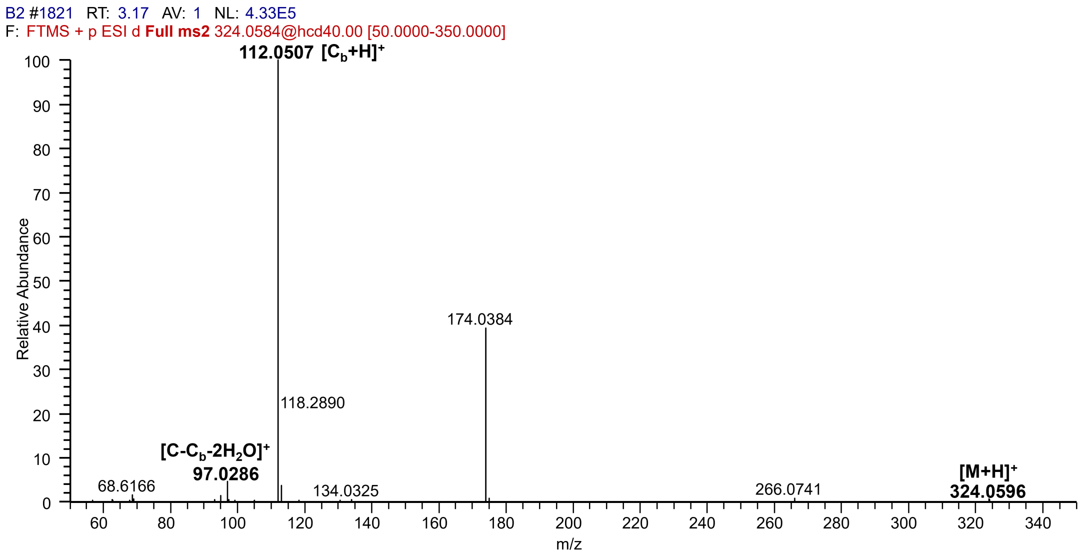

Supplementary Figure 71. The MS^2 spectrum of 5'-CMP $[M+H]^+$ ion (*m/z* 324.06) in **Supplementary Figure 70**. C: Cytidine; C_b: The base cytosine.

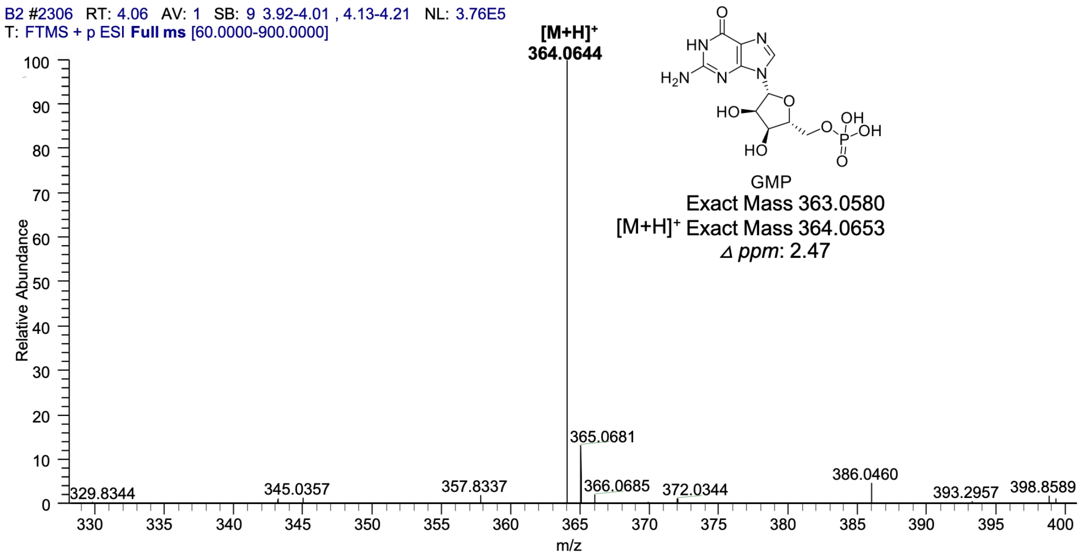

Supplementary Figure 72. The MS spectrum of 5'-GMP (*m/z* 364.06) product formed from the reaction system containing Phe, nucleosides (C and G) and P_3m without minerals under the CSS radiation with a total dose of 101.08 mGy in B3 Unit.

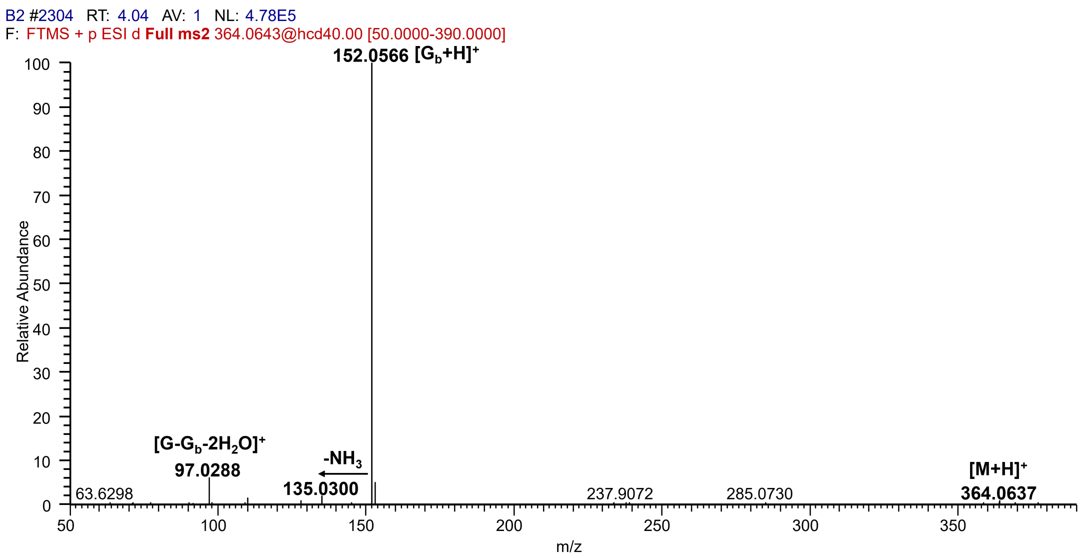

Supplementary Figure 73. The MS^2 spectrum of 5'-GMP $[M+H]^+$ ion (*m/z* 364.06) in **Supplementary Figure 72**. G: Guanosine; G_b: The base guanine.

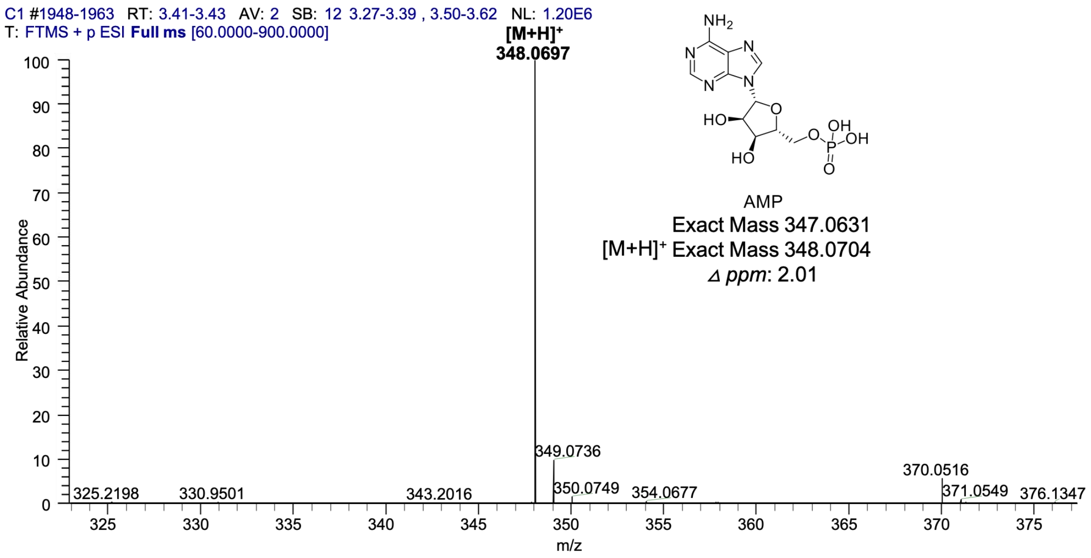

Supplementary Figure 74. The MS spectrum of 5'-AMP (*m/z* 348.07) product formed from the reaction system containing Ala, nucleosides (A and U) and P3m without minerals under the CSS radiation with a total dose of 101.08 mGy in B3 Unit.

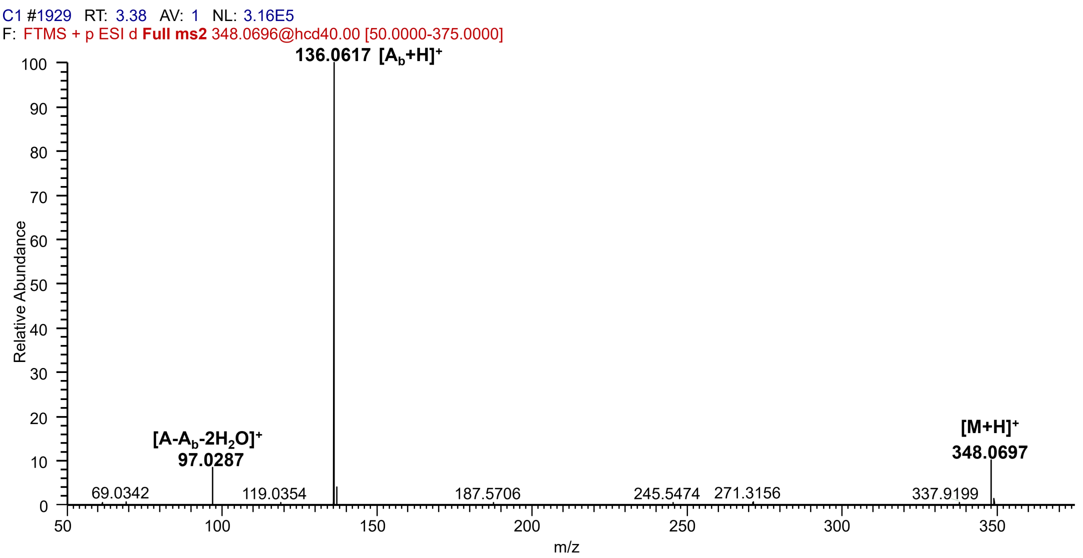

Supplementary Figure 75. The MS^2 spectrum of 5'-AMP $[M+H]^+$ ion (*m/z* 348.07) in **Supplementary Figure 74**. A: Adenosine; A_b: The base adenine.

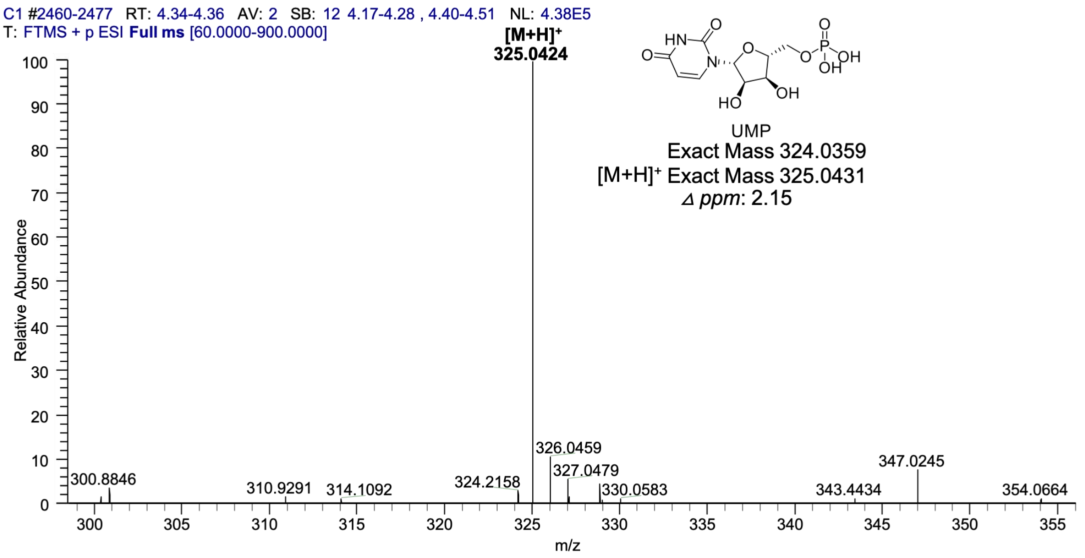

Supplementary Figure 76. The MS spectrum of 5'-UMP (*m/z* 325.04) product formed from the reaction system containing Ala, nucleosides (A and U) and P_3m without minerals under the CSS radiation with a total dose of 101.08 mGy in B3 Unit.

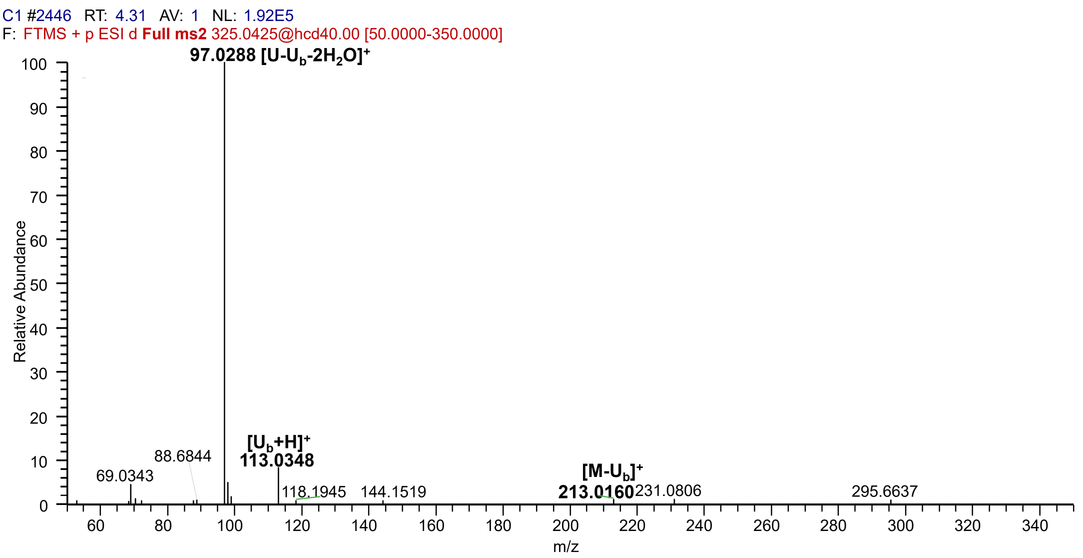

Supplementary Figure 77. The MS^2 spectrum of 5'-UMP $[M+H]^+$ ion (*m/z* 325.04) in **Supplementary Figure 76**. U: Uridine; U_b: The base uracil.

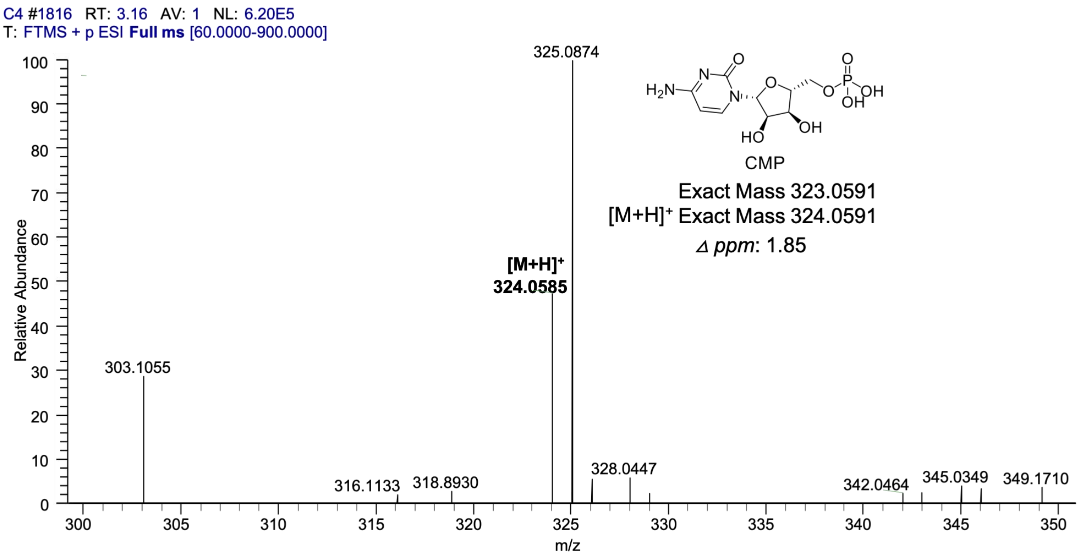

Supplementary Figure 78. The MS spectrum of 5'-CMP (*m/z* 324.06) product formed from the reaction system containing Ala, nucleosides (C and G) and P_3m without minerals under the CSS radiation with a total dose of 101.08 mGy in B3 Unit.

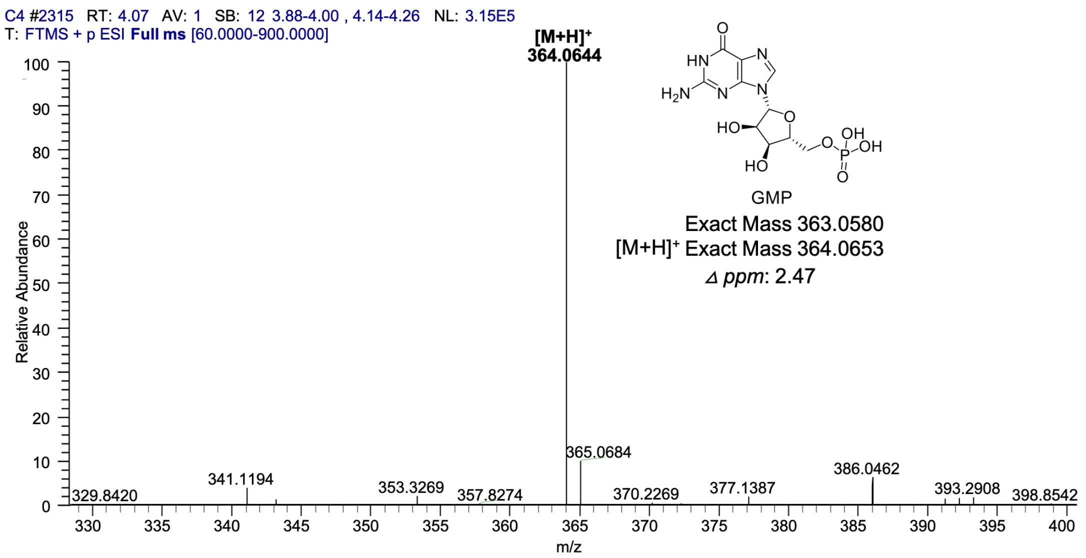

Supplementary Figure 79. The MS spectrum of 5'-GMP (*m/z* 364.06) product formed from the reaction system containing Ala, nucleosides (C and G) and P_3m without minerals under the CSS radiation with a total dose of 101.08 mGy in B3 Unit.

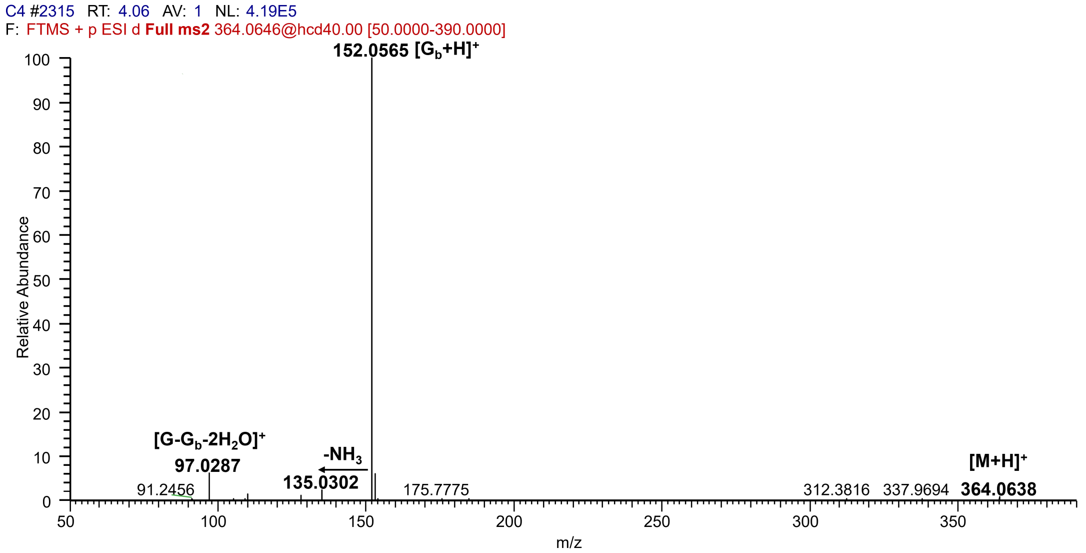

Supplementary Figure 80. The MS^2 spectrum of 5'-GMP $[M+H]^+$ ion (*m/z* 364.06) in **Supplementary Figure 79**. G: Guanosine; G_b: The base guanine.

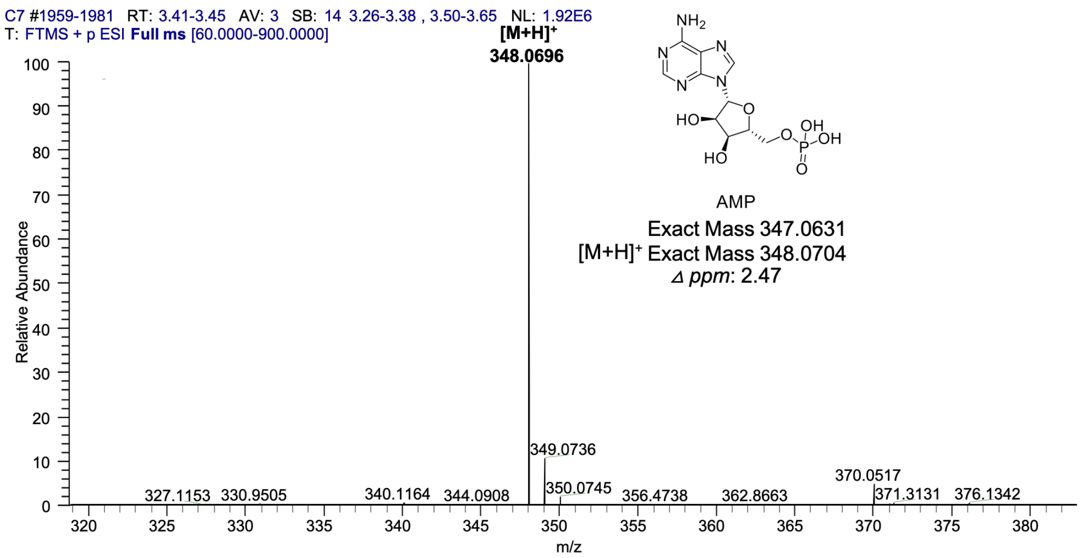

Supplementary Figure 81. The MS spectrum of 5'-AMP (*m/z* 348.07) product formed from the reaction system containing Arg, nucleosides (A and U) and P_3m without minerals under the CSS radiation with a total dose of 101.08 mGy in B3 Unit.

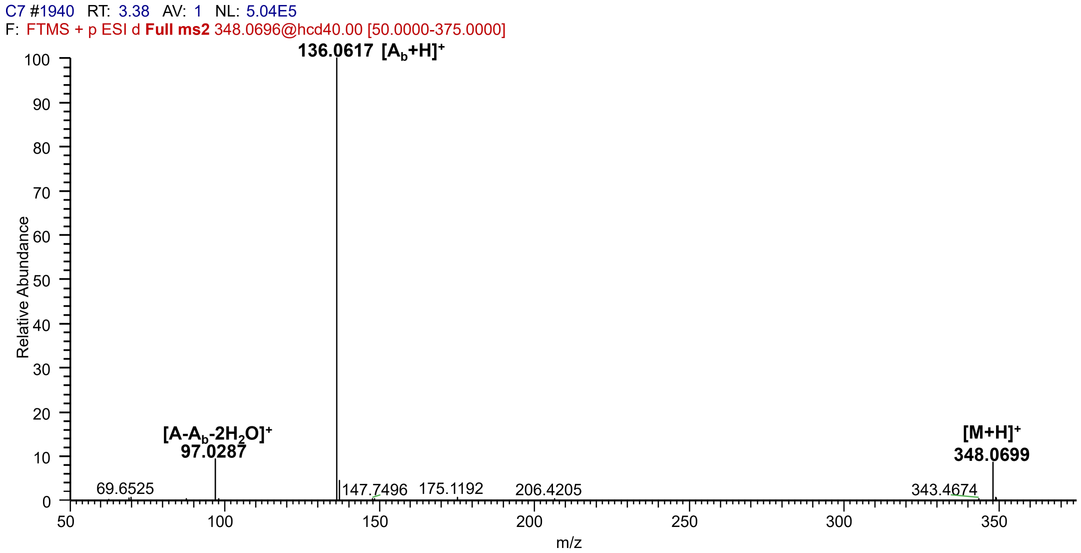

Supplementary Figure 82. The MS^2 spectrum of 5'-AMP $[M+H]^+$ ion (*m/z* 348.07) in **Supplementary Figure 81**. A: Adenosine; A_b: The base adenine.

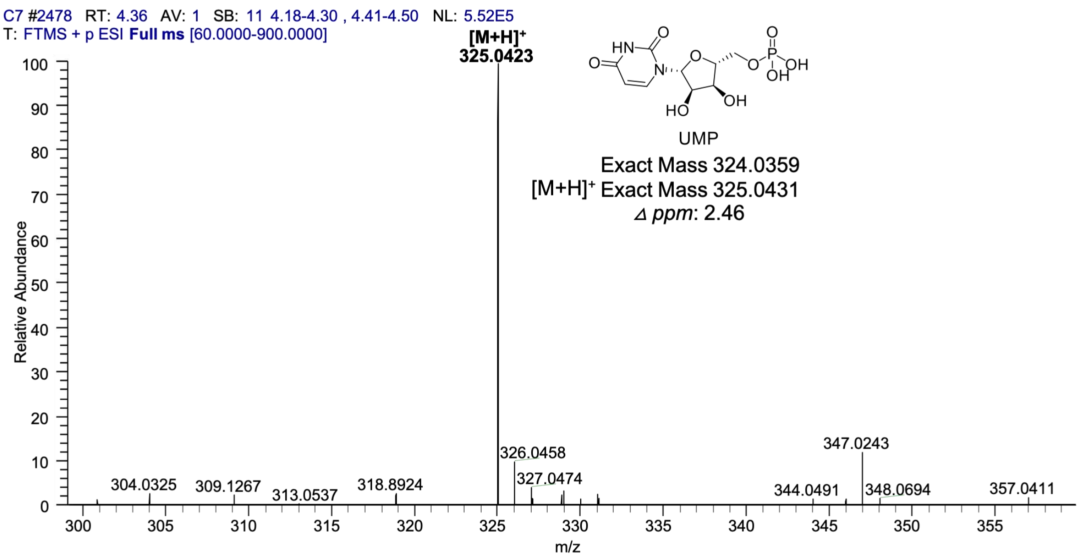

Supplementary Figure 83. The MS spectrum of 5'-UMP (*m/z* 325.04) product formed from the reaction system containing Arg, nucleosides (A and U) and P_3m without minerals under the CSS radiation with a total dose of 101.08 mGy in B3 Unit.

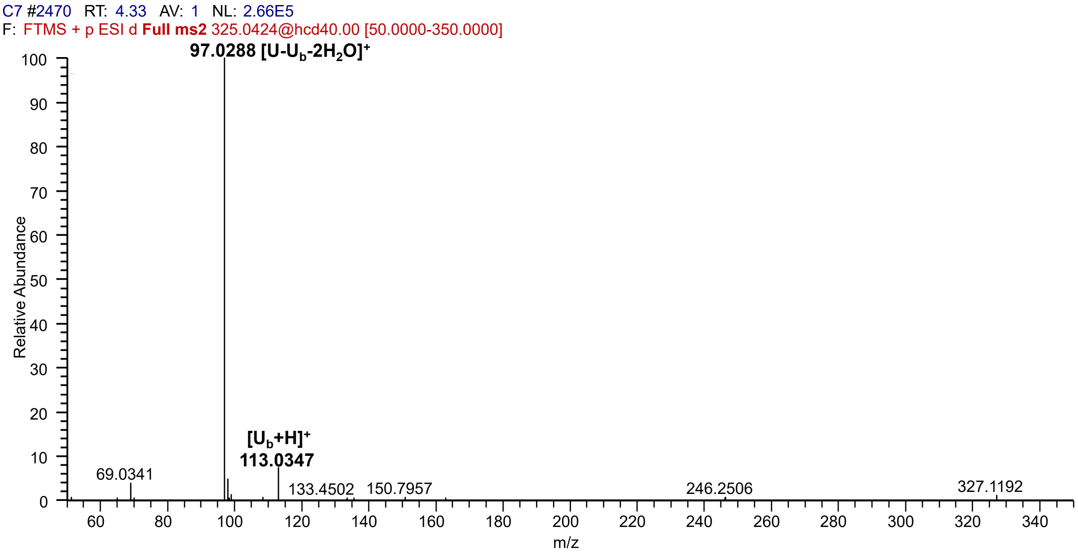

Supplementary Figure 84. The MS^2 spectrum of 5'-UMP $[M+H]^+$ ion (*m/z* 325.04) in **Supplementary Figure 83**. U: Uridine; U_b: The base uracil.

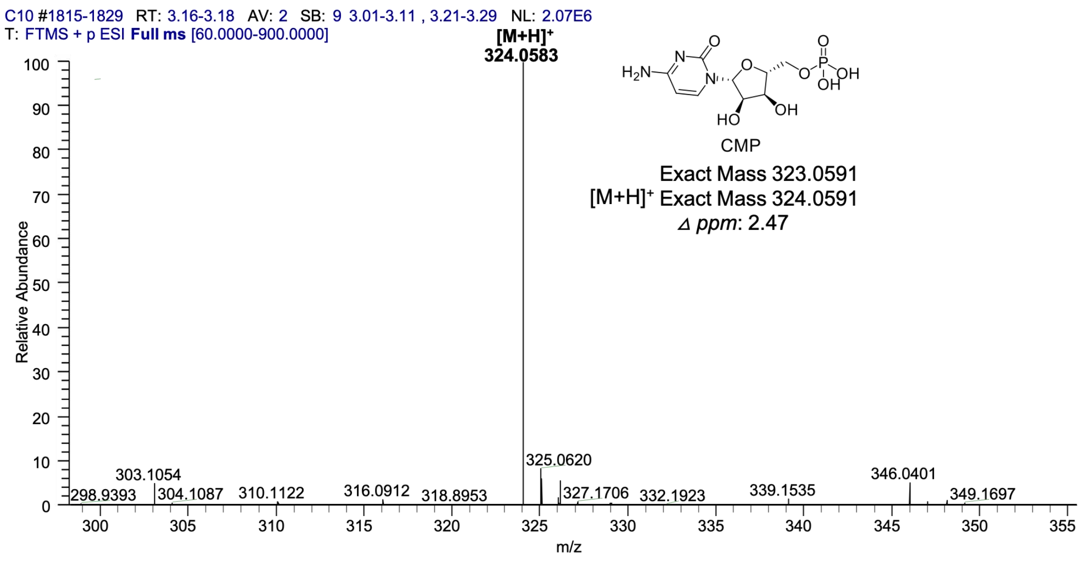

Supplementary Figure 85. The MS spectrum of 5'-CMP (*m/z* 324.06) product formed from the reaction system containing Arg, nucleosides (C and G) and P_3m without minerals under the CSS radiation with a total dose of 101.08 mGy in B3 Unit.

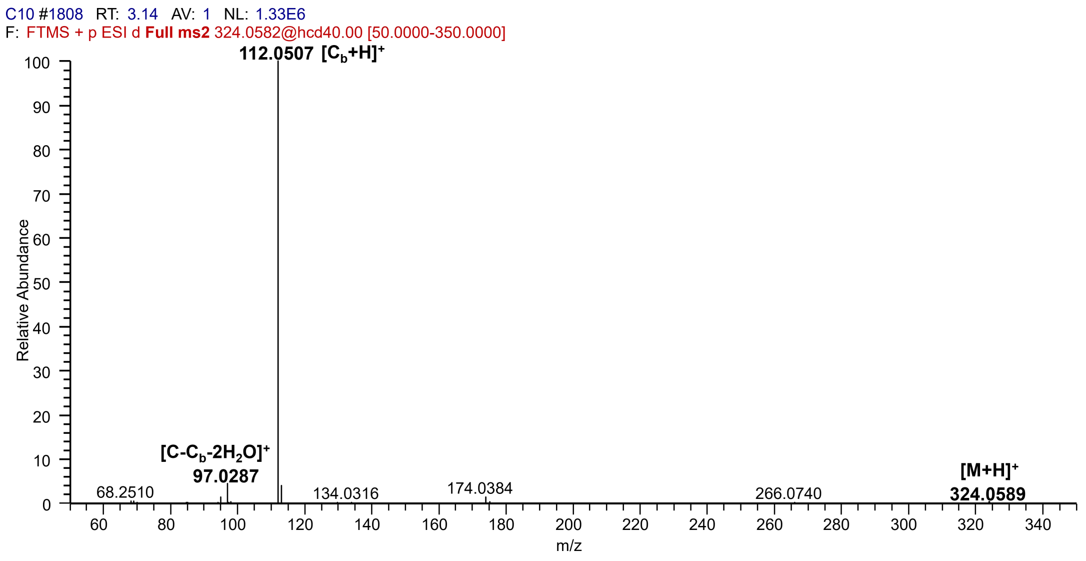

Supplementary Figure 86. The MS^2 spectrum of 5'-CMP $[M+H]^+$ ion (*m/z* 324.06) in **Supplementary Figure 85**. C: Cytidine; C_b: The base cytosine.

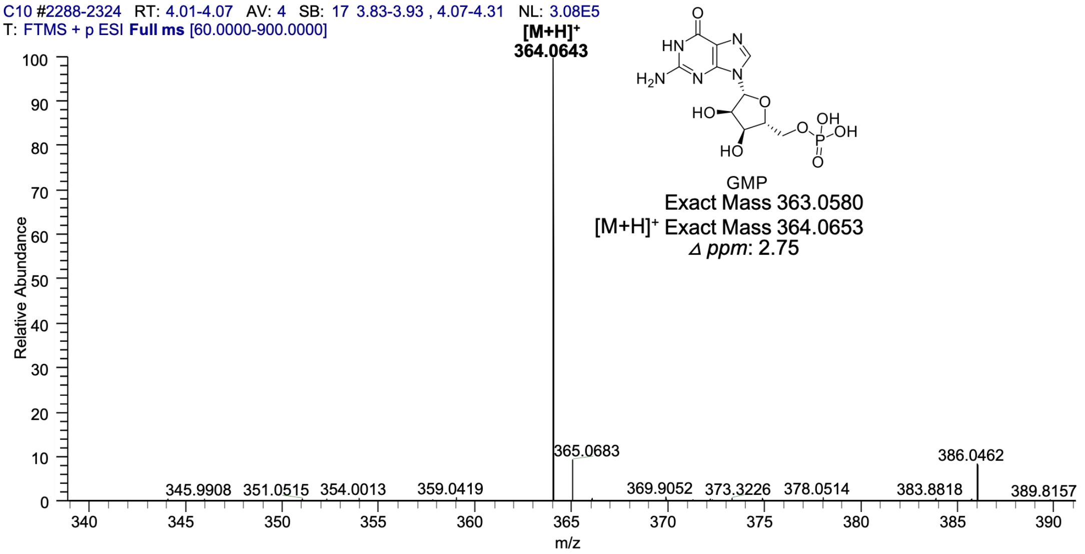

Supplementary Figure 87. The MS spectrum of 5'-GMP (*m/z* 364.06) product formed from the reaction system containing Arg, nucleosides (C and G) and P_3m without minerals under the CSS radiation with a total dose of 101.08 mGy in B3 Unit.

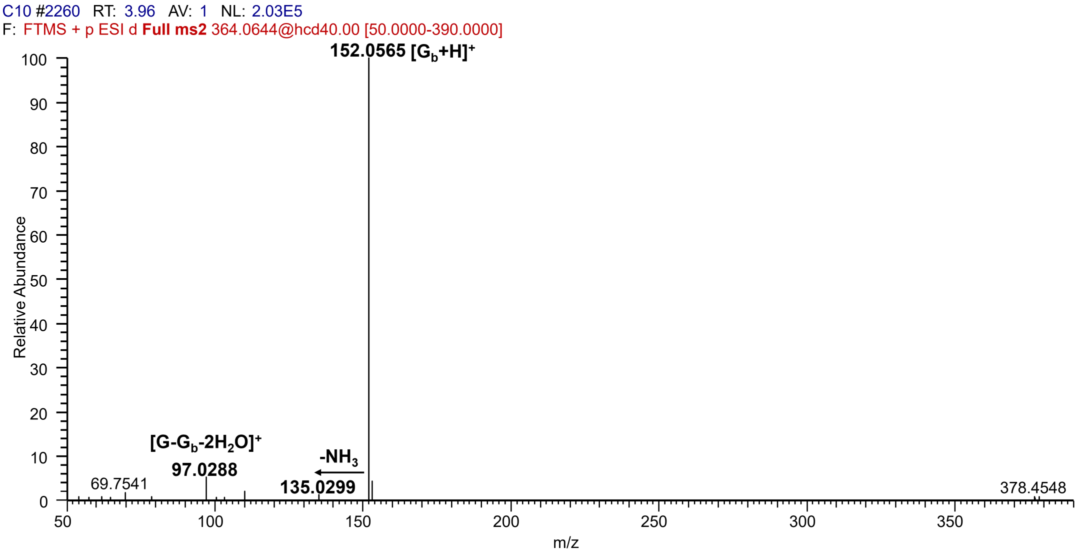

Supplementary Figure 88. The MS^2 spectrum of 5'-GMP $[M+H]^+$ ion (*m/z* 364.06) in **Supplementary Figure 87**. G: Guanosine; G_b: The base guanine.

11. MS analysis of the products formed in the reaction system containing Phe, nucleosides (A and U) and P3m under the ground radiation conditions

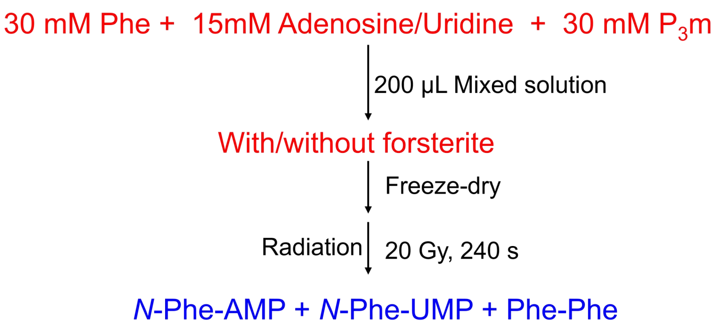

30 mmol/L P3m and 30 mmol/L Phe solutions were well-mixed with 30 mmol/L nucleosides solution (15 mmolL Adenosine and 15 mmol/L Uridine) in a 1.5 mL EP tube. To aliquot 200 μL of the aforementioned mixed solution into sample containers. Among the above two sample containers, the container containing forsterite was selected as the experimental group, while another container without forsterite was used as the blank control group. Subsequently, all the samples were freeze-dried by a freeze-dryer. The irradiation dose of 20 Gy was achieved using exposure factors of 160 kV, 25 mA, and 240 s in the absence of a 0.3 mm copper filter, employing RS2000 X-rays biological irradiator.

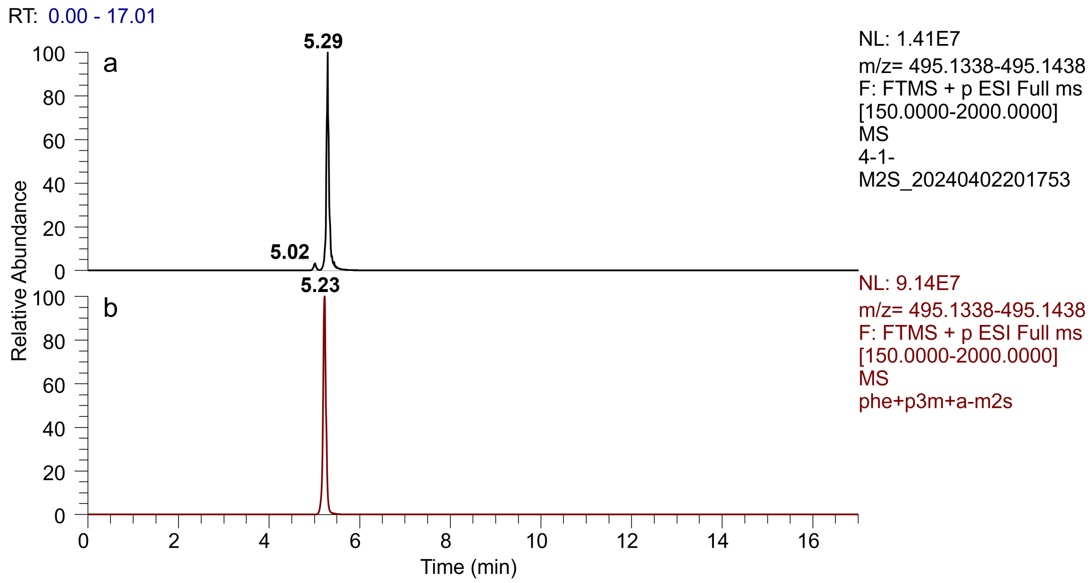

Supplementary Figure 89. a. The EIC of the product *N*-Phe-AMP (*m/z* 495.1388) in the presence of forsterite. b. The EIC of the parallel sample which was stored in a 4 °C refrigerator for six months. The results indicate that 2'-*N*-Phe-AMP exhibited instability and was susceptible to hydrolysis, resulting in the survival of only 5'-*N*-Phe-AMP after storing for six months in a 4 °C refrigerator.

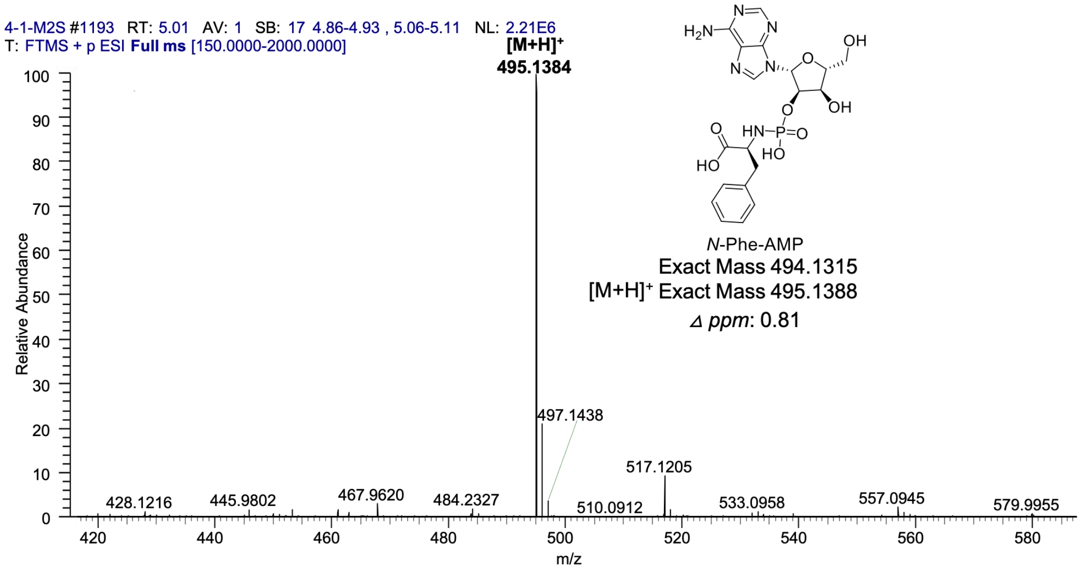

Supplementary Figure 90. The MS spectrum of the product 2'-*N*-Phe-AMP (*m/z* 495.1384, corresponding to the peak RT=5.02 min, 2'-*N*-Phe-AMP, in **Supplementary Figure 89**).

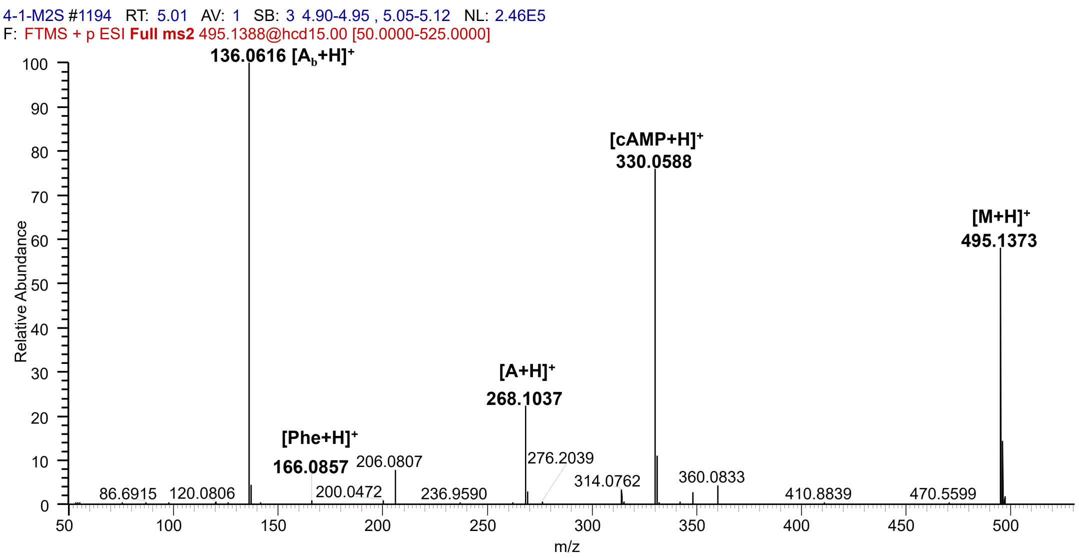

Supplementary Figure 91. The MS^2 spectrum of 2'-*N*-Phe-AMP $[M+H]^+$ ion (*m/z* 495.1373) in **Supplementary Figure 90**. A: Adenosine; A_b: The base adenine.

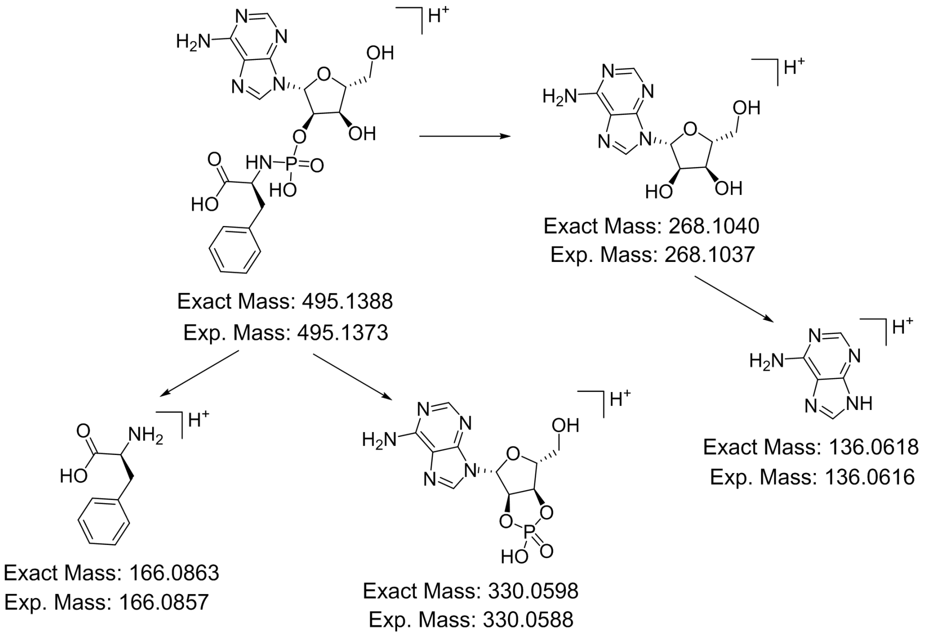

Supplementary Figure 92. The possible MS^2 fragmentation pathways of the product 2'-*N*-Phe-AMP in **Supplementary Figure 91** (*m/z* 495.1373).

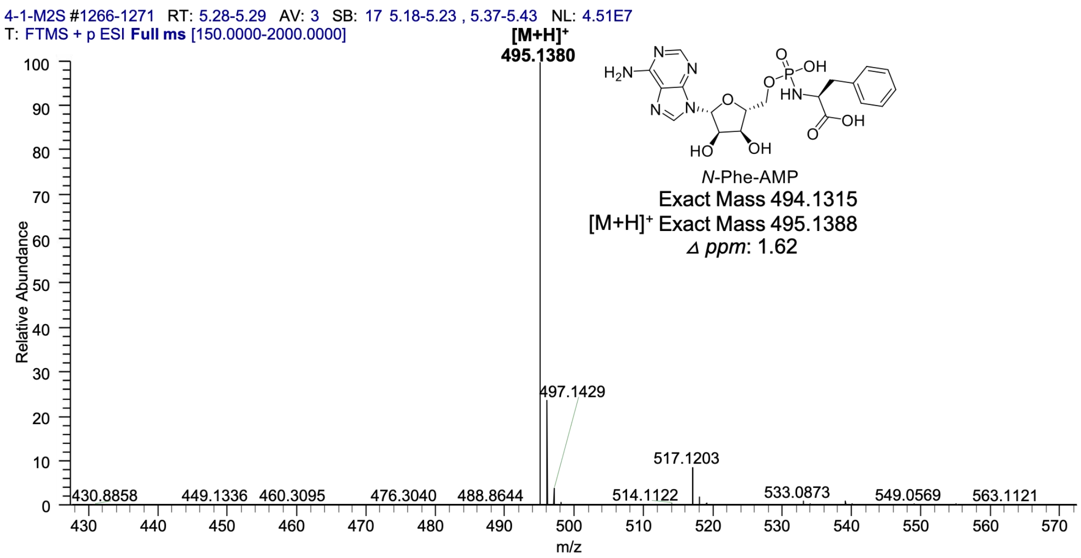

Supplementary Figure 93. The MS spectrum of the product 5'-*N*-Phe-AMP (*m/z* 495.1380, corresponding to the peak RT=5.29 min, 5'-*N*-Phe-AMP in **Supplementary Figure 89**).

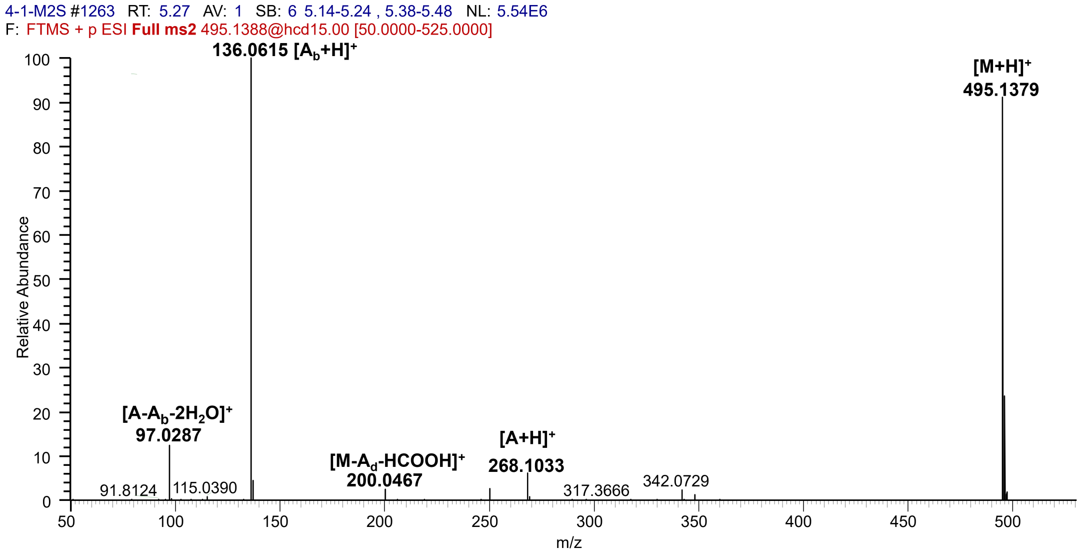

Supplementary Figure 94. The MS^2 spectrum of 5'-*N*-Phe-AMP $[M+H]^+$ ion (*m/z* 495.1379) in **Supplementary Figure 93**. A: Adenosine; A_b: The base adenine; A_d: Adenosine losing a H_2O.

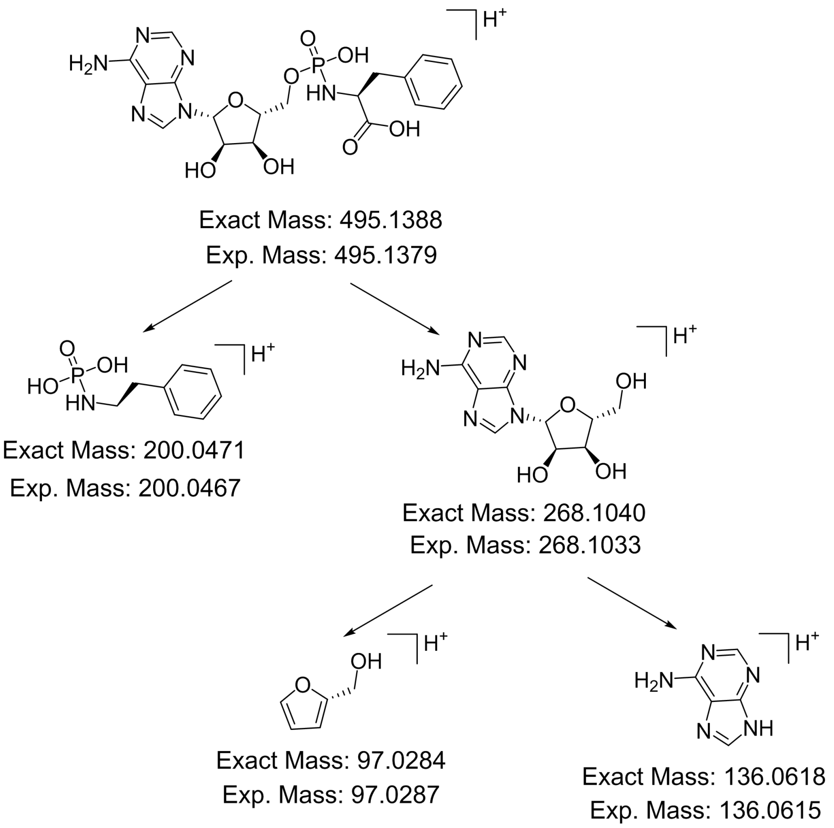

Supplementary Figure 95. The possible MS^2 fragmentation pathways of 5'-*N*-Phe-AMP in **Supplementary Figure 94**.

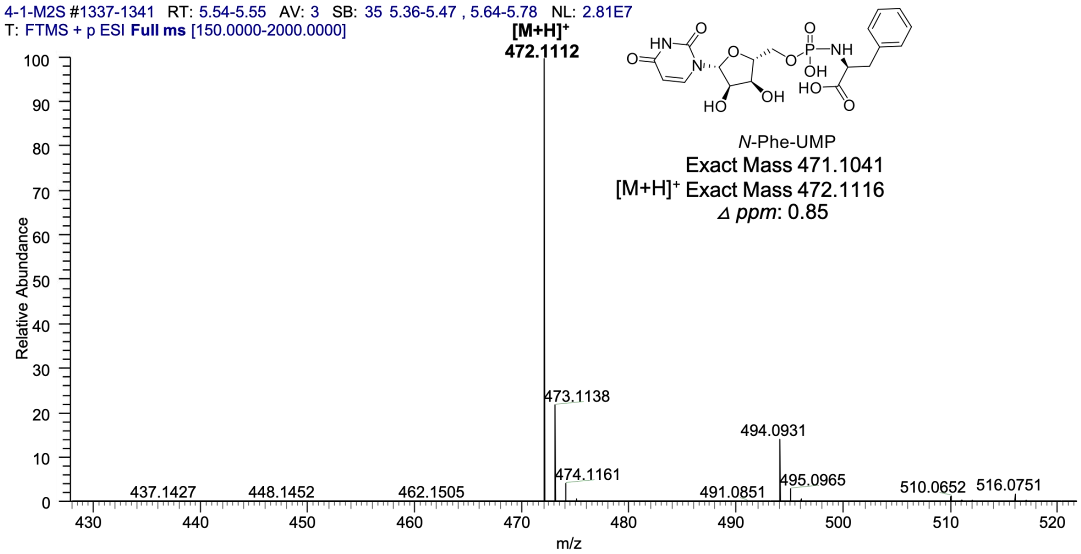

Supplementary Figure 96. The MS spectrum of the product 5'-*N*-Phe-UMP (*m/z* 472.1112) in the presence of forsterite.

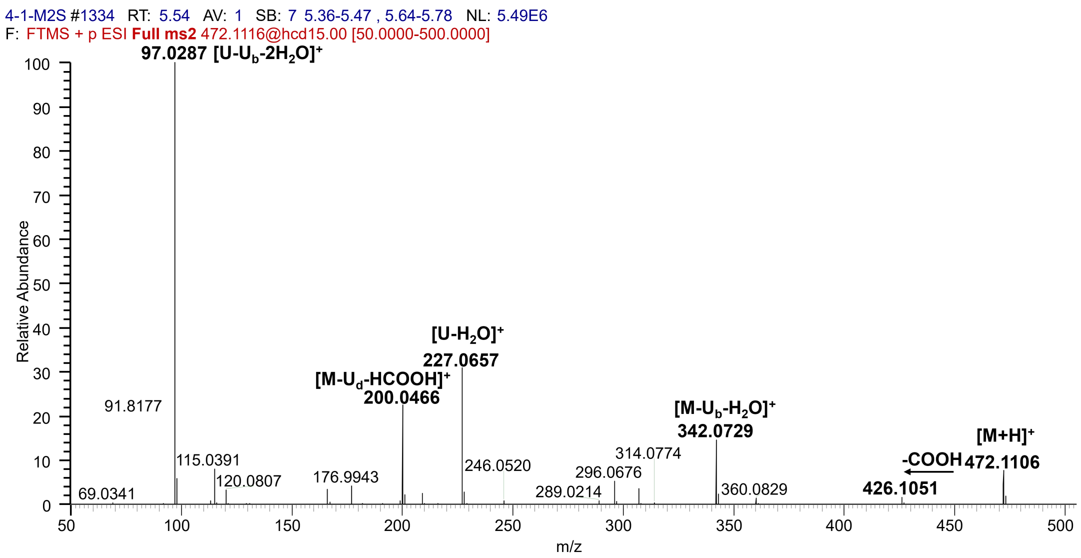

Supplementary Figure 97. The MS^2 spectrum of 5'-*N*-Phe-UMP $[M+H]^+$ ion (*m/z* 472.1106) in **Supplementary Figure 96**. U: Uridine; U_b: The base uracil; U_d: Uridine losing an H_2O.

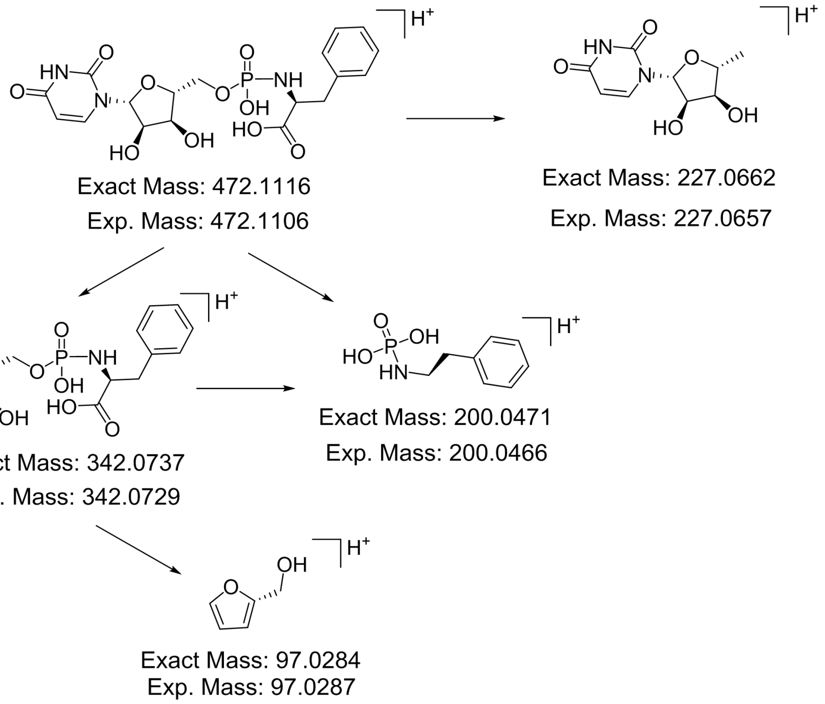

Supplementary Figure 98. The possible MS^2 fragmentation pathways of 5'-*N*-Phe-UMP ion (*m/z* 472.1106) in **Supplementary Figure 97**.

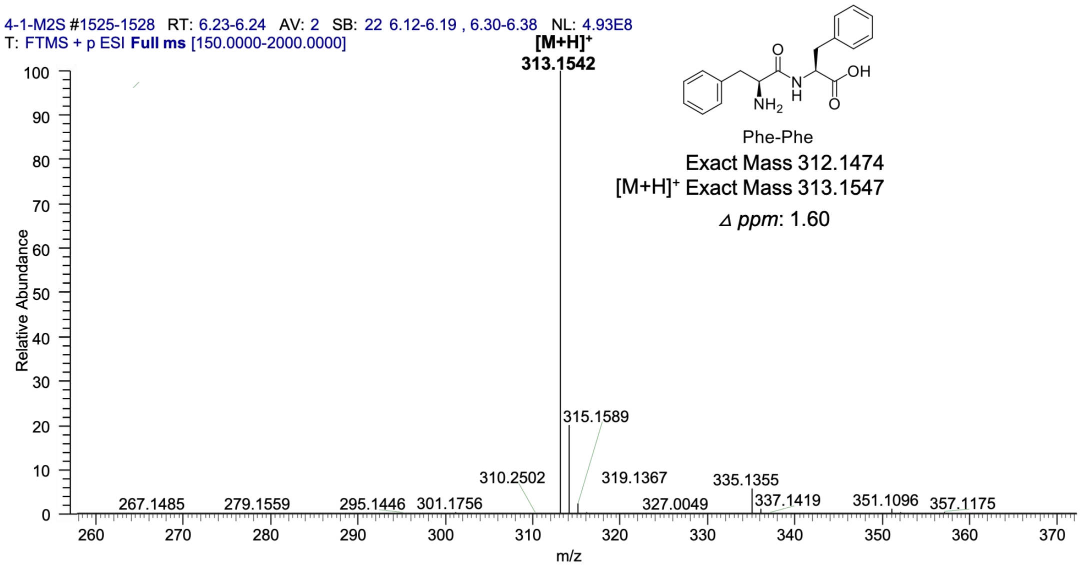

Supplementary Figure 99. The MS spectrum of the product Phe-Phe (*m/z* 313.1542) in the presence of forsterite.

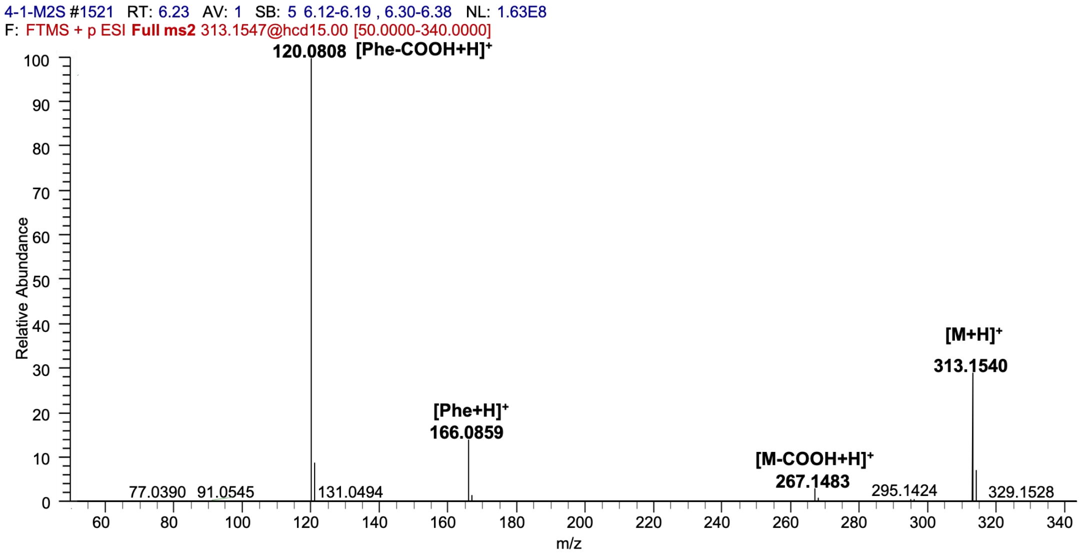

Supplementary Figure 100. The MS^2 spectrum of Phe-Phe $[M+H]^+$ ion (*m/z* 313.1540) in **Supplementary Figure 99**.

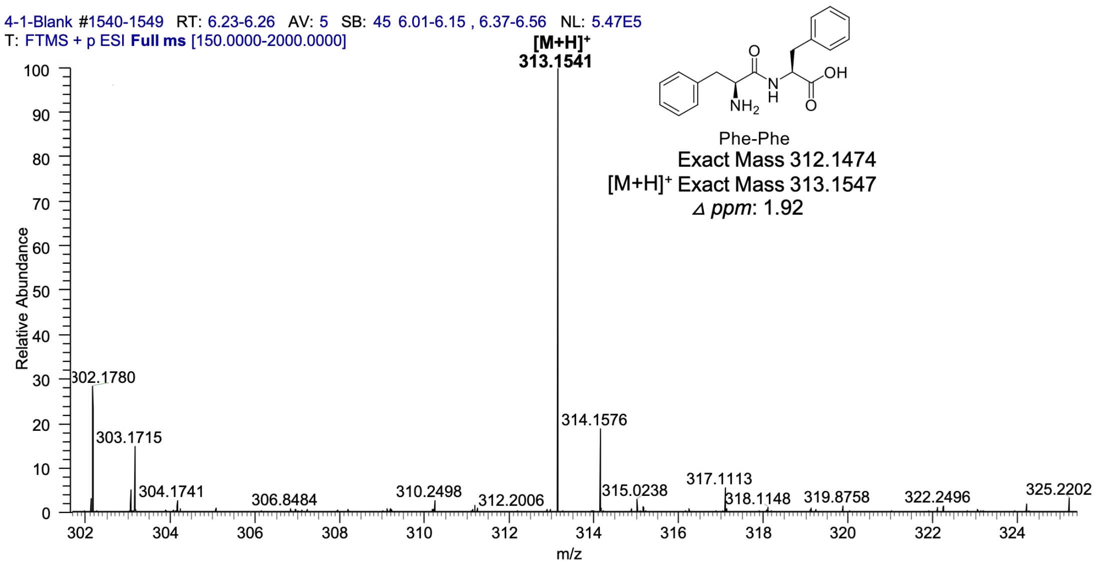

Supplementary Figure 101. The MS spectrum of the product Phe-Phe (*m/z* 313.1541) in the absence of forsterite (Blank control).

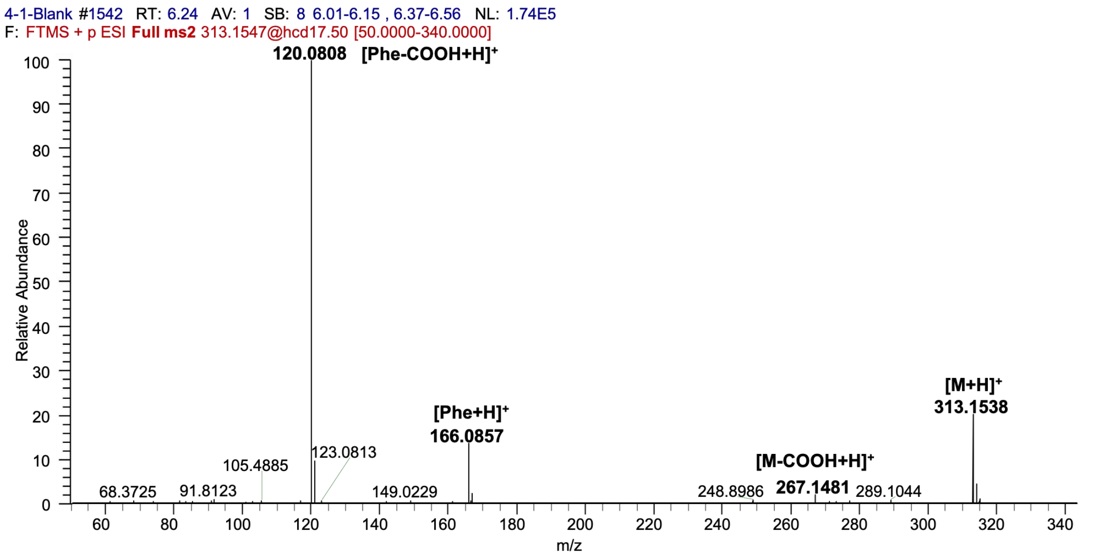

Supplementary Figure 102. The MS^2 spectrum of Phe-Phe $[M+H]^+$ ion (*m/z* 313.1538) in **Supplementary Figure 101**.

12. MS analysis of the products formed in the reaction system containing Phe, nucleosides (C and G), and P$_3$m with forsterite on the ground radiation condition

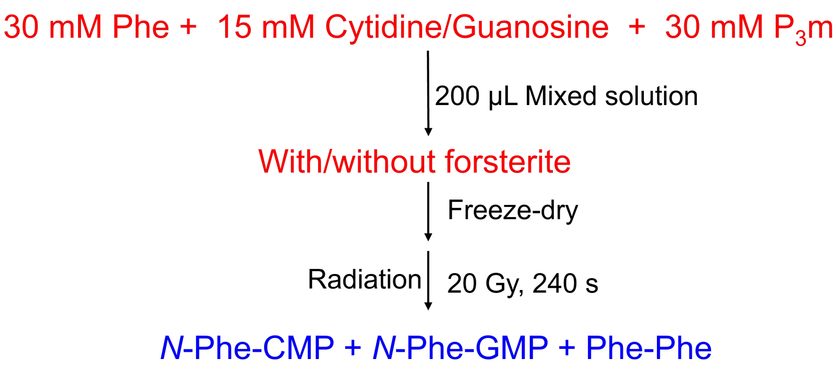

30 mmol P$_3$m and 30 mmol Phe solutions were well-mixed with 30 mmol nucleosides solution (15 mmol Cytidine and 15 mmol Guanosine) in a 1.5 mL EP tube. To aliquot 200 μL of the aforementioned mixed solution into sample containers. Among the above two sample containers, the container containing forsterite was selected as the experimental group, while another container without forsterite was used as the blank control group. Subsequently, all the samples were freeze-dried by a freeze-dryer. The irradiation dose of 20 Gy was achieved using exposure factors of 160 kV, 25 mA, and 240 s without a 0.3 mm copper filter, employing an RS2000 X-rays biological irradiator.

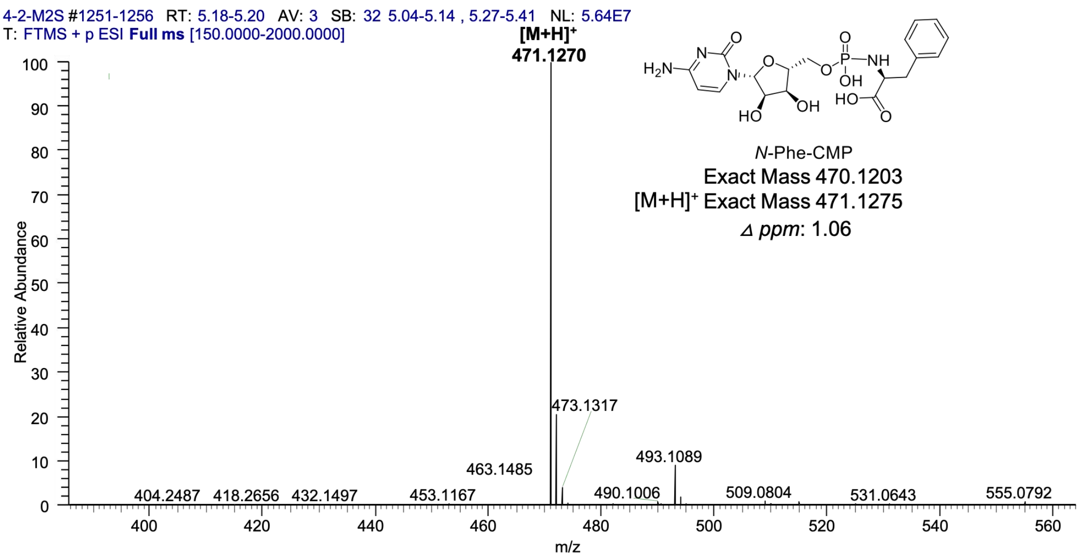

Supplementary Figure 103. The MS spectrum of the product 5'-*N*-Phe-CMP (*m/z* 471.1270) in the presence of forsterite.

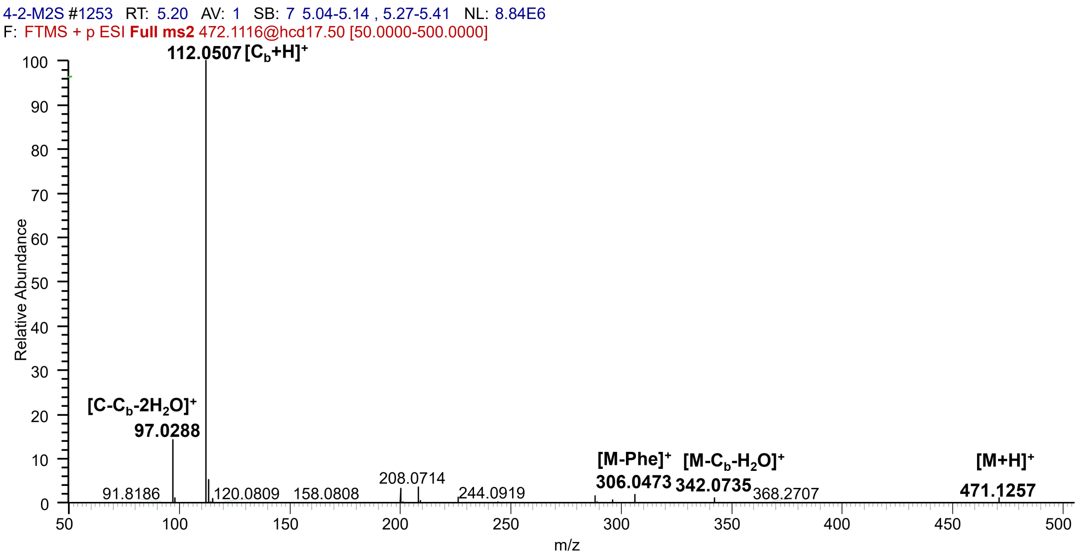

Supplementary Figure 104. The MS^2 spectrum of 5'-*N*-Phe-CMP [M+H]$^+$ ion (*m/z* 471.1257) in **Supplementary Figure 103**. C: Cytidine; C$_b$: The base cytosine.

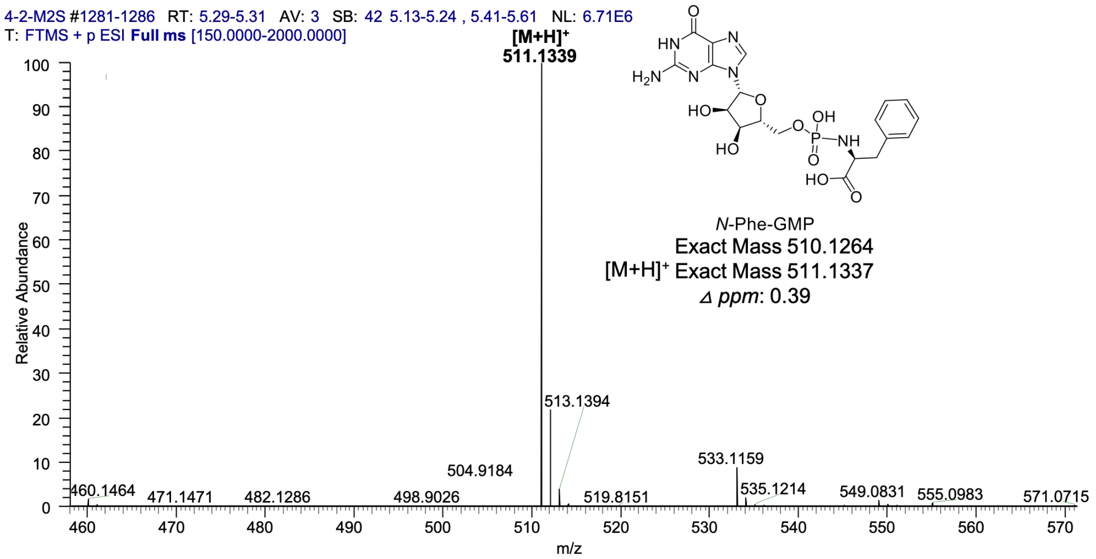

Supplementary Figure 105. The MS spectrum of the product 5'-*N*-Phe-GMP (*m/z* 511.1339) in the presence of forsterite.

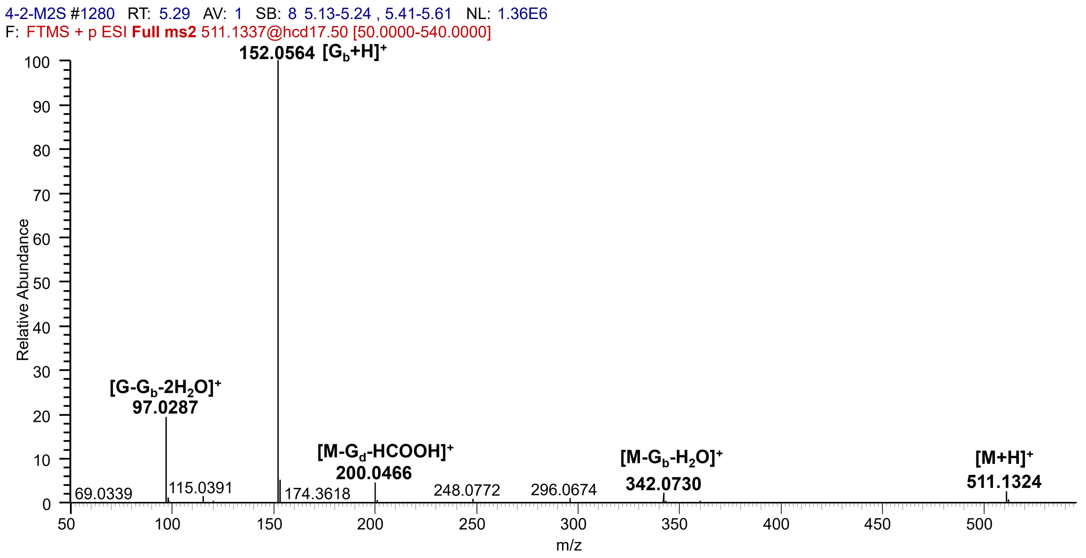

Supplementary Figure 106. The MS^2 spectrum of 5'-*N*-Phe-GMP $[M+H]^+$ ion (*m/z* 511.1324) in **Supplementary Figure 105**. G: Guanosine; G_b: The base guanine; G_d: Guanosine losing an H_2O.

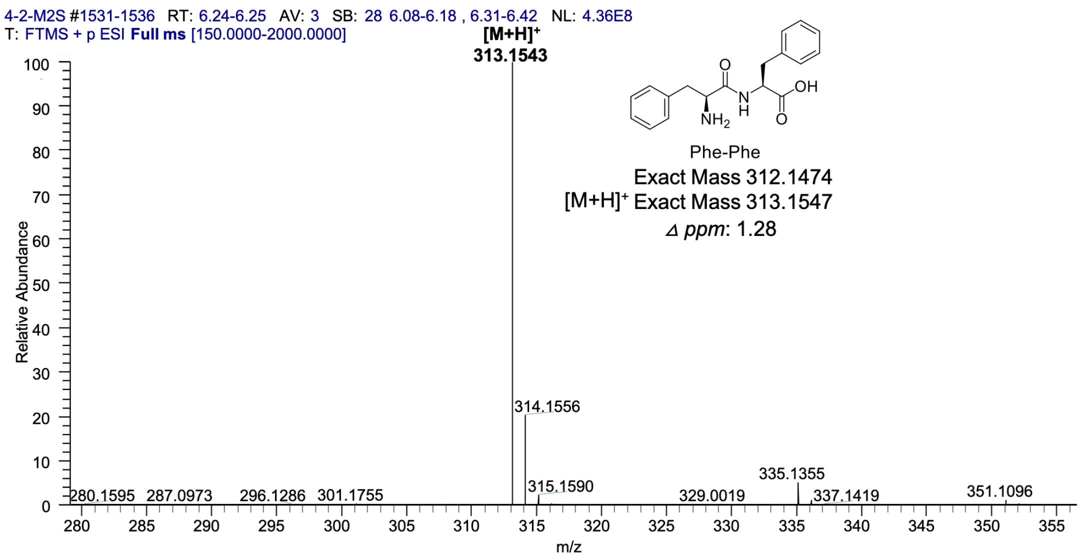

Supplementary Figure 107. The MS spectrum of the product Phe-Phe (*m/z* 313.1543) in the presence of forsterite.

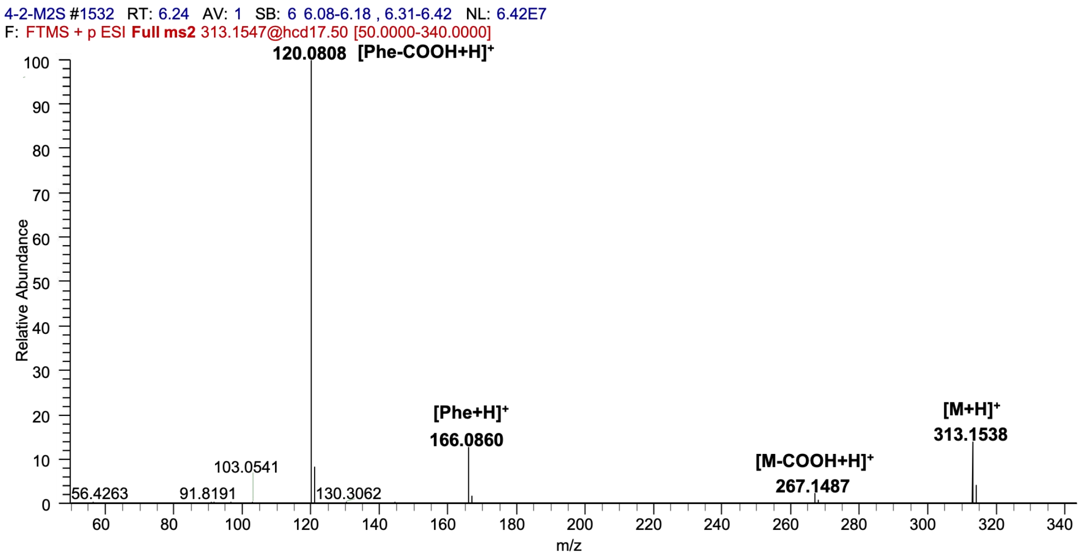

Supplementary Figure 108. The MS^2 spectrum of Phe-Phe $[M+H]^+$ ion (*m/z* 313.1538) in **Supplementary Figure 107**.

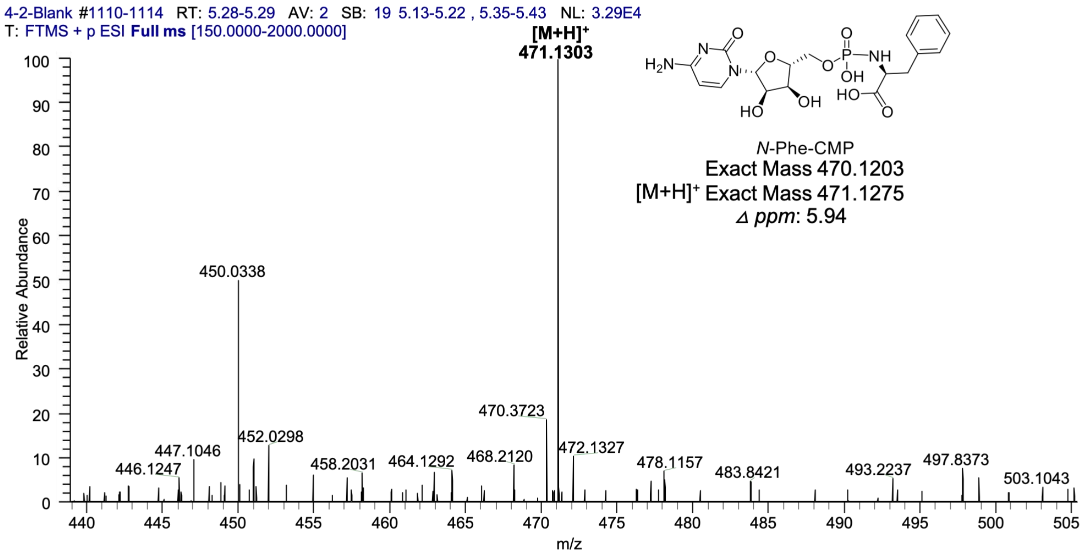

Supplementary Figure 109. The MS spectrum of the product 5’-*N*-Phe-CMP (*m/z* 471.1303) in the absence of forsterite (Blank control).

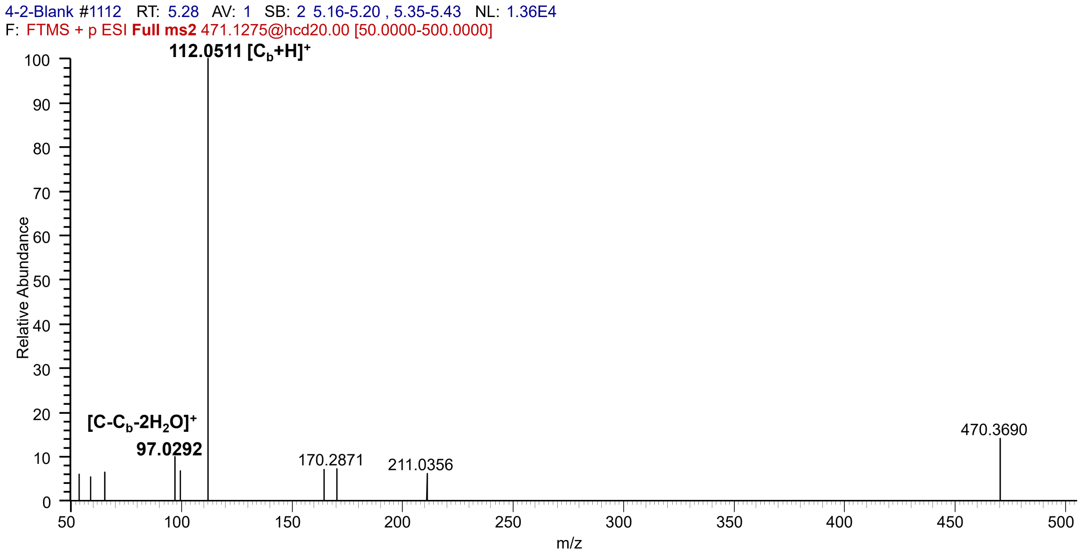

Supplementary Figure 110. The MS^2 spectrum of 5’-*N*-Phe-CMP [M+H]$^+$ ion (*m/z* 471.1275) in **Supplementary Figure 109**. C: Cytidine; C_b: The base cytosine.

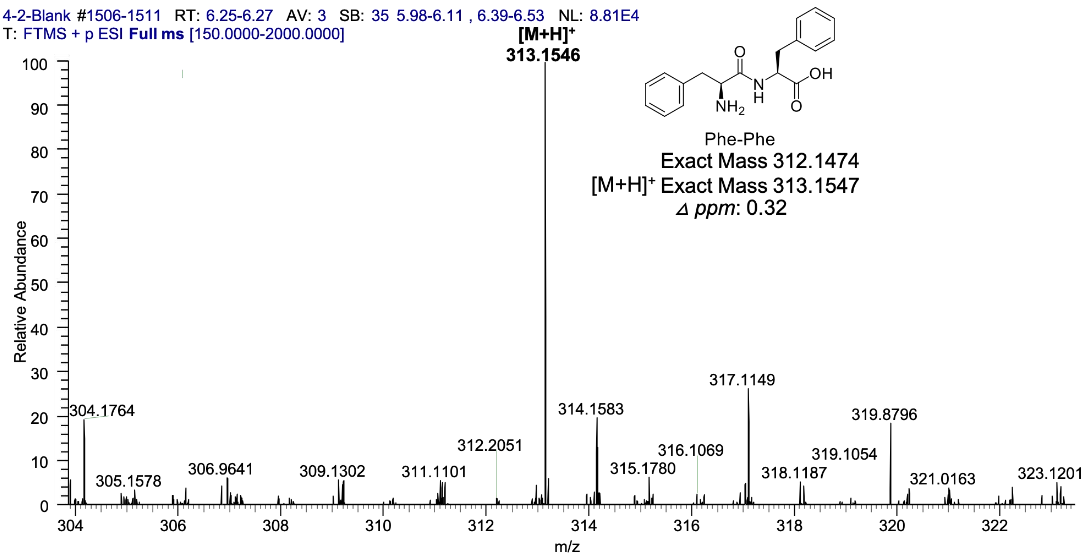

Supplementary Figure 111. The MS spectrum of the product Phe-Phe (*m/z* 313.1546) in the absence of forsterite (Blank control).

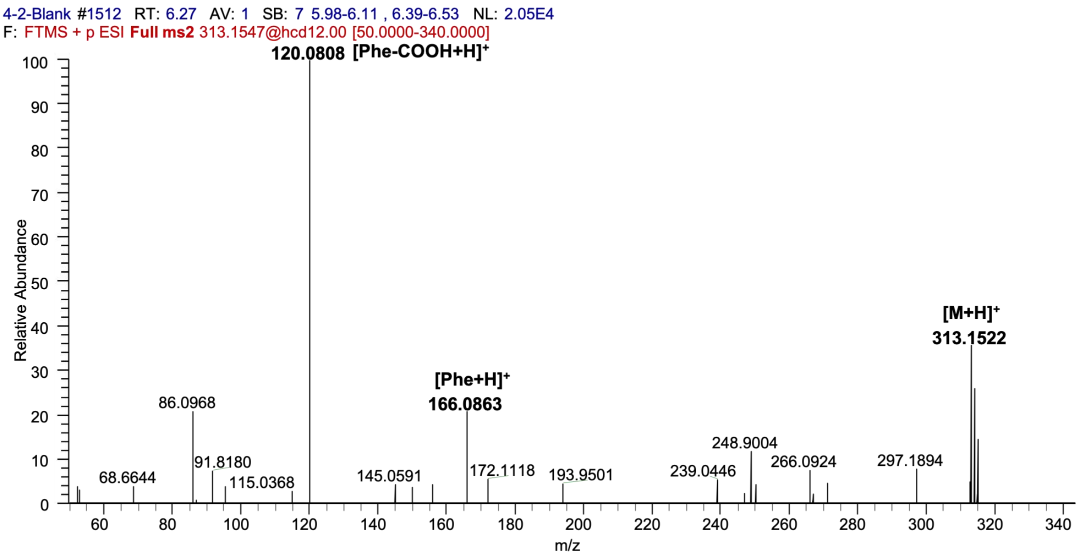

Supplementary Figure 112. The MS^2 spectrum of Phe-Phe [M+H]$^+$ ion (*m/z* 313.1547) in **Supplementary Figure 111**.

13. MS analysis in the mixed Phe and P3m reaction system of the ground condition

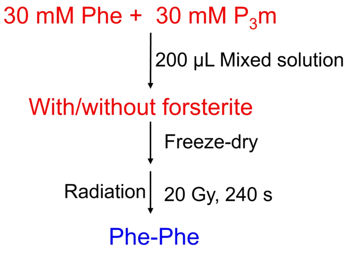

The solutions of 30 mmol/L P3m and 30 mmol/L Phe were thoroughly mixed with an equal volume of water in a 1.5 mL EP tube. To aliquot 200 μL of the aforementioned mixed solution into sample containers, respectively. Among the above two sample containers, the container containing forsterite was selected as the experimental group, while another container without forsterite was used as the blank control group. Subsequently, all the samples were freeze-dried by a freeze-dryer. The irradiation dose of 20 Gy was exposure factors of 160 kV, 25 mA, and 240 s, without 0.3 mm copper by an RS2000 X-ray biological irradiator.

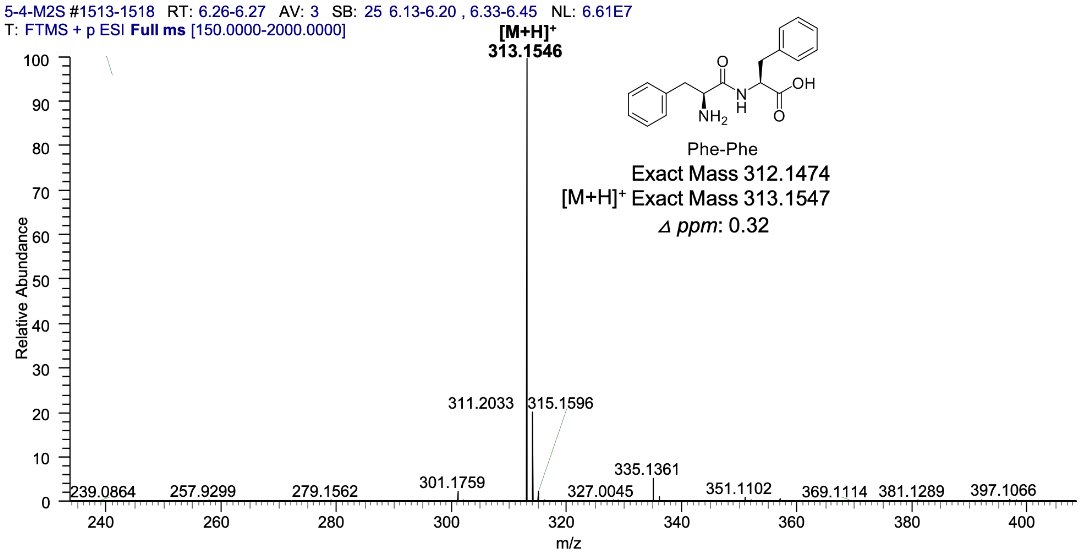

Supplementary Figure 113. The MS spectrum of the product Phe-Phe (*m/z* 313.1546) in the presence of forsterite.

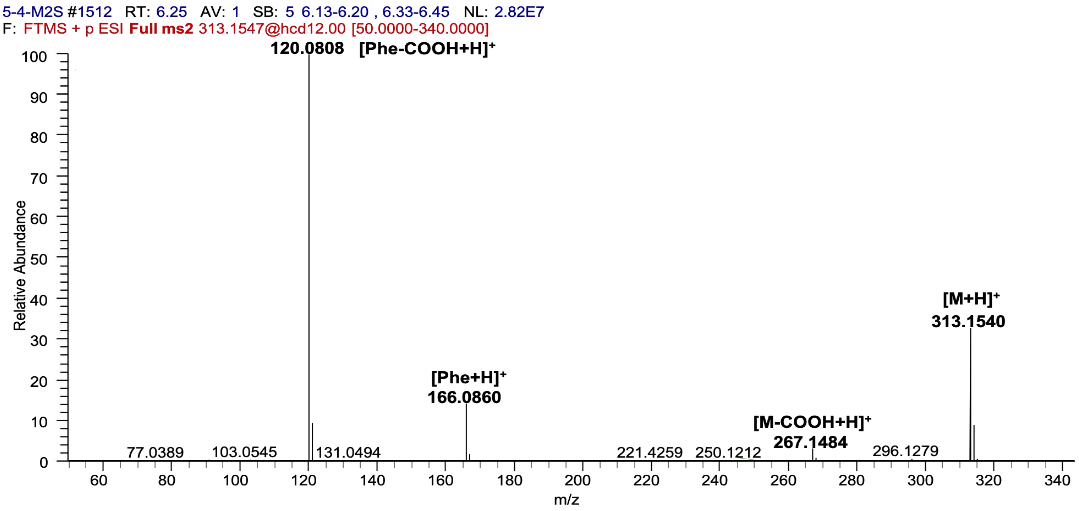

Supplementary Figure 114. The MS^2 spectrum of Phe-Phe $[M+H]^+$ ion (*m/z* 313.1547) in **Supplementary Figure 113**.

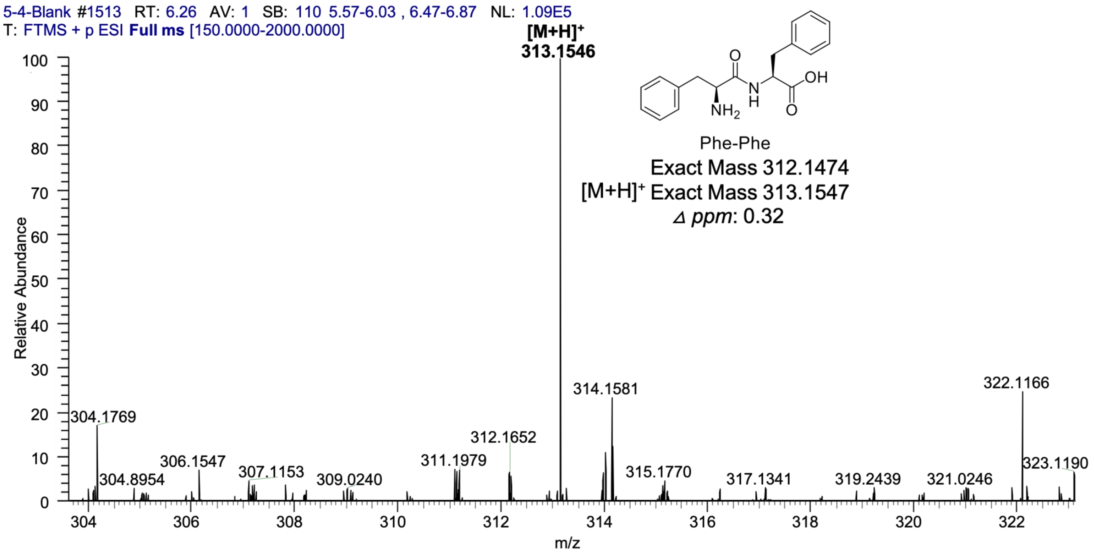

Supplementary Figure 115. The MS spectrum of the product Phe-Phe (*m/z* 313.1546) in the absence of forsterite (Blank control).

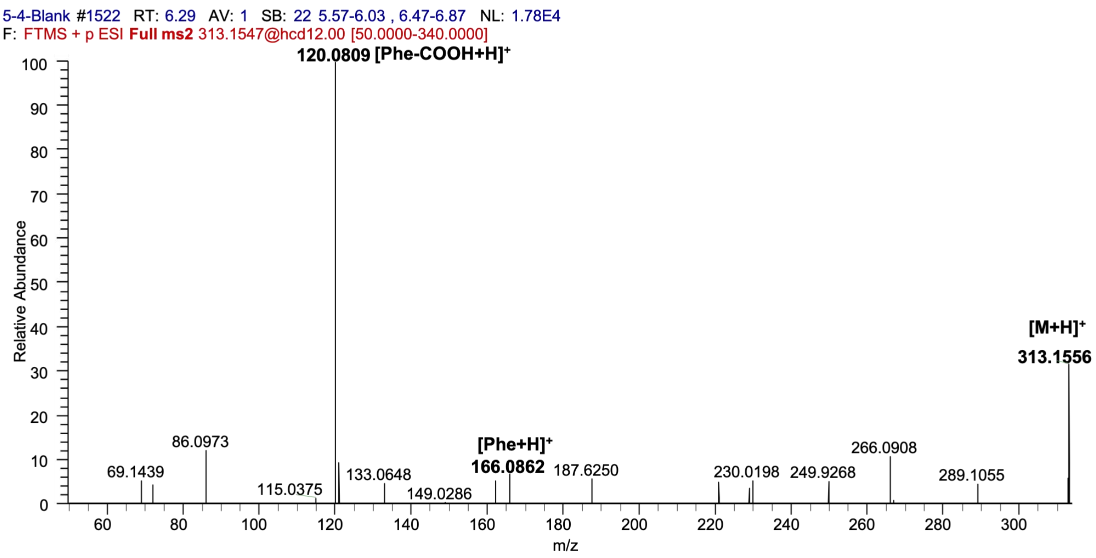

Supplementary Figure 116. The MS^2 spectrum of Phe-Phe $[M+H]^+$ ion (*m/z* 313.1547) in **Supplementary Figure 115**.

14. MS analysis in the Phe reaction system without P3m under the ground condition

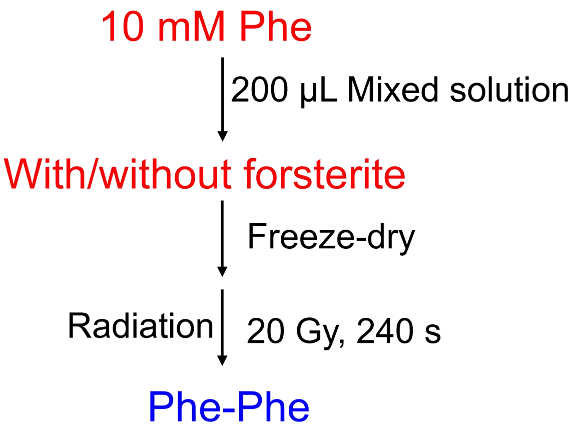

The 10 mmol/L Phe solution was thoroughly mixed in a 1.5 mL EP tube. To aliquot 200 μL of the aforementioned mixed solution into sample containers, respectively. Among the above two sample containers, the container containing forsterite was selected as the experimental group, while another container without forsterite was used as the blank control group. Subsequently, all the samples were freeze-dried by a freeze-dryer. The irradiation dose of 20 Gy was exposure factors of 160 kV, 25 mA,

and 240 s, without 0.3 mm copper by RS2000 X-rays biological irradiator.

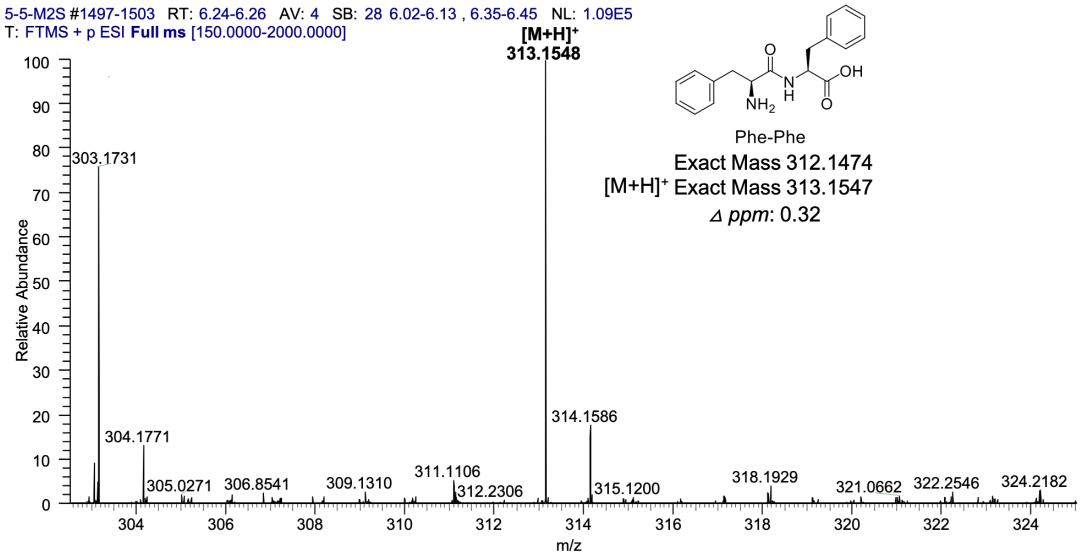

Supplementary Figure 117. The MS spectrum of the product Phe-Phe (*m/z* 313.1548) in the presence of forsterite.

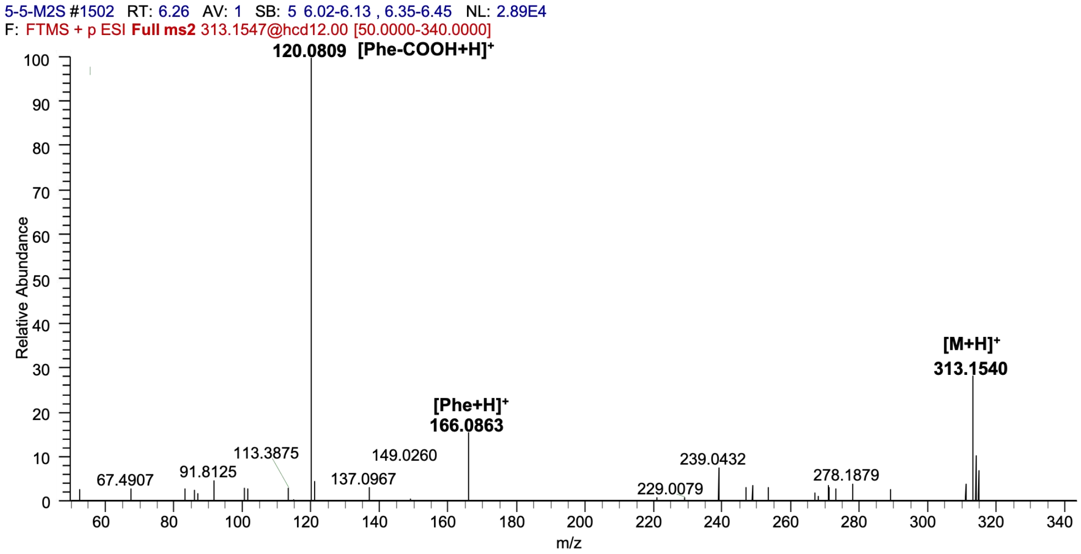

Supplementary Figure 118. The MS^2 spectrum of Phe-Phe [M+H]$^+$ ion (*m/z* 313.1547) in **Supplementary Figure 117**.

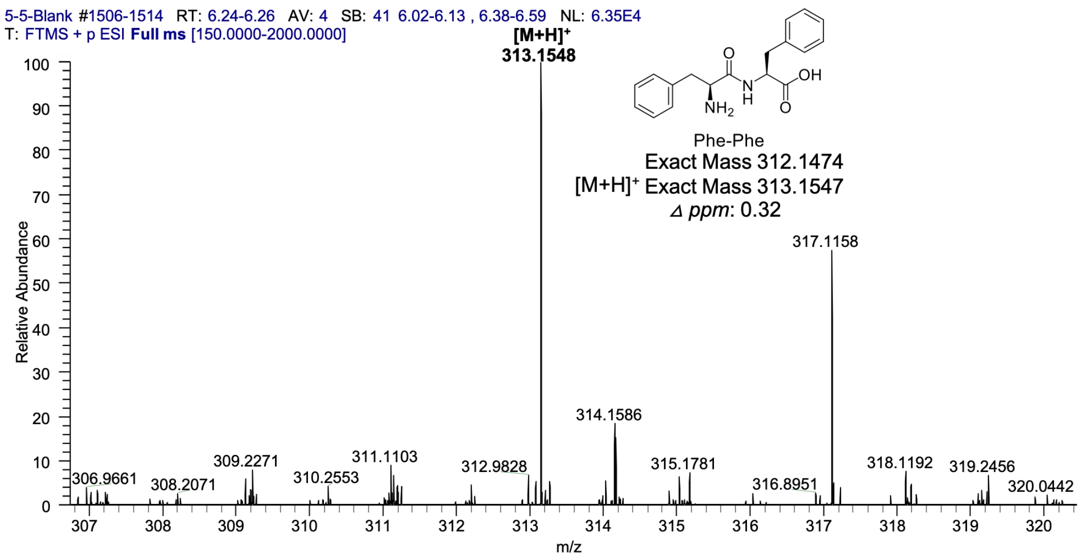

Supplementary Figure 119. The MS spectrum of the product Phe-Phe (*m/z* 313.1548) in the absence of forsterite (Blank control).

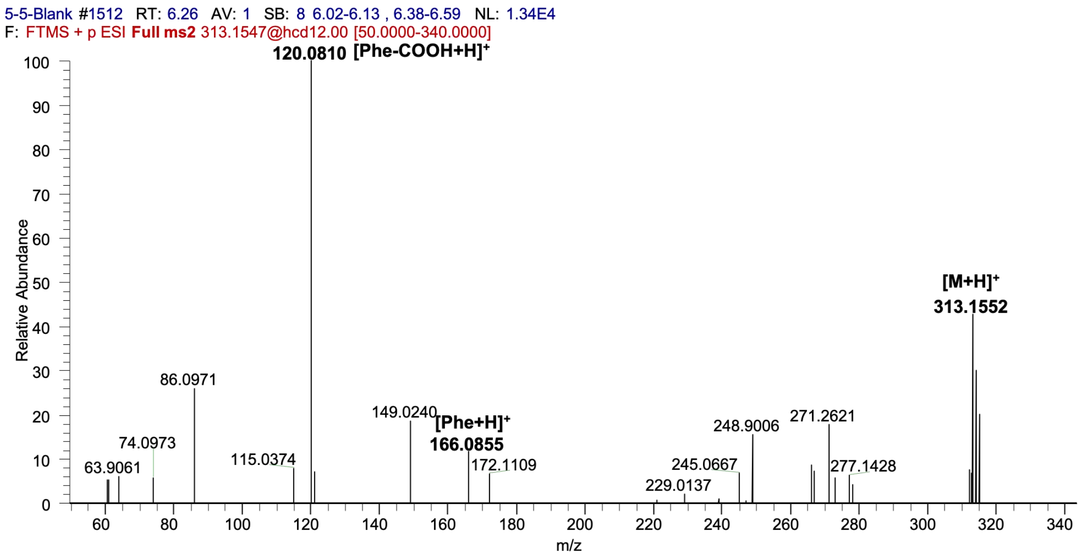

Supplementary Figure 120. The MS^2 spectrum of Phe-Phe $[M+H]^+$ ion (*m/z* 313.1547) in **Supplementary Figure 119**.

According to the above experimental results in chapters 11 to 14, the experimental results were summarized and summarized as shown in the following table (**Supplementary Table 3**):

Supplementary Table 3. The UPLC-HRMS qualitative analysis results summary of *L*-Phe reaction system ground control groups with X-ray radiation*

**Entry No.**	**Reaction system**	**Target products**	**Results**	
			Forsterite	Blank
**1**	Phe	Phe-Phe	√	√
**2**	Phe + P_3m	Phe-Phe	√	√
**3**	Phe + P_3m + A/U	Phe-Phe	√	√
		N-Phe-AMP [a]	√	n.d.
		N-Phe-UMP [a]	√	n.d.
		AMP [b]	√	√
		UMP [b]	√	n.d
**4**	Phe + P_3m + C/G	Phe-Phe	√	√
		N-Phe-CMP [a]	√	√
		N-Phe-GMP [a]	√	n.d.
		CMP [b]	n.d.	n.d.
		GMP [b]	n.d.	n.d.

Notes: *The ground control group condition: 240 s, 20 Gy. √: detected; n.d.: Not detected; Blank: The control group without any minerals. [a] *N*-NMP: 2'-Phe-NMP, 3'-Phe-NMP and 5'-Phe-NMP (5' -*N*-Phe-NMP was dominant isomer of *N*-Phe-NMP). [b] Detected by QTrap™ 5500 LC-MS. *N*: nitrogen-atom. N: nucleoside, A/U/G/C.

15. MS analysis of the reaction system containing the mixed amino acid, nucleosides and P_3m after space ionizing radiation exposure in A2 Unit of TZ 7

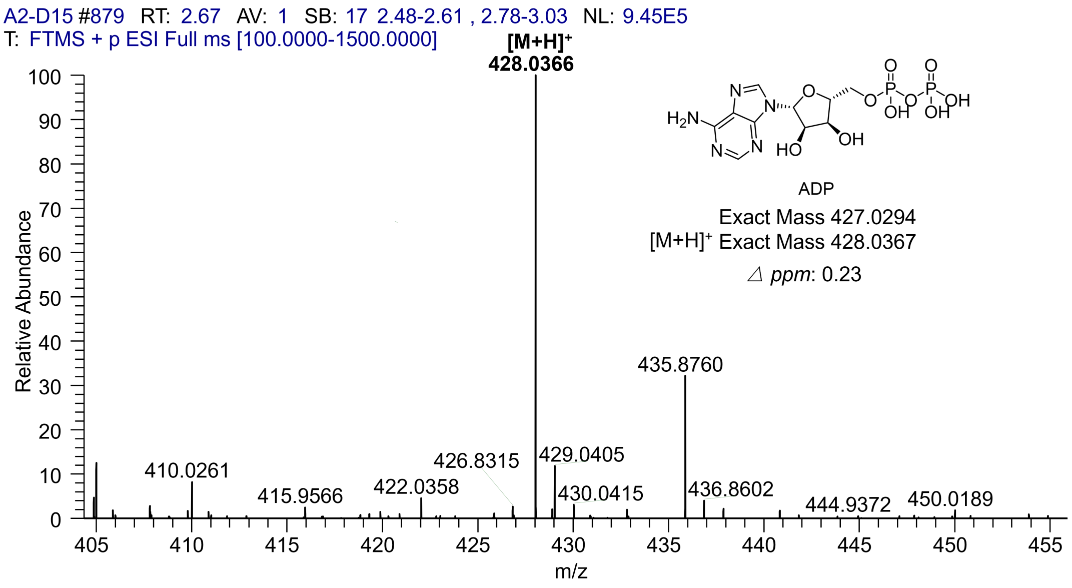

Supplementary Figure 121. The MS spectrum of 5'-ADP (*m/z* 428) product formed

from the reaction system containing Phe, nucleosides (A and U) and P3m with forsterite under the CSS radiation with a total dose of 53.50 mGy in A2 Unit of the TZ 7 launch mission (extravehicular exposure for 174 days).

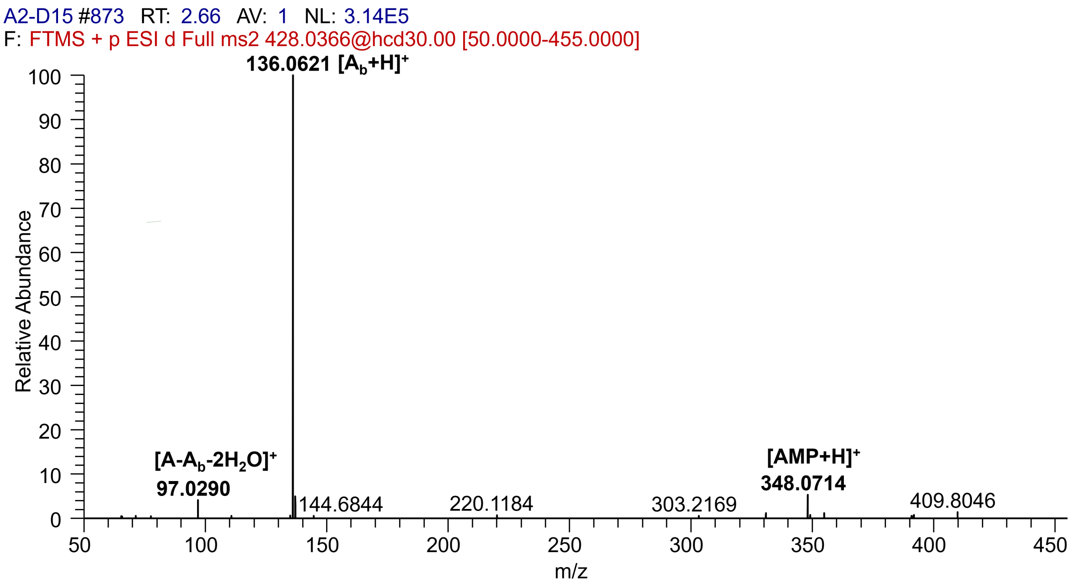

Supplementary Figure 122. The MS^2 spectrum of 5'-ADP $[M+H]^+$ ion (*m/z* 428.03) in **Supplementary Figure 121**. A: Adenosine; A_b: The base adenine.

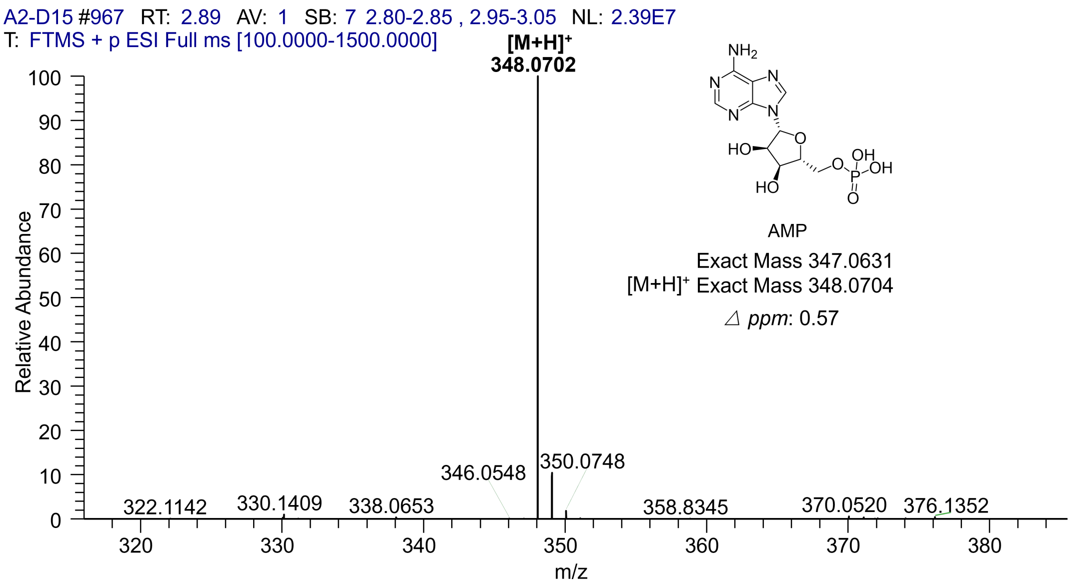

Supplementary Figure 123. The MS spectrum of 5'-AMP (*m/z* 348) product formed from the reaction system containing Phe, nucleosides (A and U) and P3m with forsterite under the CSS radiation with a total dose of 53.50 mGy in A2 Unit of the TZ 7 launch mission (extravehicular exposure for 174 days).

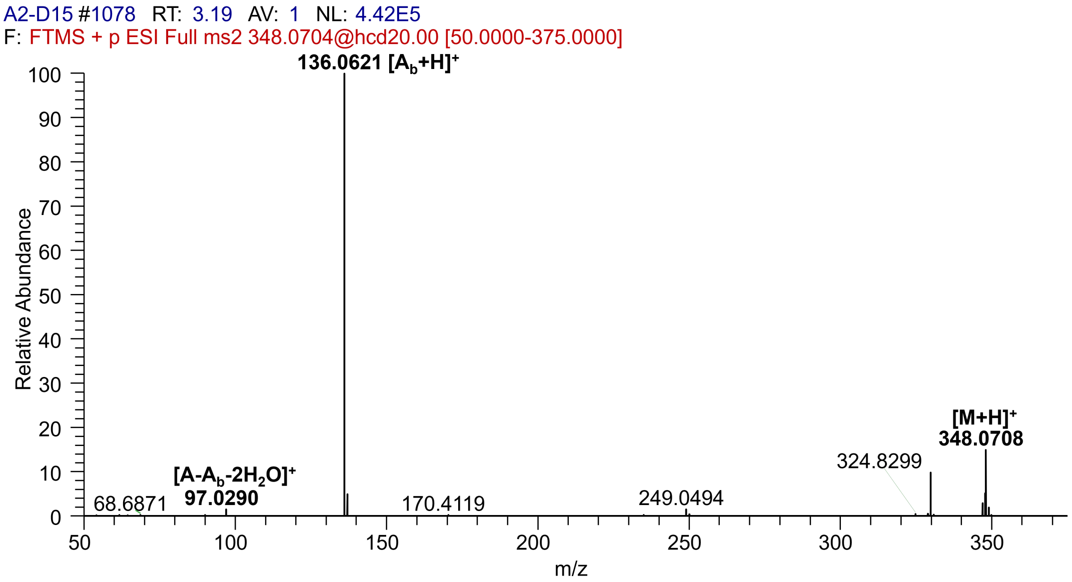

Supplementary Figure 124. The MS^2 spectrum of 5'-AMP $[M+H]^+$ ion (*m/z* 348.07) in **Supplementary Figure 123**. A: Adenosine; A_b: The base adenine.

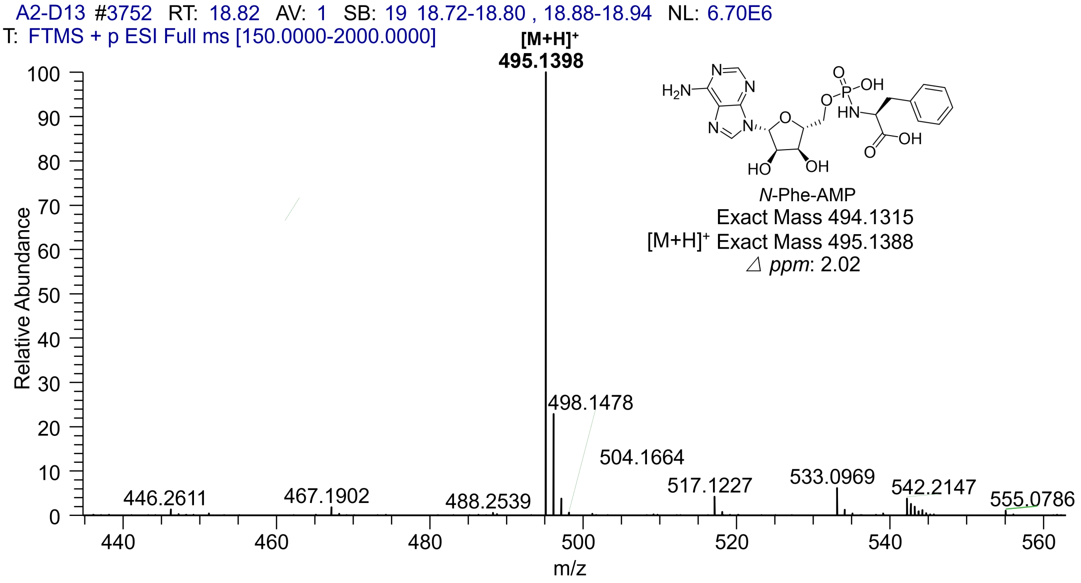

Supplementary Figure 125. The MS spectrum of 5'-*N*-Phe-AMP (*m/z* 495.13), which was formed through a solid-state reaction system facilitated by radiation and forsterite under the CSS radiation with a total dose of 53.50 mGy in A2 Unit of TZ 7 (dominant isomer of *N*-Phe-AMP, RT=18.83 min).

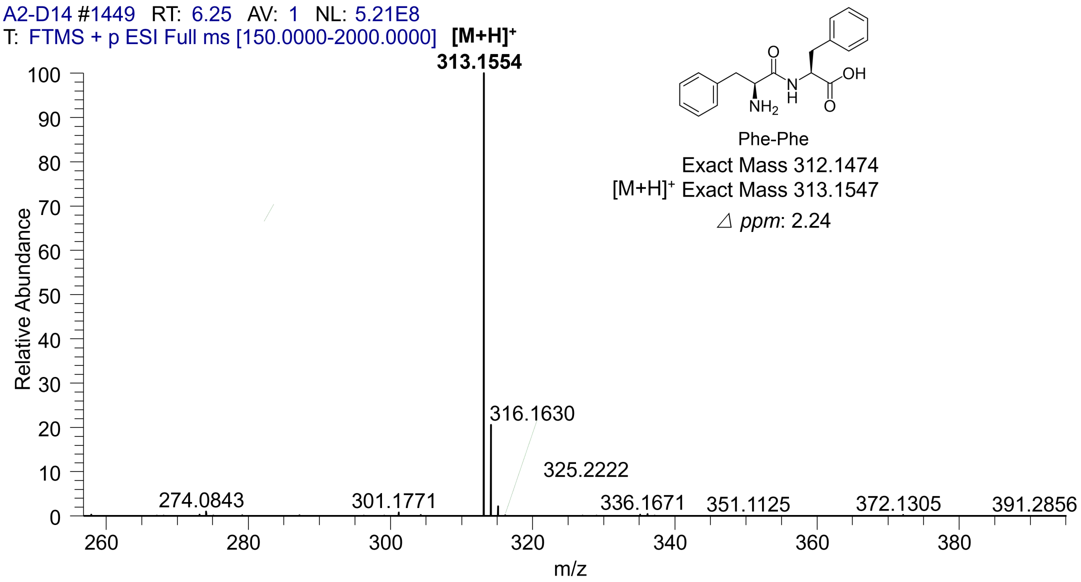

Supplementary Figure 126. The MS spectrum of Phe-Phe (*m/z* 313) product formed from the reaction system containing Phe, nucleosides (A and U) and P_3m with forsterite under the CSS radiation with a total dose of 53.50 mGy in A2 Unit of the TZ 7 launch mission (extravehicular exposure for 174 days).

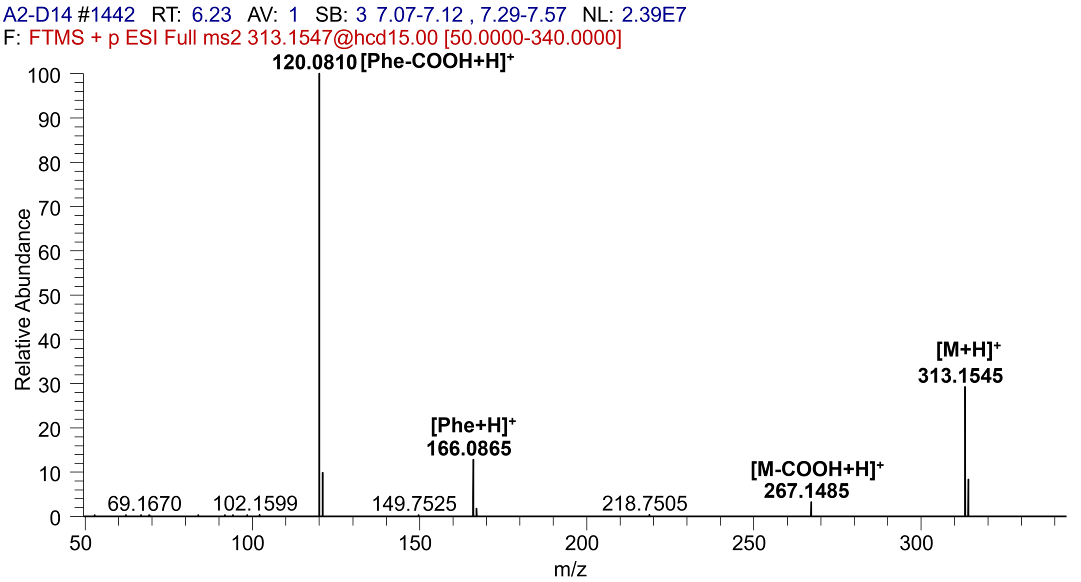

Supplementary Figure 127. The MS^2 spectrum of Phe-Phe$[M+H]^+$ ion (*m/z* 313.15) in **Supplementary Figure 126**. A: Adenosine; A_b: The base adenine.

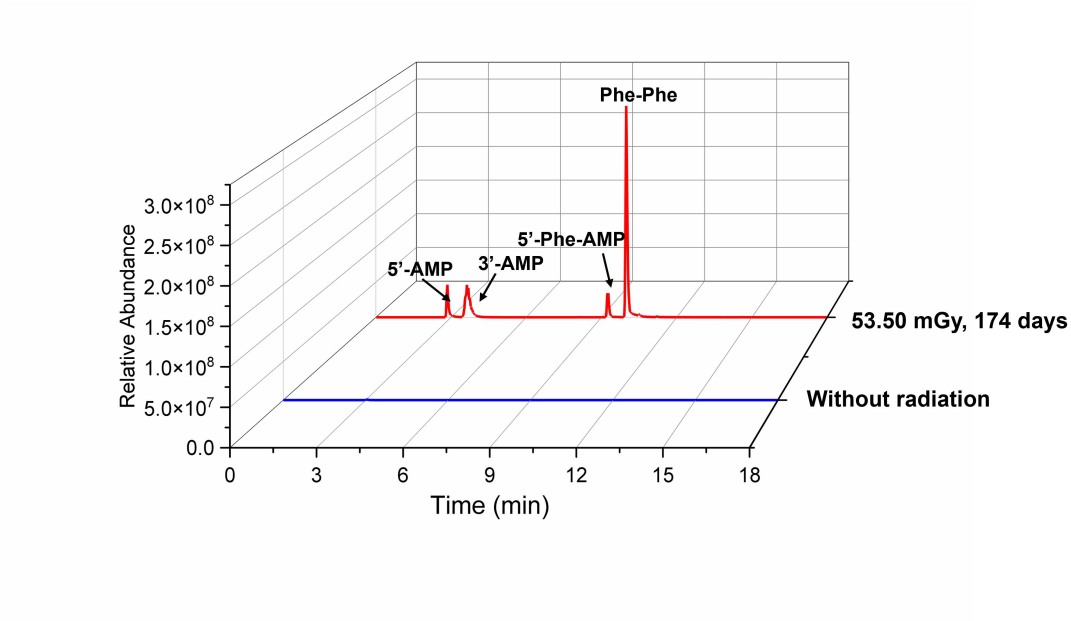

Supplementary Figure 128. The extracted ion chromatogram (EIC) of reaction products (5'-AMP, 3'-AMP, 5'-*N*-Phe-AMP, and Phe-Phe) formed from the reaction system containing Phe, nucleosides (A and U), and P_3m with forsterite under the CSS radiation with a total dose of 53.50 mGy in A2 Unit of the TZ 7 launch mission (extravehicular exposure for 174 days) and without radiation on the ground. The results demonstrate that no related products were detected in the absence of radiation.

16. Characterization of the compound *N*-Phe-AMP obtained through two different abiotic synthesis strategies by UPLC-HRMS

16.1 Formation of *N*-Phe-AMP from a reaction mixture of Phe, adenosine and P_3m with forsterite under radiation

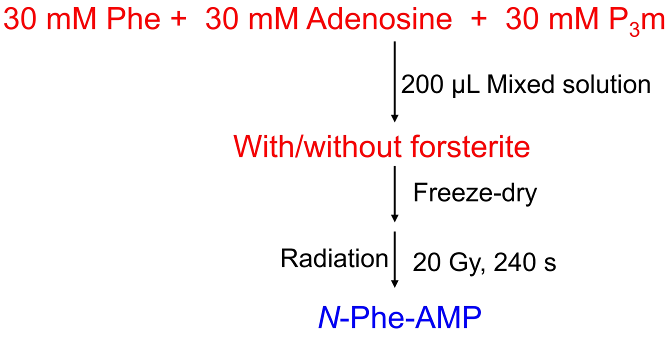

30 mmol/L P_3m and 30 mmol/L Phe solution were well-mixed with 30 mmol/L adenosine equi-volumetrically in a 1.5 mL EP tube. The above samples were 200 μL

of the aforementioned mixed solution into sample containers containing forsterite. Subsequently, all the samples were freeze-dried by a freeze-dryer. The irradiation dose of 20 Gy was exposed to factors of 160 KV, 25 mA, and 240 s, without 0.3 mm copper by an RS2000 X-ray biological irradiator. For *N*-Phe-AMP, the HPLC was fitted with an Agilent TC-C18, 5 μm, 4.6 mm × 150 mm column. The solvent A was 5 mM ammonium acetate, and solvent B was acetonitrile. The HPLC flow rate was 0.8 mL· min^{-1} with TC-C18, and the column temperature was maintained at room temperature. The gradient elution conditions were 0-14 min, 5% B; 14-20 min, 5-60% B; 20-22 min, 60% B; 22-24 min, 60-5% B; 24-30 min, 5% B. The flow rate was 0.8 mL·min^{-1}, and the DAD detector was 256 nm.

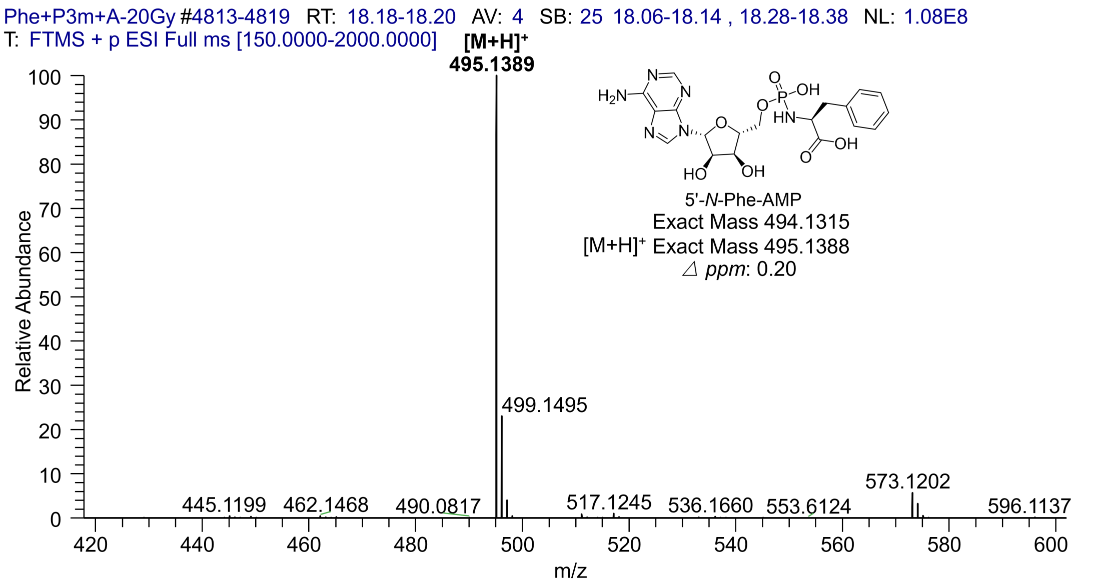

Supplementary Figure 129. The MS spectrum of the compound 5'-*N*-Phe-AMP (*m/z* 495.1389), which was formed through a solid-state reaction system facilitated by radiation and forsterite. The structure of 5' -*N*-Phe -AMP (dominant isomer of *N*-Phe-AMP, RT=18.19 min).

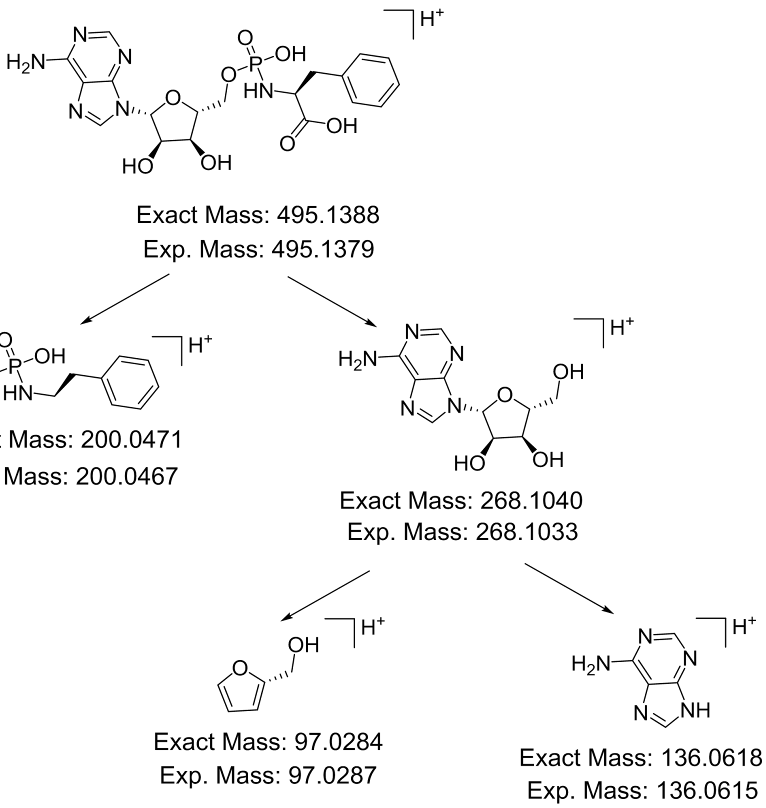

Supplementary Figure 130. The possible MS^2 fragmentation pathways of the product 5’-*N*-Phe-AMP.

16.2 Formation of *N*-Phe-AMP from a reaction mixture of Phe, adenosine and P₃m in an alkaline aqueous solution (pH 11)

$$\text{0.1 M Phe} + \text{0.1 M Adenosine} + \text{0.1 M } P_3m \xrightarrow[\text{Water, 5 h}]{45\ ^\circ C} N\text{-Phe-AMP}$$

Phenylalanine (0.1 mmol), adenosine (0.1 mmol) and trimetaphosphate (P_3m, 0.1 mmol) were dissolved in water (1 mL), and the solution was adjusted to pH 11.7 with NaOH. The solution was reacted at 45℃ for 5 h. The fraction of compound *N*-Phe-AMP was then collected according to the report results in our previous work (*Chem. Commun.*, 2018, **54**, 8598-8601) and analyzed using LC, MS and NMR systems.

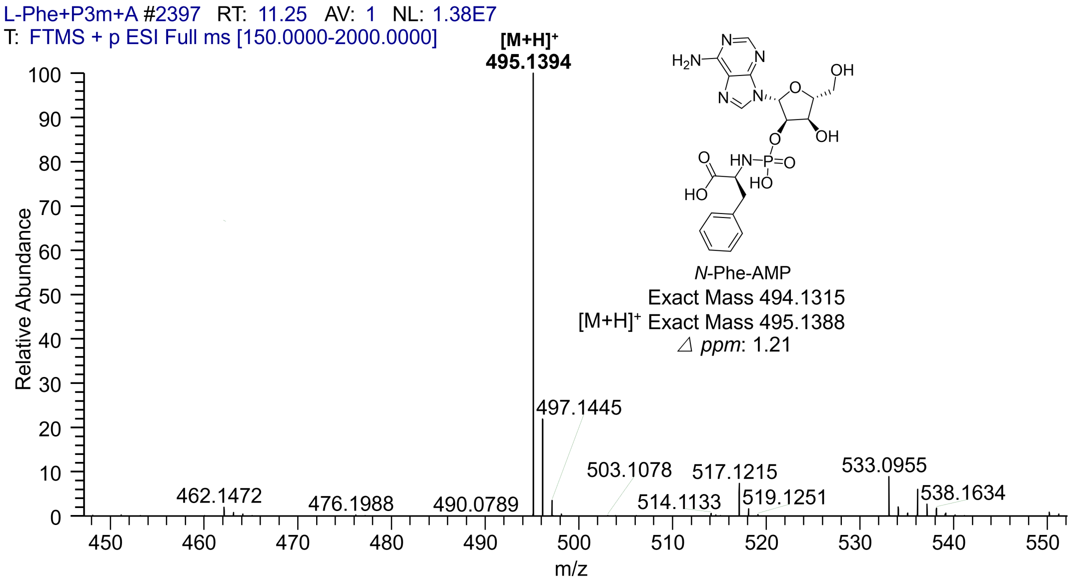

Supplementary Figure 131. The MS spectrum of 2’-*N*-Phe-AMP (*m/z* 495.1385) product formed in the alkaline aqueous (pH 11). The structure of 2’-*N*-Phe-AMP (dominant isomer of *N*-Phe-AMP, RT=11.25 min).

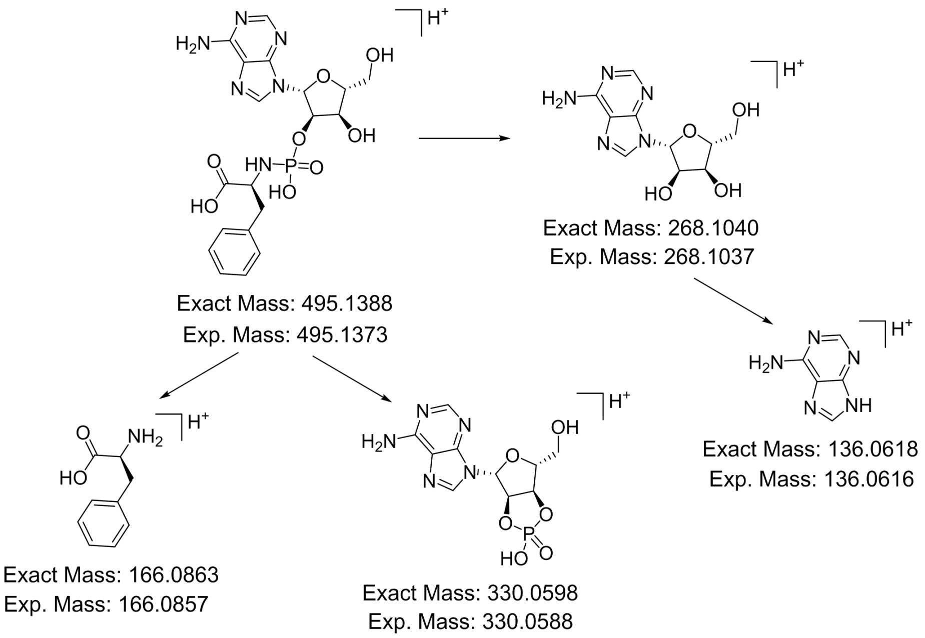

Supplementary Figure 132. The possible MS^2 fragmentation pathways of the product 2’-*N*-Phe-AMP.

17. Characterization of the compound *N*-Ile-AMP obtained through two different abiotic synthesis strategies by UPLC-HRMS

17.1 Formation of *N*-Ile-AMP from a reaction mixture of Ile, adenosine and P_3m with forsterite under radiation

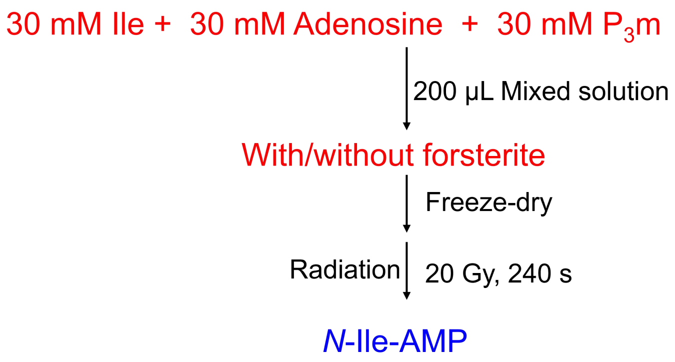

30 mmol/L P_3m and 30 mmol/L Ile solution were well-mixed with 30 mmol/L adenosine equi-volumetrically in a 1.5 mL EP tube. The above samples were 200 μL of the aforementioned mixed solution into sample containers containing forsterite. Subsequently, all the samples were freeze-dried by a freeze-dryer. The irradiation dose of 20 Gy was exposed to factors of 160 KV, 25 mA, and 240 s, without 0.3 mm copper by an RS2000 X-ray biological irradiator. The HPLC flow rate was 0.8 mL·min^{-1} with TC-C18, and the column temperature was maintained at room temperature. The gradient elution conditions were 0-9 min, 5% B; 9-11 min, 5-60% B; 11-13 min, 60-5% B; 13-18 min, 5% B. The flow rate was 0.8 mL·min^{-1}, and the DAD detector was 256 nm.

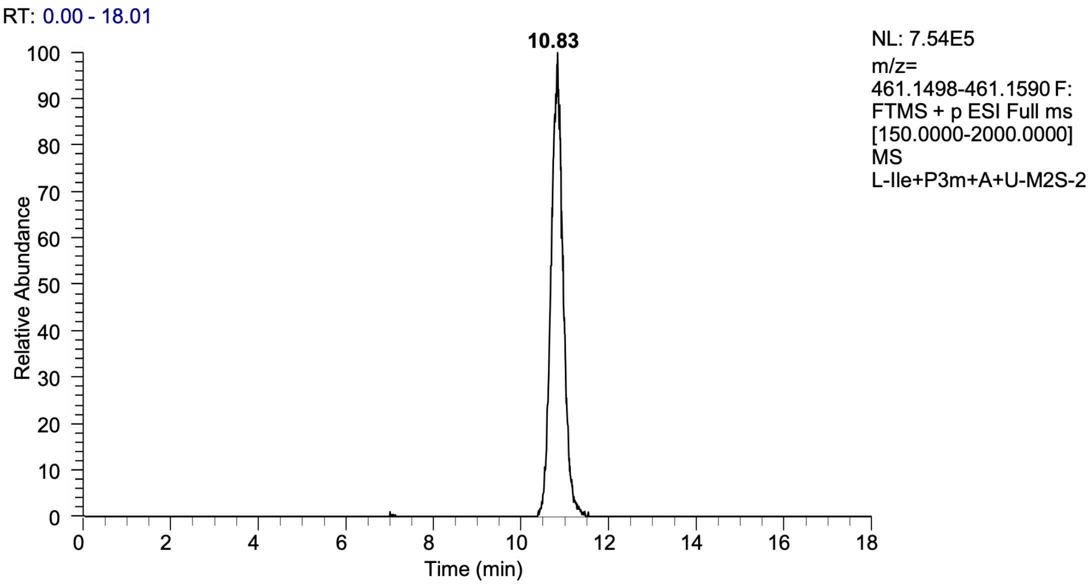

Supplementary Figure 133. The extracted ion chromatogram (EIC) of the product *N*-Ile-AMP from the forsterite-solid phase reaction system with the radiation dose of 20 Gy (240 s).

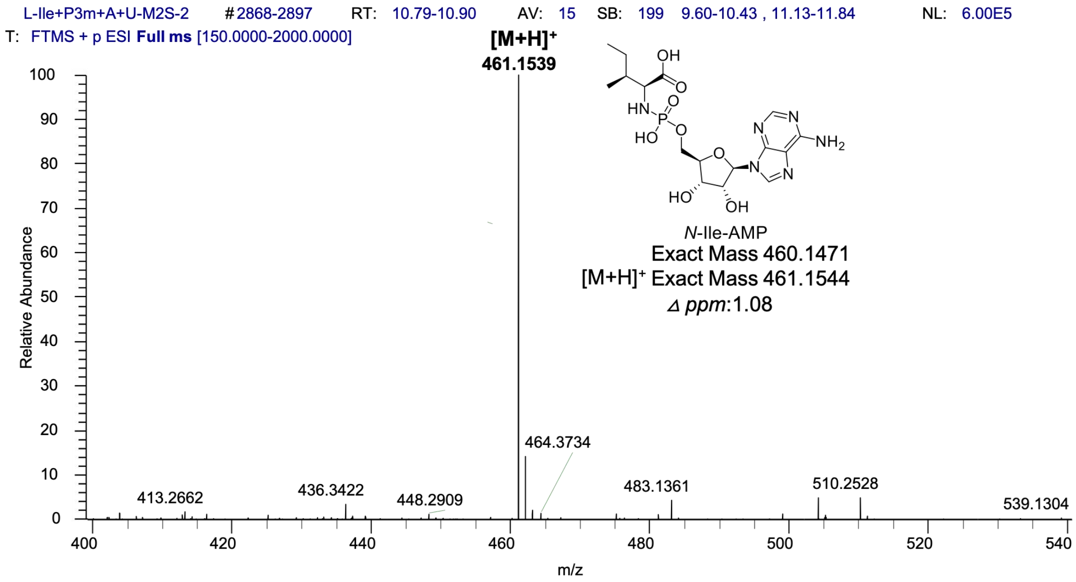

Supplementary Figure 134. The MS spectrum of the compound 5'-*N*-Ile-AMP (*m/z* 461.1539), which was formed through a solid-phase reaction system facilitated by radiation and forsterite. The structure of 5'-*N*-Ile-AMP (dominant isomer of *N*-Ile-AMP, RT=10.83 min).

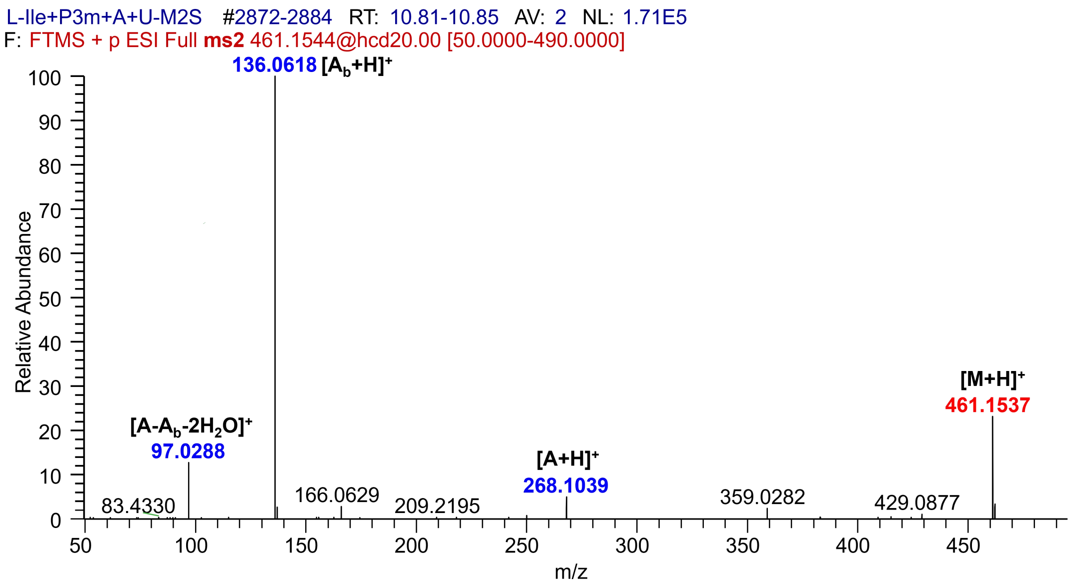

Supplementary Figure 135. The MS^2 spectrum of 5’-*N*-Ile-AMP $[M+H]^+$ ion (*m/z* 461.1537) in **Supplementary Figure 134**. A: Adenosine; A_b: The base adenine; cAMP: cyclic of adenylate.

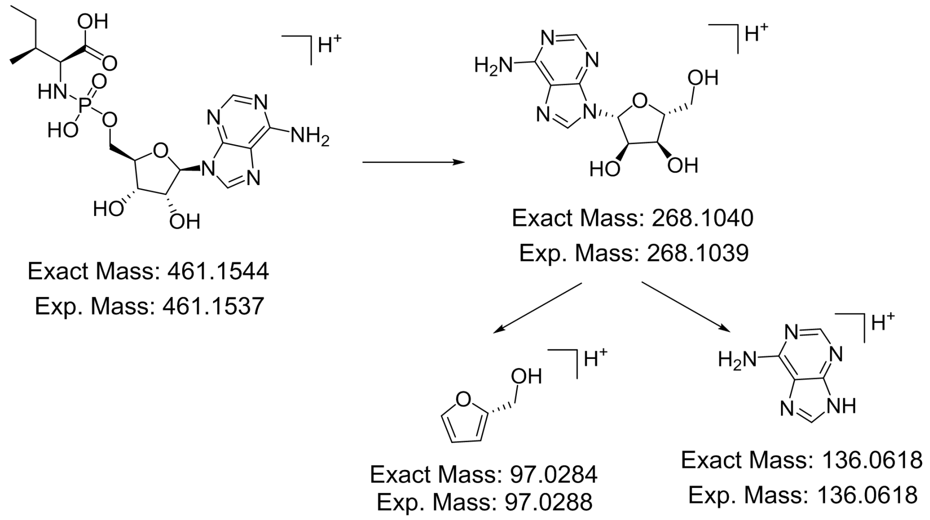

Supplementary Figure 136. The possible MS^2 fragmentation pathways of the product 5’-*N*-Ile-AMP.

17.2 Formation of *N*-Ile-AMP from a reaction mixture of Ile, adenosine and P$_3$m in an alkaline aqueous solution (pH 11)

$$\text{0.1 M Ile} + \text{0.1 M Adenosine} + \text{0.1 M } P_3m \xrightarrow[\text{Water, 5 h}]{45\ ^\circ C} N\text{-Ile-AMP}$$

Isoleucine (0.1 mmol), adenosine (0.1 mmol) and trimetaphosphate (P$_3$m, 0.1 mmol)

were dissolved in water (1 mL), and the solution was adjusted to pH 11.7 with NaOH. The solution was reacted at 45 ℃ for 5 h. The fraction of compound *N*-Ile-AMP was then collected according to the report results in our previous work (*Chin. Chem. Lett.*, 2022, **33**, 821-824) and analyzed using LC, MS and NMR systems.

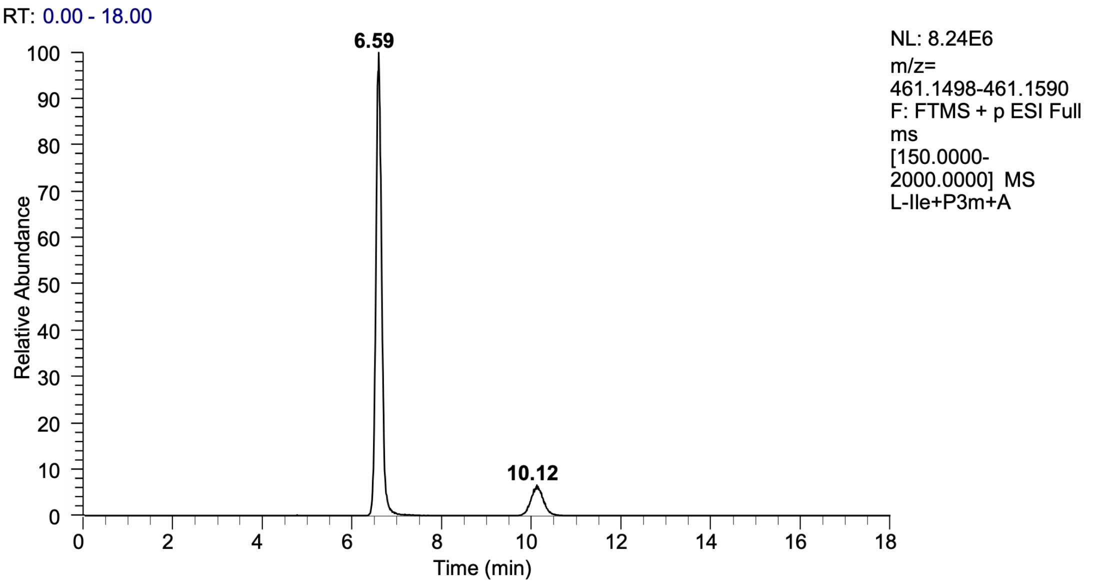

Supplementary Figure 137. The extracted ion chromatogram (EIC) of the product *N*-Ile-AMP from the alkaline aqueous solution system (pH 11).

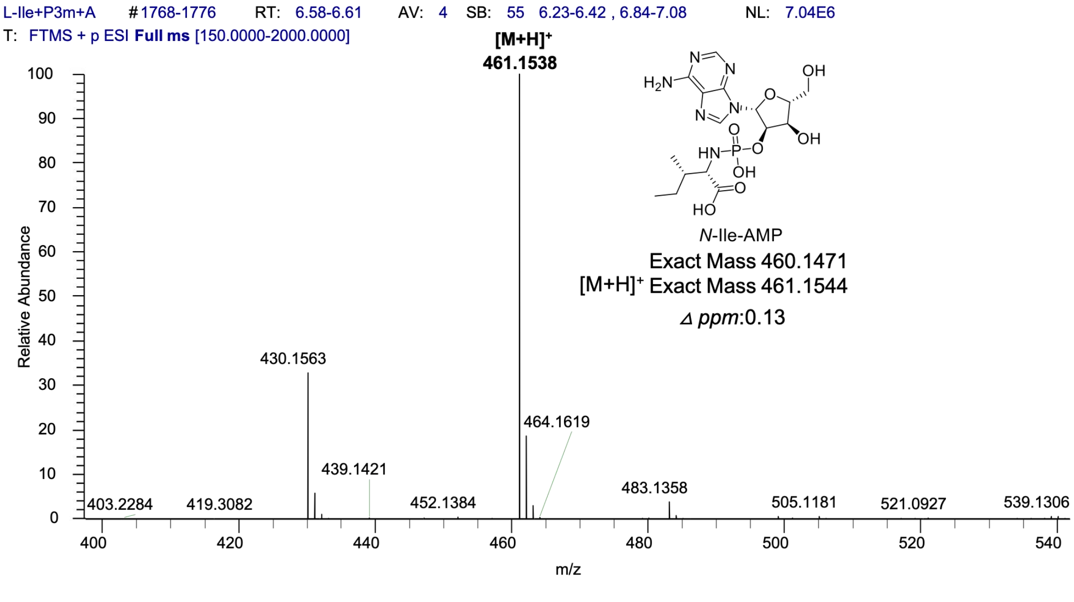

Supplementary Figure 138. The MS spectrum of *N*-Ile-AMP (*m/z* 461.1538) product formed in the alkaline aqueous (pH 11). The structure of 2'-*N*-Ile-AMP (dominant isomer of *N*-Ile-AMP, RT=6.59 min).

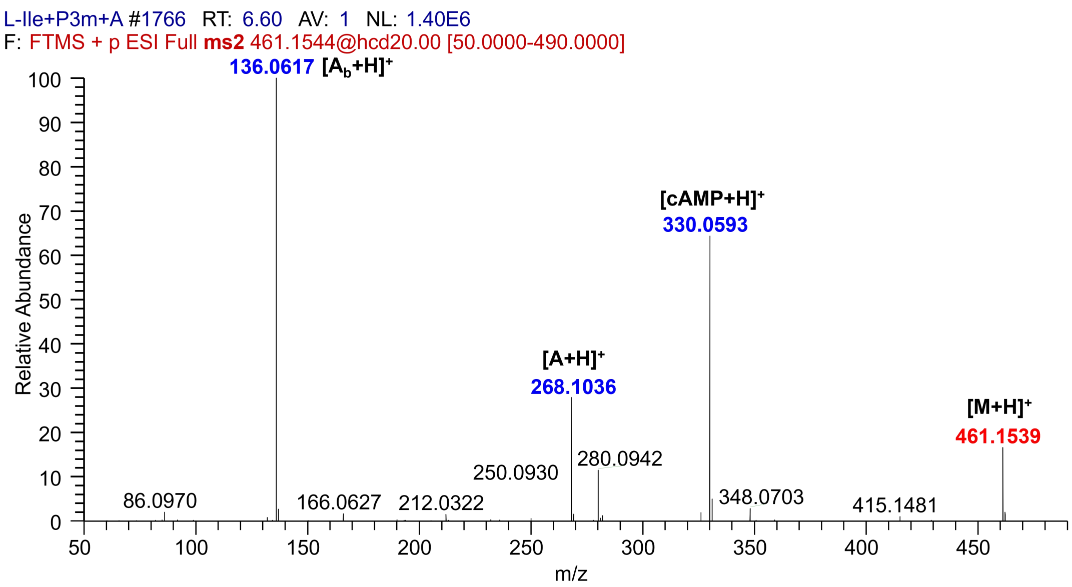

Supplementary Figure 139. The MS^2 spectrum of 2'-*N*-Ile-AMP $[M+H]^+$ ion (*m/z* 461.1537) in **Supplementary Figure 138**. A: Adenosine; A_b: The base adenine; cAMP: cyclic of adenylate.

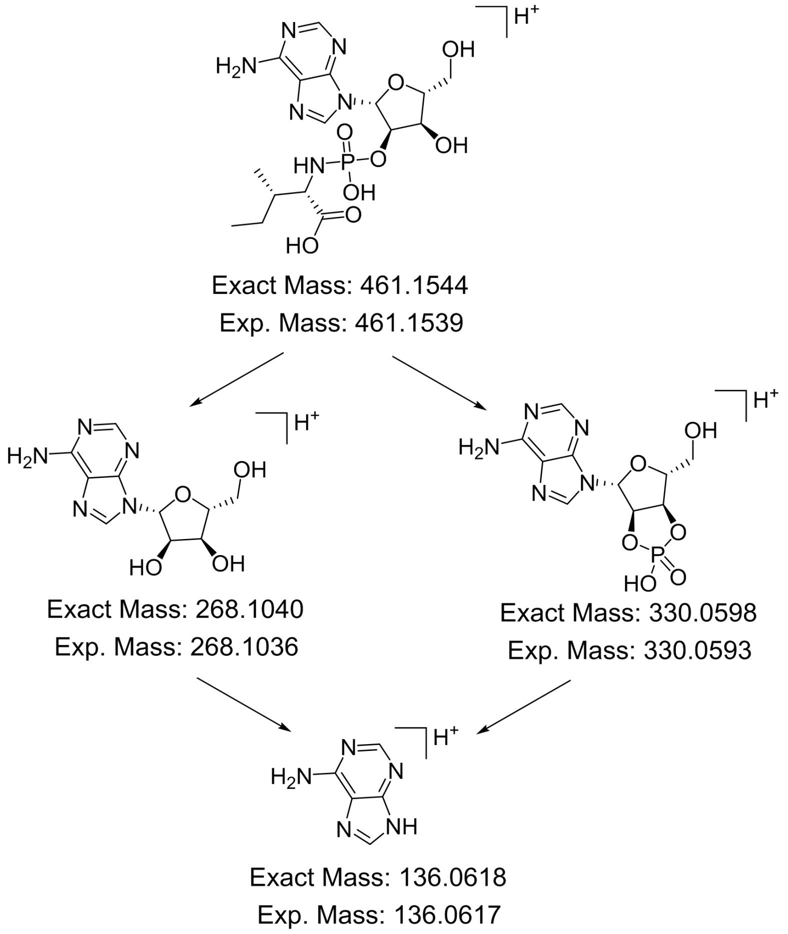

Supplementary Figure 140. The possible MS^2 fragmentation pathways of the product 2'-*N*-Ile-AMP.

18. Radical quenching experiment and the role of magnesium ion in the reaction systems

30 mmol/L Phe solutions were well-mixed with 30 mmol/L nucleosides solution (15 mmol/L Adenosine and 15 mmol/L Uridine) with an equal volume of water in a 1.5 mL EP tube. To aliquot 200 μL of the aforementioned mixed solution into two sample containers with forsterite, respectively. Adding 3 equiv. 2,2,6,6-Tetramethylpiperidinooxy (Tempo) solution as a radical quenching agent to one EP tube as the experimental group, and another one without Tempo as the control group. Subsequently, all the samples were freeze-dried by a freeze-dryer. The irradiation dose of 63.63 mGy was exposure factors of 160 kV, 5 mA, and 17 s, with 0.3 mm copper by RS2000 X-rays biological irradiator. The above experiments were carried out in parallel three times. The related products were analyzed by UPLC-HRMS. Mass spectrometry detection of the products revealed the formation of dipeptide products was not affected by the introduction of Tempo, and the free radical products captured by Tempo were also detected. It indicates that the amide and phosphoester bonds formed in the reaction system can undergo radical reactions, yet this is not the sole pathway.

30 mmol/L Phe solutions and 30 mmol/L P_3m were well-mixed with an equal volume of water in a 1.5 mL EP tube. To aliquot 200 μL of the aforementioned mixed solution into two sample containers with forsterite, respectively. Adding 3 equiv. ethylenediaminetetraacetic acid (EDTA) as a magnesium ion chelating agent to one EP tube as the experimental group, and another one without EDTA as the control group. The radiation dose of 63.63 mGy was exposure factors of 160 kV, 5 mA, and 17 s, with 0.3 mm copper by RS2000 X-rays biological irradiator. The above experiments were carried out in parallel three times. The related products were analyzed by UPLC-HRMS. The notable decrease in dipeptide yield observed upon the addition of EDTA serves as evidence that magnesium ions play a role in facilitating the peptide formation reaction. The results in the **Fig. 5c**.

30 mmol/L Phe solutions and 30 mmol/L P3m were well-mixed with an equal volume of water in a 1.5 mL EP tube. To aliquot 200 μL of the aforementioned mixed solution into three sample containers with forsterite, magnesium oxide (MgO) and silicon dioxide (SiO_2), respectively. The radiation dose of 63.63 mGy was exposure factors of 160 kV, 5 mA, and 17 s, with 0.3 mm copper by RS2000 X-rays biological irradiator. The above experiments were carried out in parallel three times. The related products were quantitative analysis of the products using an AB SCIEX QTrapTM 5500 LC–MS instrument. We observed that the peptide formation was more pronounced in the presence of magnesium oxide compared to forsterite and silicon dioxide. It indicates that the main catalytic component in forsterite is magnesium oxide. The results in the **Fig. 5d**.

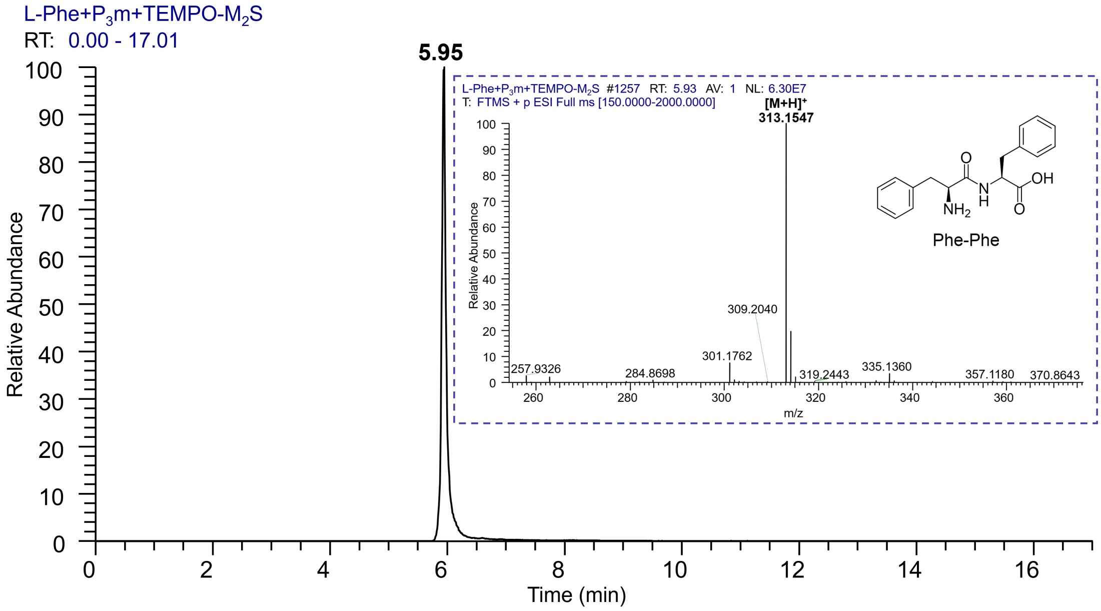

Supplementary Figure 141. Analysis results of Phe-Phe formation from Phe reaction systems with 3 equiv. Tempo conditions using UPLC-HRMS/MS. The extracted ion chromatogram (EIC) of the product Phe-Phe at *m/z* 313.1547. The insert is the MS spectrum of the product Phe-Phe (RT=5.95 min).

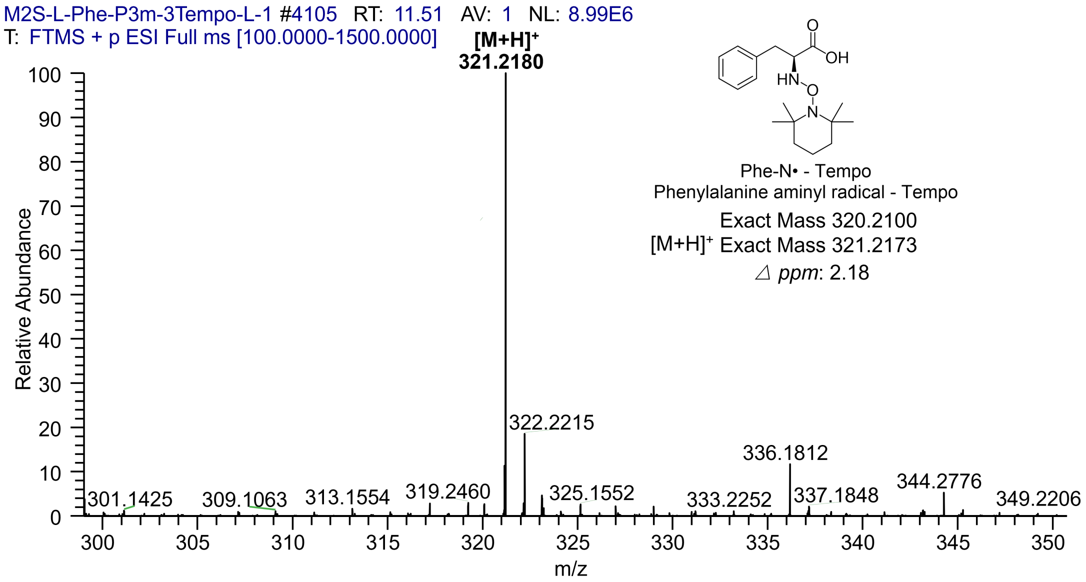

Supplementary Figure 142. The MS spectrum of Phe-N• - Tempo Phenylalanine aminyl radical - Tempo (*m/z* 321) product formed from the forsterite-solid phase with Tempo reaction system with 63.63 mGy (17 s) X-ray irradiation.

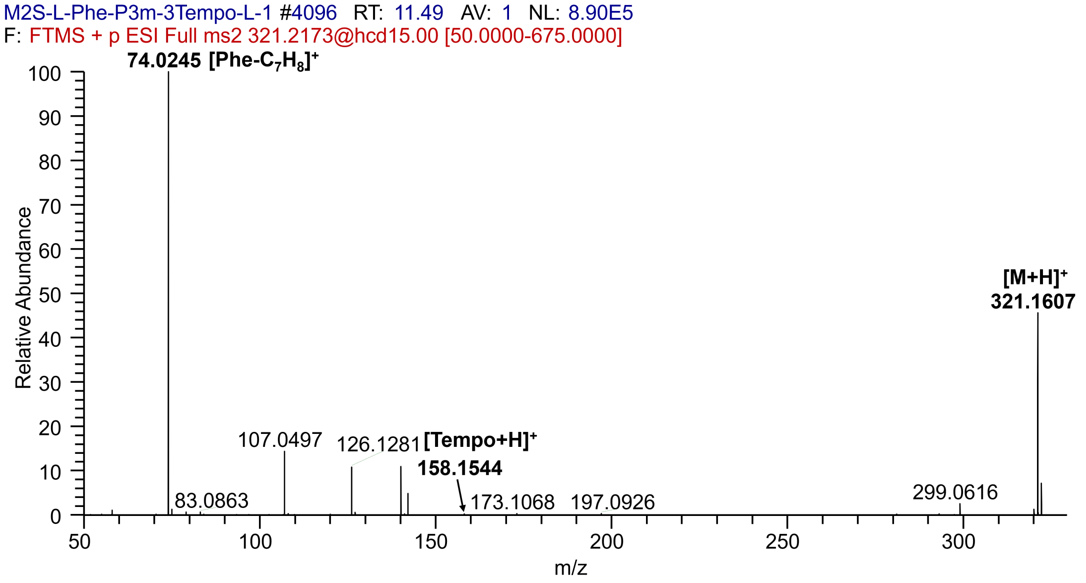

Supplementary Figure 143. The MS^2 spectrum of Phe-N• - Tempo Phenylalanine aminyl radical - Tempo $[M+H]^+$ ion (*m/z* 321.1607) in **Supplementary Figure 142**.

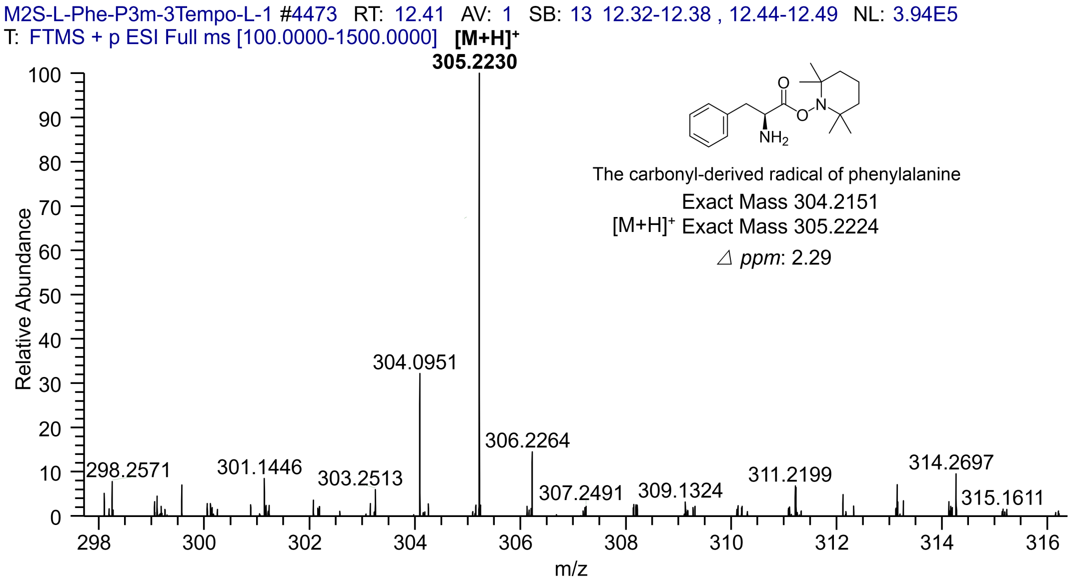

Supplementary Figure 144. The MS spectrum of the carbonyl-derived radical of phenylalanine (*m/z* 305) product formed from the forsterite-solid phase with Tempo reaction system with 63.63 mGy (17 s) X-ray irradiation.

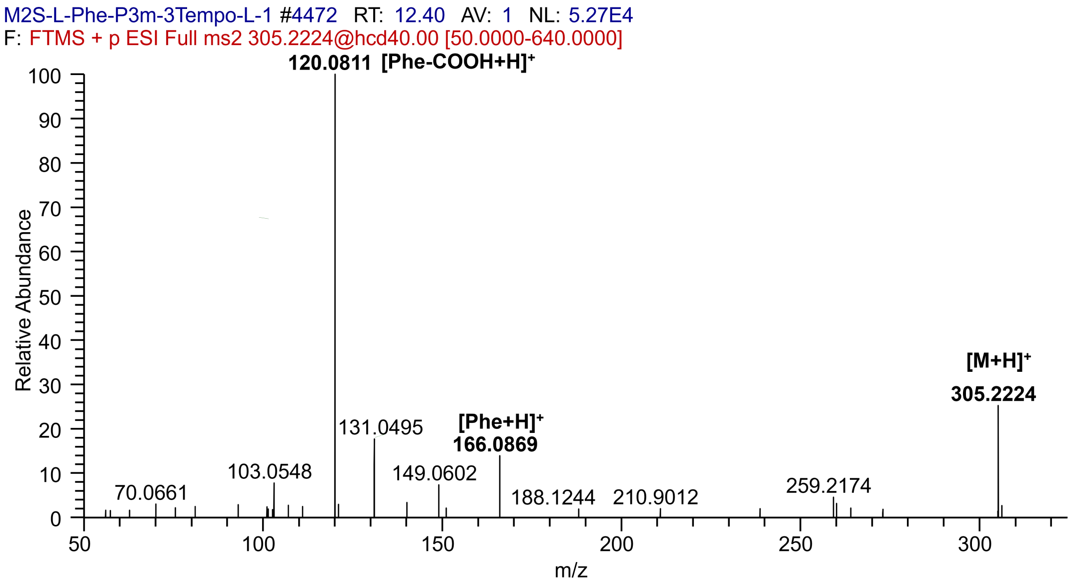

Supplementary Figure 145. The MS^2 spectrum of the carbonyl-derived radical of phenylalanine $[M+H]^+$ ion (*m/z* 305.2224) in **Supplementary Figure 144**.

19. ^{31}P NMR tracking the ring-opening reaction of P3m under X-ray radiation

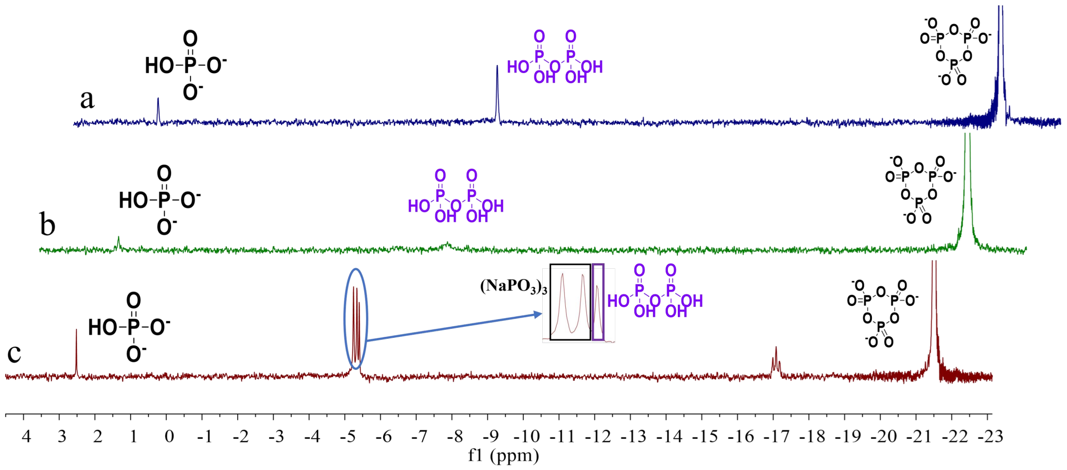

Supplementary Figure 146. ^{31}P NMR tracking the ring-opening reaction of P3m under X-ray radiation. a, The sample of A and P3m with SiO_2. b, The sample of A and P3m with forsterite. c, The sample of A and P3m with MgO. After radiation, P3m will open the ring to form monophosphate (Pi) and pyrophosphoric acid (PPi).

20. MS analysis in the mixed reaction system with Phe and hydroxyapatite under the ground radiation condition

20.1 MS analysis in the Phe and hydroxyapatite reaction system under the ground condition

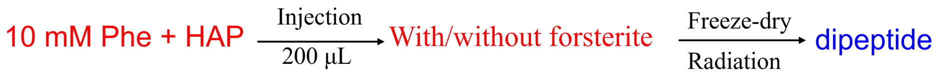

The 10 mmol/L Phe solution was thoroughly mixed with an equal volume of water in a 1.5 mL EP tube. To aliquot 200 μL of the aforementioned mixed solution into two sample containers with hydroxyapatite (HAP), respectively.

Among the above two sample containers, the container containing forsterite was selected as the experimental group, while another container without forsterite was

used as the blank control group. Subsequently, all the samples were freeze-dried by a freeze-dryer. The irradiation dose of 63.63 mGy was exposure factors of 160 kV, 5 mA, and 17 s, with 0.3 mm copper by RS2000 X-rays biological irradiator.

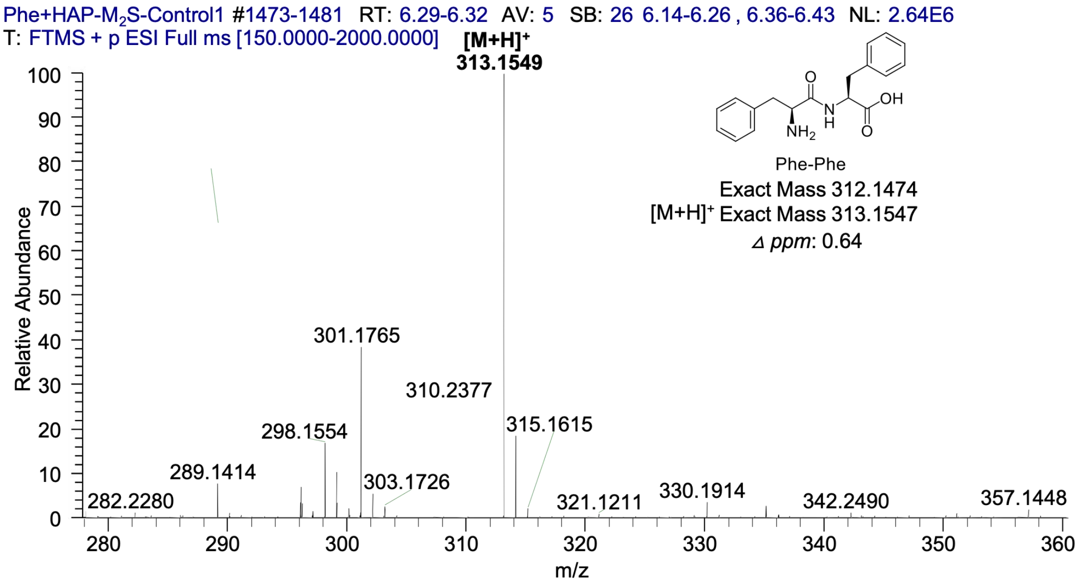

Supplementary Figure 147. The MS spectrum of the product Phe-Phe (*m/z* 313.1549) in the presence of forsterite.

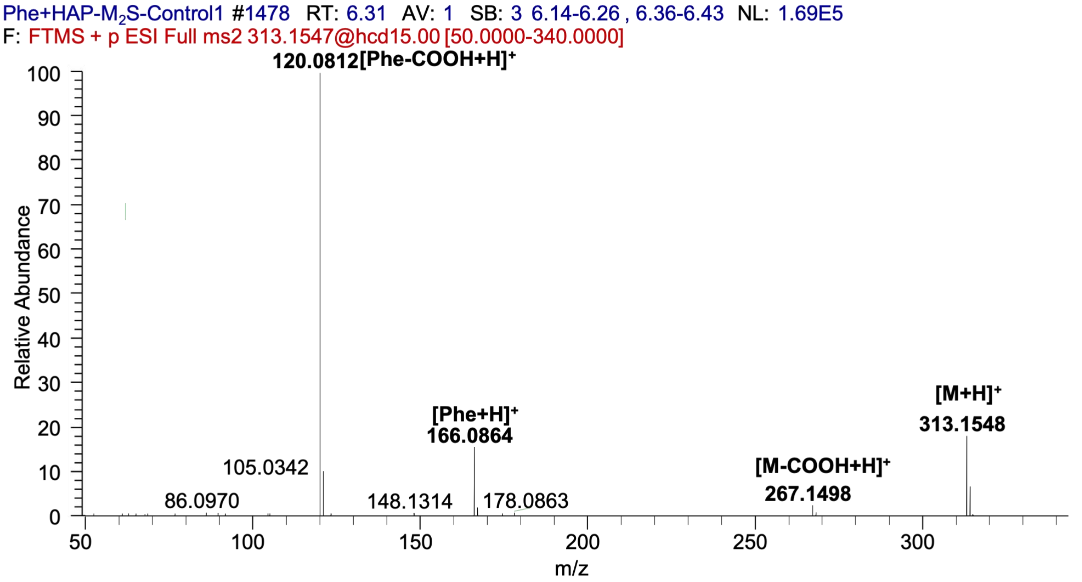

Supplementary Figure 148. The MS^2 spectrum of Phe-Phe $[M+H]^+$ ion (*m/z* 313.1548) in **Figure 147.**

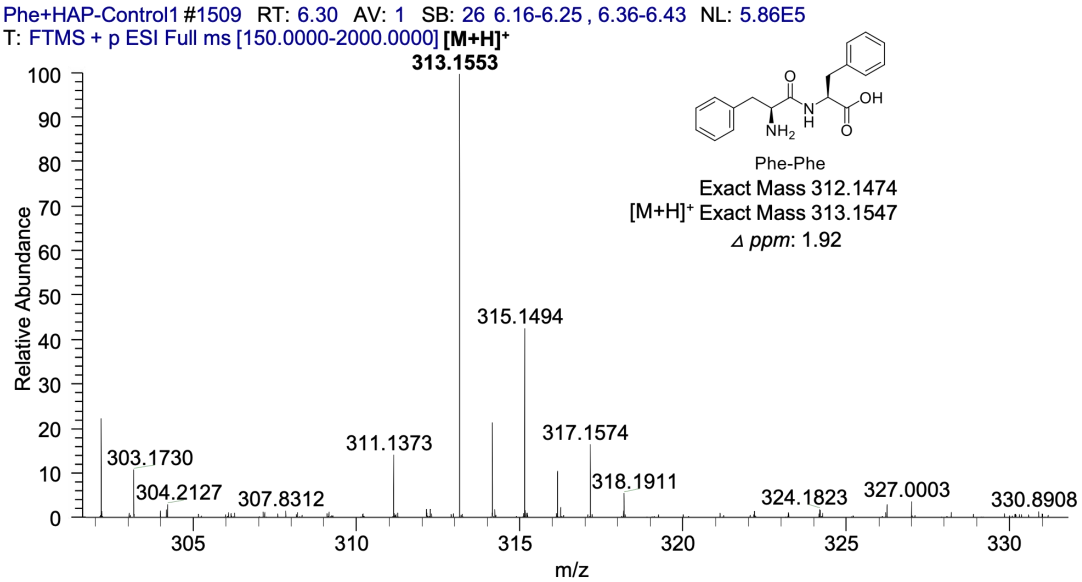

Supplementary Figure 149. The MS spectrum of the product Phe-Phe (*m/z* 313.1553) in the absence of forsterite.

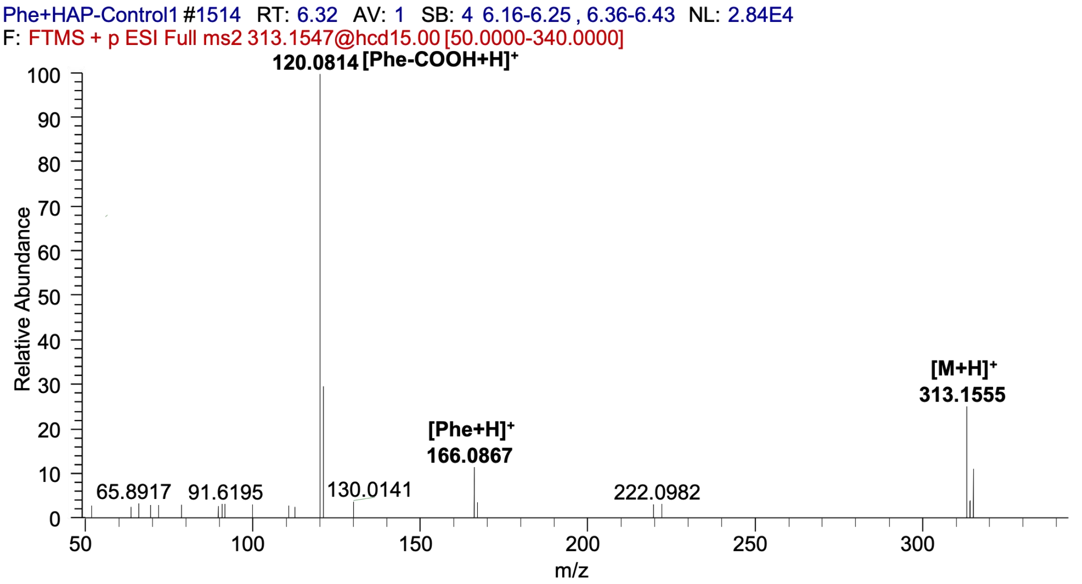

Supplementary Figure 150. The MS^2 spectrum of Phe-Phe $[M+H]^+$ ion (*m/z* 313.1555) in **Supplementary Figure 149.**

20.2 MS analysis of the products formed in the reaction system containing Phe, nucleosides (A and U), and hydroxyapatite under the ground radiation condition

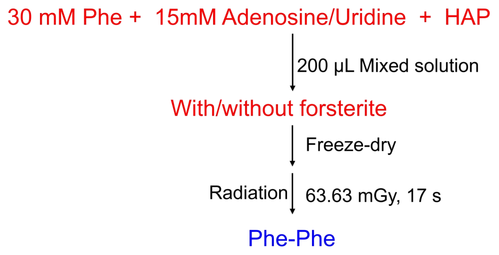

30 mmol/L Phe solutions were well-mixed with 30 mmol/L nucleosides solution (15 mmol Adenosine and 15 mmol/L Uridine) with an equal volume of water in a 1.5 mL EP tube. To aliquot 200 μL of the aforementioned mixed solution into two sample containers of hydroxyapatite, respectively. Among the above two sample containers, the container containing forsterite was selected as the experimental group, while another container without forsterite was used as the blank control group. Subsequently, all the samples were freeze-dried by a freeze-dryer. The irradiation dose of 63.63 mGy was exposure factors of 160 kV, 5 mA, and 17 s, with 0.3 mm copper by RS2000 X-rays biological irradiator.

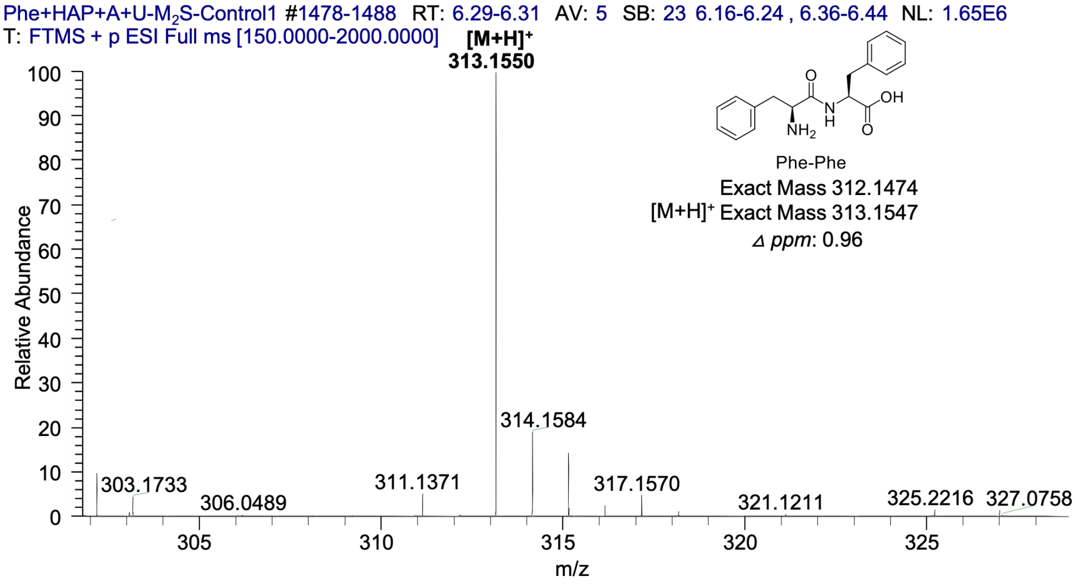

Supplementary Figure 151. The MS spectrum of the product Phe-Phe (*m/z* 313.1550) in the presence of forsterite.

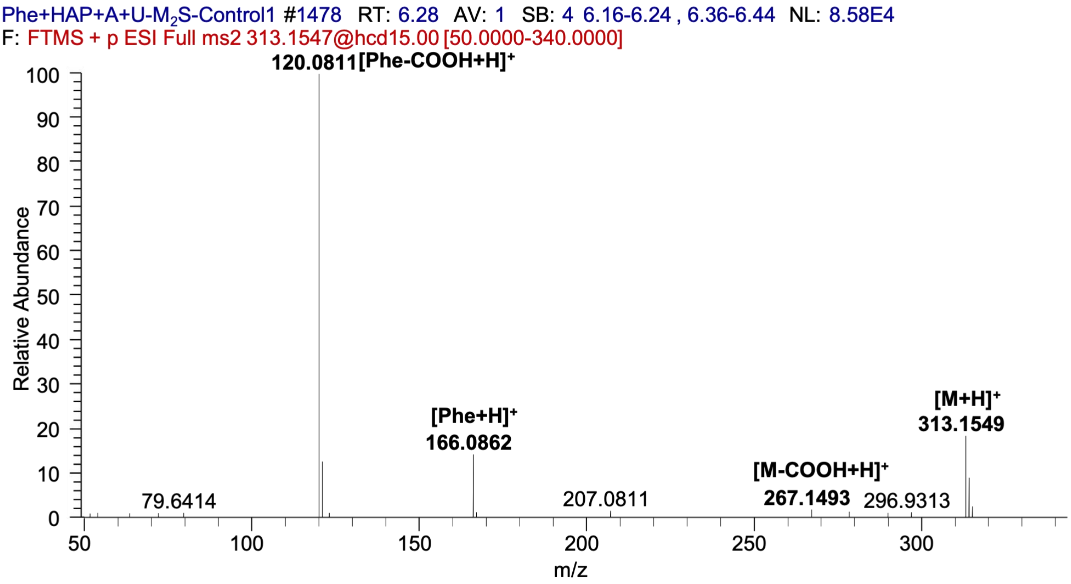

Supplementary Figure 152. The MS^2 spectrum of Phe-Phe $[M+H]^+$ ion (*m/z* 313.1549) in **Supplementary Figure 151.**

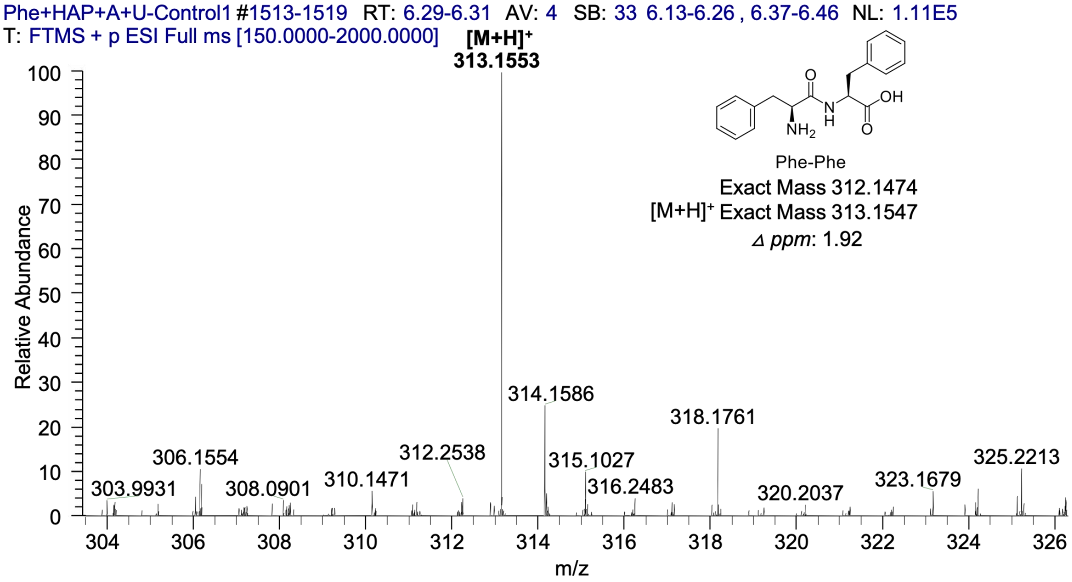

Supplementary Figure 153. The MS spectrum of the product Phe-Phe (*m/z* 313.1553) in the absence of forsterite.

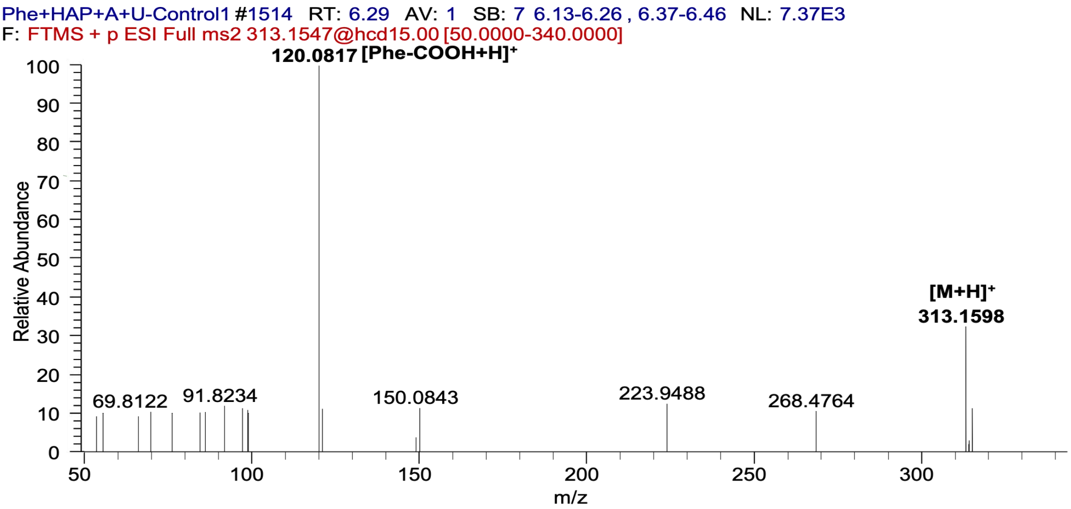

Supplementary Figure 154. The MS^2 spectrum of Phe-Phe $[M+H]^+$ ion (*m/z* 313.1598) in **Supplementary Figure 153**.

20.3 MS analysis of the products formed in the reaction system containing Phe, nucleosides (C and G), and hydroxyapatite under the ground radiation conditions

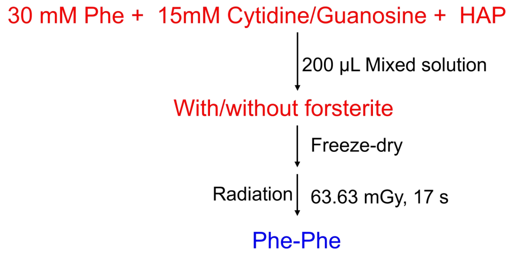

30 mmol/L Phe solutions were well-mixed with 30 mmol/L nucleosides solution (15 mmol/L Cytidine and 15 mmol/L Guanosine) with an equal volume of water in a 1.5 mL EP tube. To aliquot 200 μL of the aforementioned mixed solution into two sample containers with hydroxyapatite, respectively. Among the above two sample containers, the container containing forsterite was selected as the experimental group, while another container without forsterite was used as the blank control group. Subsequently, all the samples were freeze-dried by a freeze-dryer. The irradiation dose of 63.63 mGy was exposure factors of 160 kV, 5 mA, and 17 s, with 0.3 mm copper by RS2000 X-rays biological irradiator.

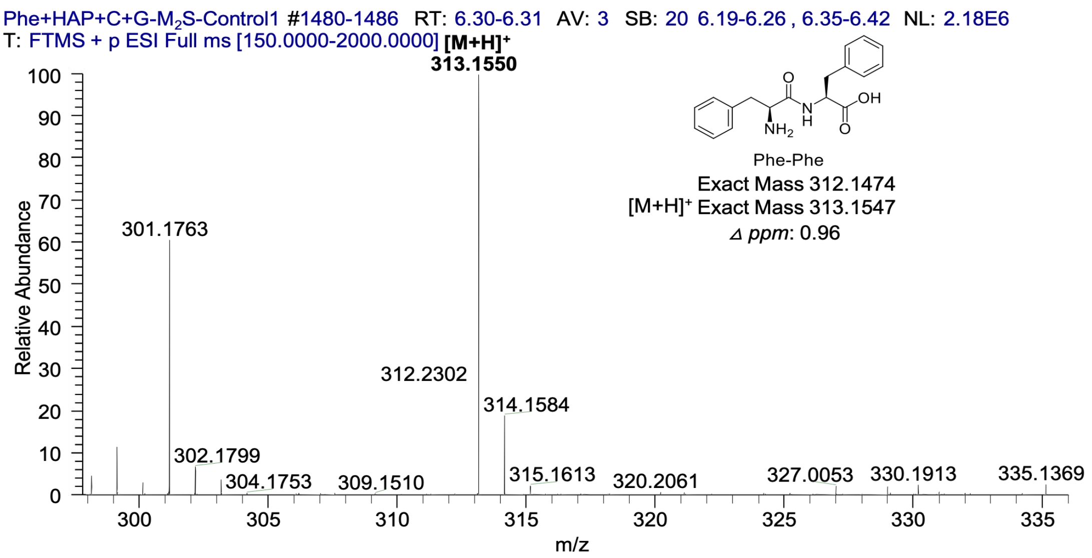

Supplementary Figure 155. The MS spectrum of the product Phe-Phe (*m/z* 313.1550) in the presence of forsterite.

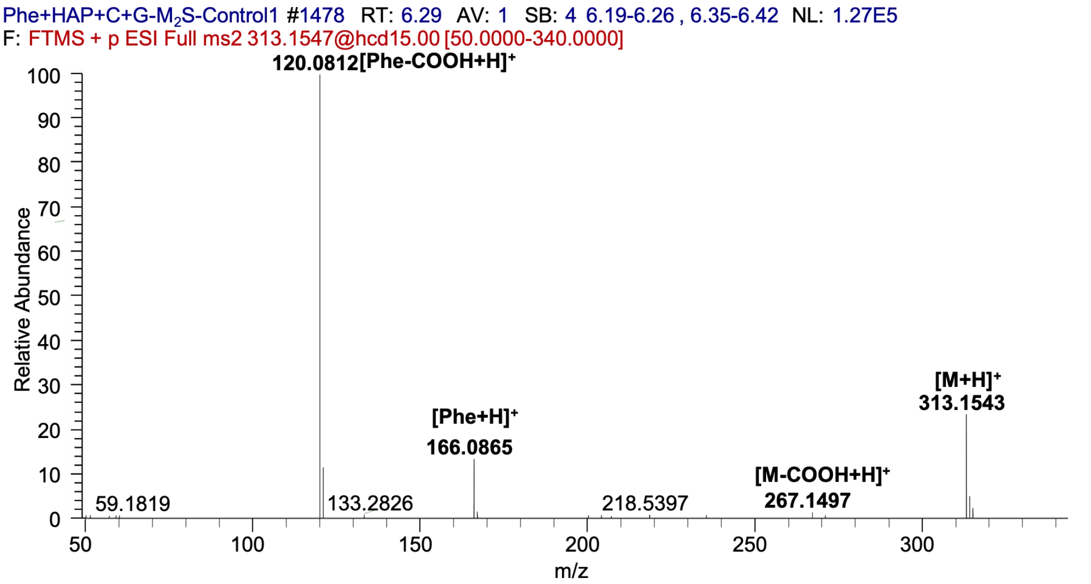

Supplementary Figure 156. The MS^2 spectrum of Phe-Phe ion (*m/z* 313.1543) in **Supplementary Figure 155**.

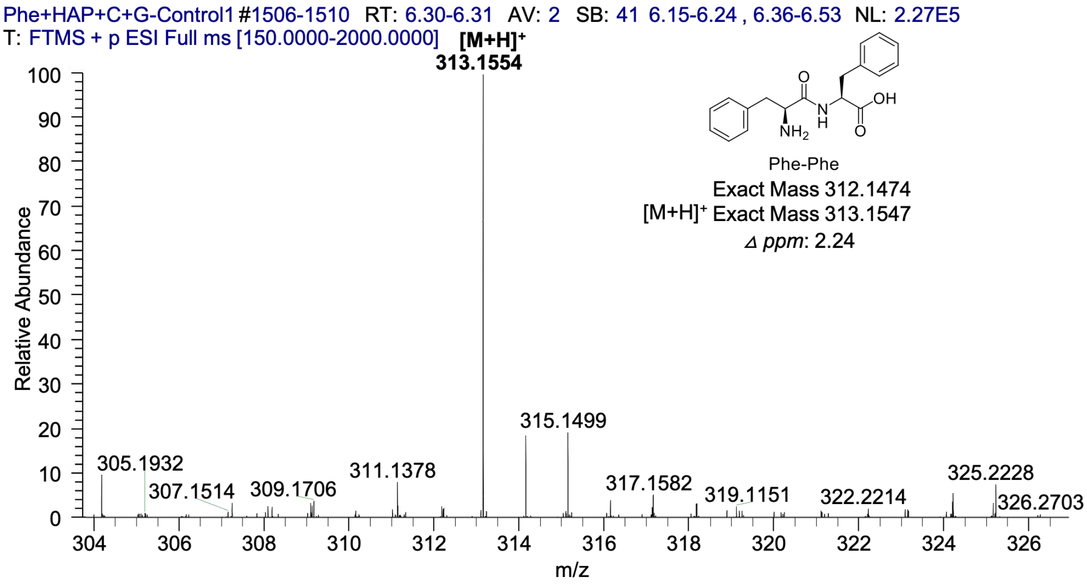

Supplementary Figure 157. The MS spectrum of the product Phe-Phe (*m/z* 313.1554) in the absence of forsterite.

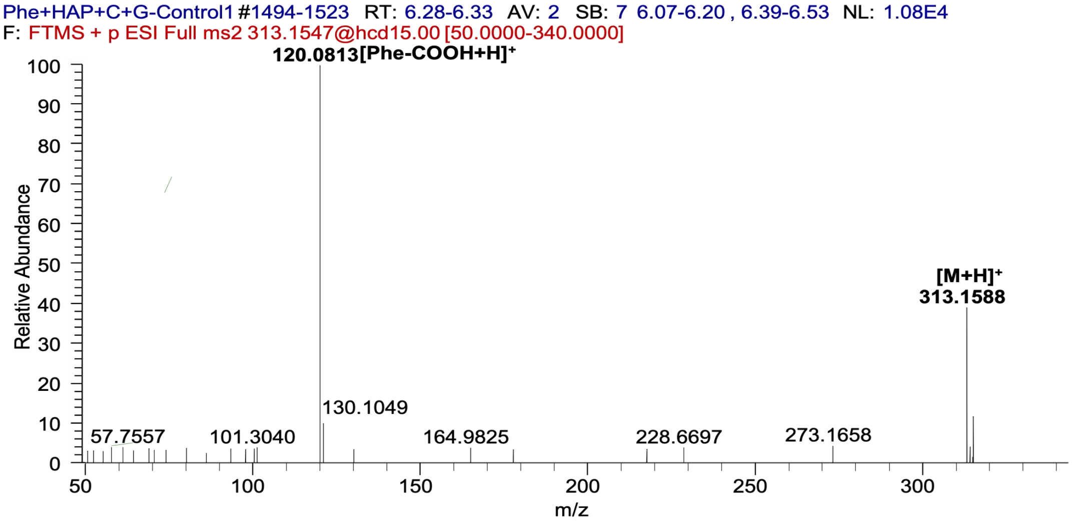

Supplementary Figure 158. The MS^2 spectrum of Phe-Phe (*m/z* 313.1588) in **Supplementary Figure 157**.

20.4 Quantitative analysis of products formed in the Phe and hydroxyapatite system under the ground radiation conditions

All the samples mentioned in sections 20.1-20.3 were quantitatively analyzed by QTrap^{TM} 5500 LC-MS. In addition to the samples with radiation of 63.63 mGy, the samples with radiation doses of 1 Gy and 10 Gy were also analyzed to explore the effect of radiation dose on product generation.

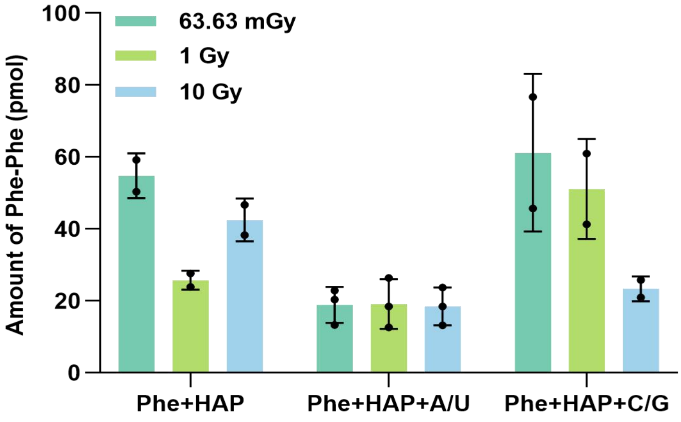

Supplementary Figure 159. The quantitative analysis of product Phe-Phe in the Phe and hydroxyapatite reaction system with forsterite under different radiation conditions. The amount of Phe-Phe formed in the reaction system without forsterite is below the quantification limit.

21. Calibration curves of dipeptide or nucleotide using AB SCIEX QTrap™ 5500 LC–MS instrument

Sample Preparation: Dissolve dipeptide (Ala-Ala, Ala-Phe, Phe-Phe, Phe-Ala and Phe-Arg) in pure water to make mother liquors, which are then diluted to five different concentrations and tested by AB SCIEX QTrap™ 5500 LC–MS. Make calibration curves based on test results.

Chromatography: ACQUITY UPLC I-Class PLUS system, Poroshell 120 HILIC column (particle size: 1.9 μm, dimensions: 2.1×150 mm) at room temperature; flow rate 0.2 mL·min^{-1}. Mobile phases: 20 mmol/L Ammonium formate in water (A), acetonitrile (B). Gradient: 80% B total analysis time 20 minutes. Injection volume 5 μL.

Supplementary Table 4. Mass parameters for the quantitative analysis of dipeptide and NMP by AB SCIEX QTrap^{TM} 5500.

Analytes	Q1 Mass (Da)	Q3 Mass (Da)	Dwell time (msec)	DP (V)	CE (V)
Phe-Phe	313.1	120.1*	50	48	28
		166.1	50	48	19.5
Ala-Ala	161.0	90.0*	50	10	13
		115.0	50	10	12
Phe-Ala	237.1	120.1*	50	20	20
		130.1	50	20	50
Phe-Arg	322.1	175.1*	50	70	29
		120.1	50	70	37
Ala-Phe	237.1	166.1*	50	25	17
		120.1	50	25	28
AMP	348.1	136.0*	20	70	24
UMP	323.0	79.0*	20	-80	-52
		210.9	20	-80	-23
CMP	322.0	79.0*	20	-110	-25
		211.0	20	-110	-23
GMP	362.0	79.0*	20	-100	-27
		211.0	20	-100	-25

Notes: * means the fragment ions used for quantitative analysis and the rest for qualitative analysis. DP, declustering potential; CE, collision energy.

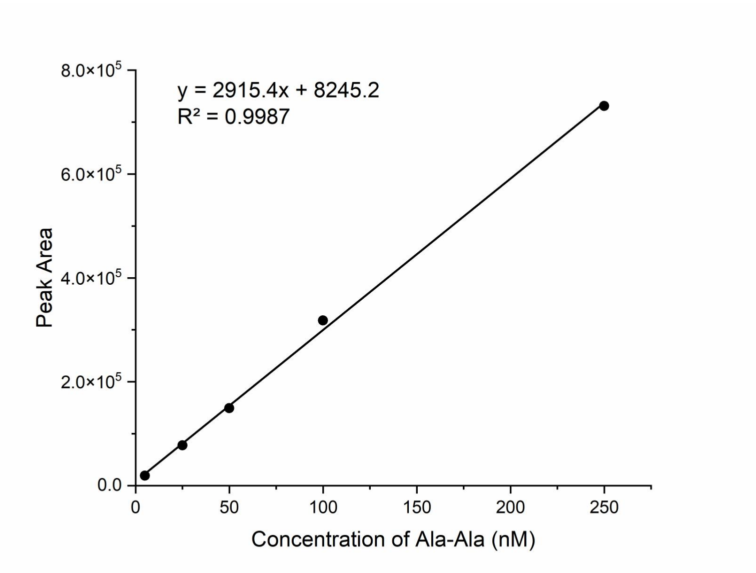

Supplementary Figure 160. Calibration curve of the Ala-Ala.

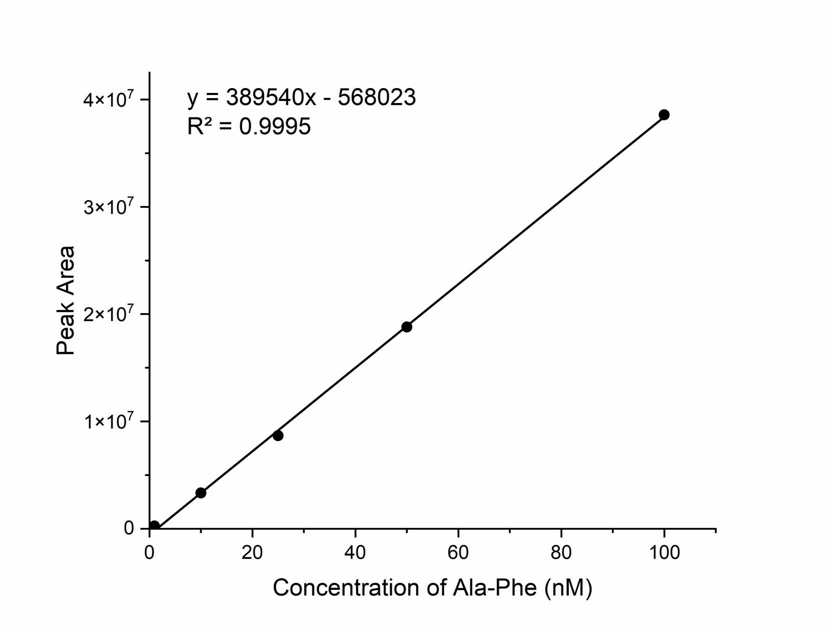

Supplementary Figure 161. Calibration curve of the Ala-Phe.

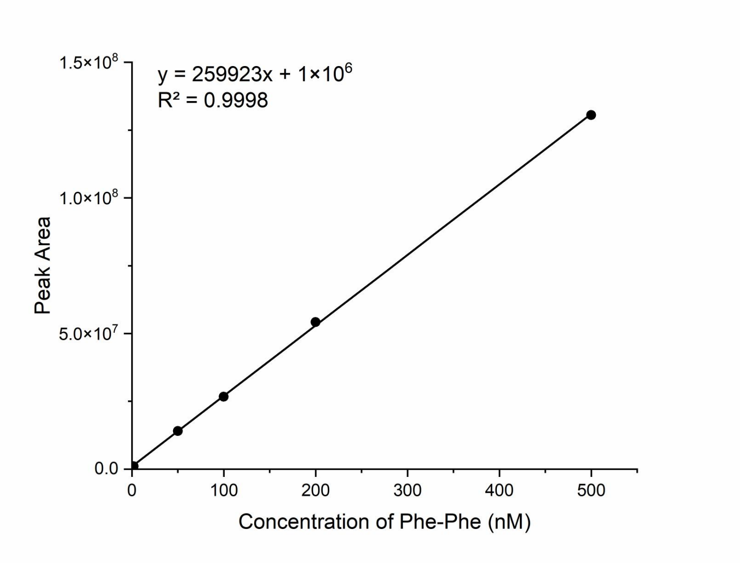

Supplementary Figure 162. Calibration curve of the Phe-Phe.

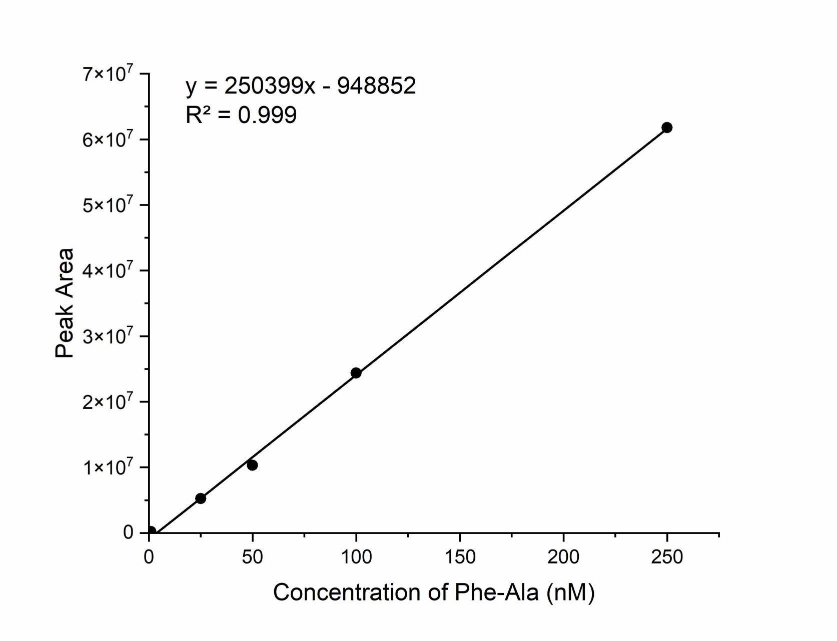

Supplementary Figure 163. Calibration curve of the Phe-Ala.

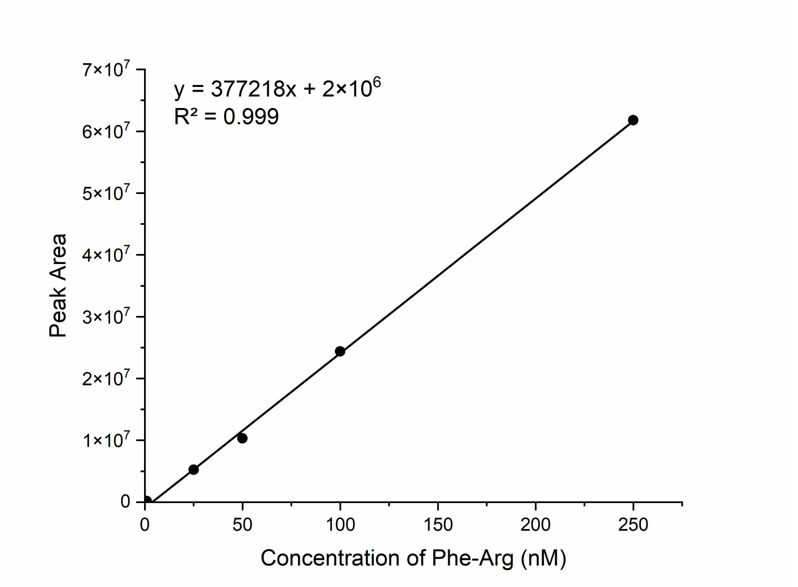

Supplementary Figure 164. Calibration curve of the Phe-Arg.

Sample Preparation:

Dissolve nucleotide (AMP, UMP, CMP and GMP) in pure water to make mother liquors, which are then diluted to five different concentrations and tested by AB SCIEX QTrap™ 5500 LC–MS. Make calibration curves based on test results.

Chromatography:

ACQUITY UPLC I-Class PLUS system, ACE Excel 3 AQ, 3 μm, 2.1 mm × 150 mm column at room temperature; flow rate 0.2 mL·min^{-1}. Mobile phases: 0.1% formic acid in water (A), 0.1% formic acid in acetonitrile (B). Gradient: 0-2 min, 0% B; 2-12 min, 0-100% B; 12-13 min, 100-0% B; 13-20 min, 0% B and total analysis time 20 minutes. Injection volume 5 μL.

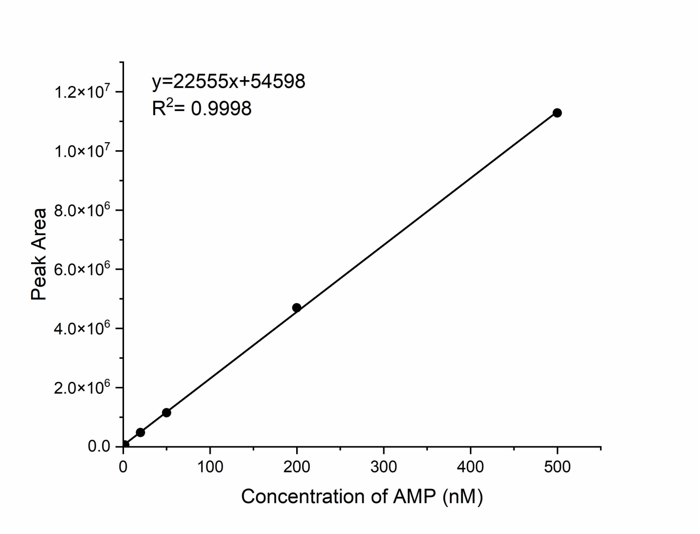

Supplementary Figure 165. Calibration curve of the AMP.

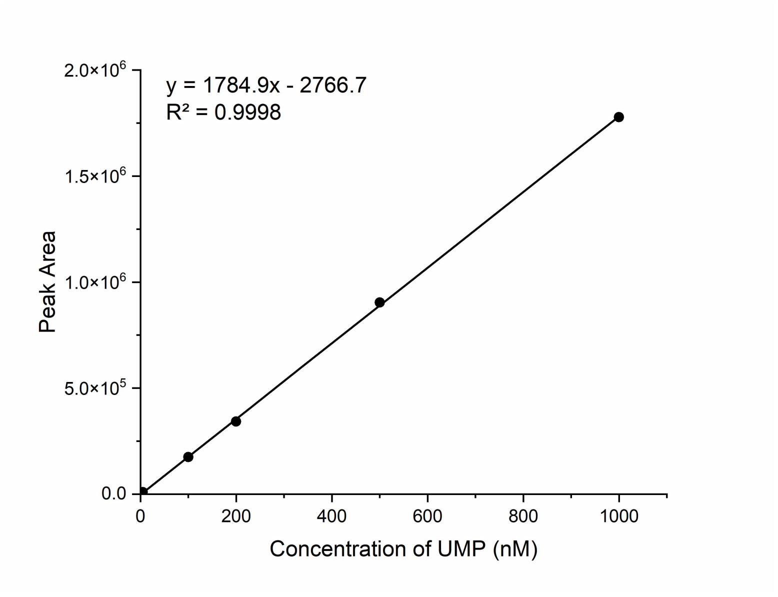

Supplementary Figure 166. Calibration curve of the UMP.

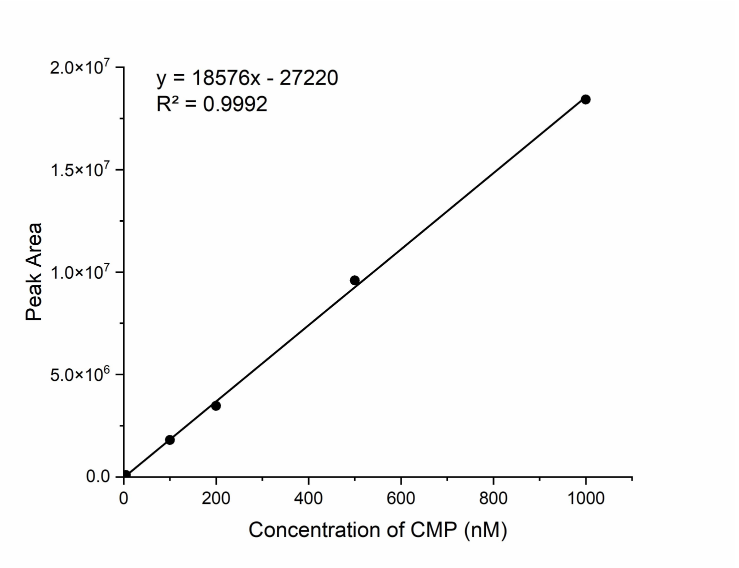

Supplementary Figure 167. Calibration curve of the CMP.

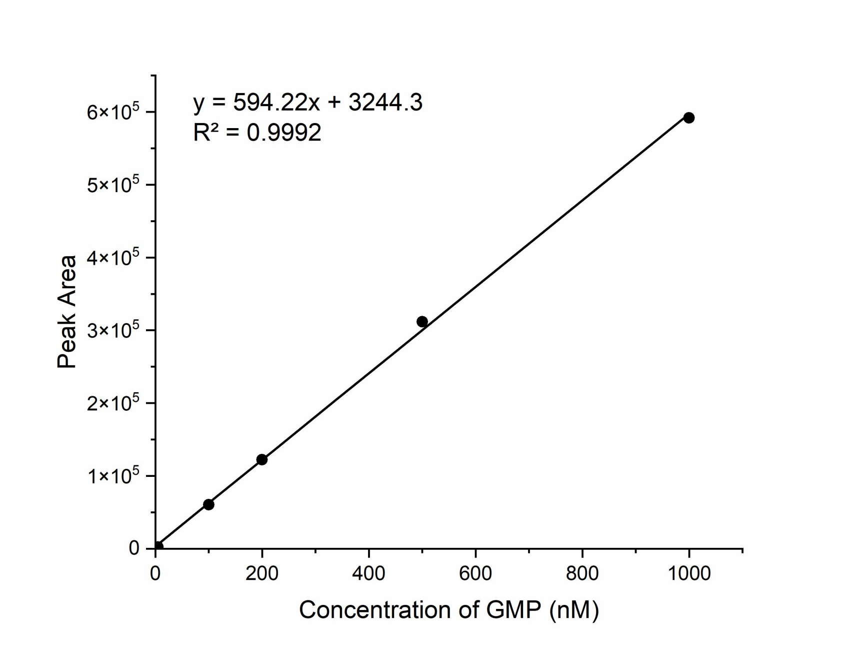

Supplementary Figure 168. Calibration curve of the GMP.

For the quantification of each sample batch, a new standard curve shall be established, as illustrated in the above examples.

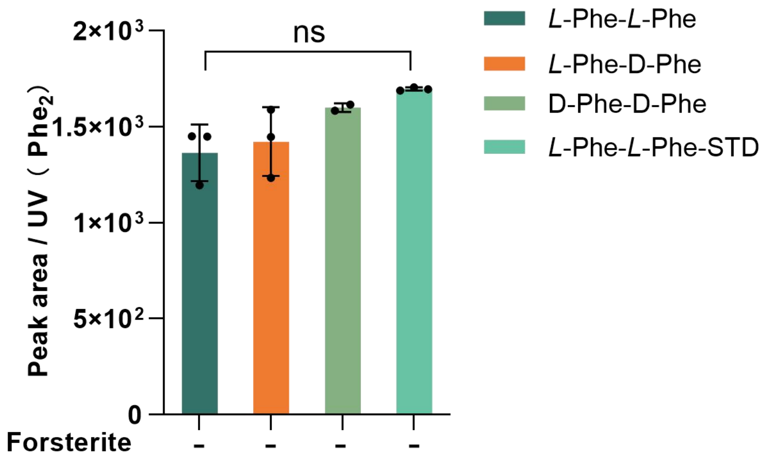

Supplementary Figure 169. The quantitative analysis of Phe-Phe without the aid of forsterite under the CSS radiation with a total dose of 37.75 mGy in C3 Unit. Statistical analysis was conducted using GraphPad Prism version 8.0. ns: not significant.

22. Radiolytic yields (*G* value)

The *G*-value quantifies the radiation chemical yield, representing the number of molecular entities formed or destroyed per 100 eV of energy deposited in an irradiated system (Baird JK, *et al. J. Appl. Phys.* 1990, **68**, 3661-3668). *G*-value can be calculated according to the equation (1).

$$\boldsymbol{G = N/E_{abs}(eV)\times 100} \qquad (1)$$

Notes: N=n×N_A ; E_{abs}=D×m. D (Gray, Gy), 1 Gy= 1 J/Kg. 1 Gy=6.242 ×10^{18} eV/Kg

N: The amount of molecules; n: total molar amount of the target molecule produced (or consumed) in the irradiated sample; N_A: Avogadro constant, 6.02214076 × 10^{23} mol^{-1}; m: Total mass of the irradiated sample; D: Average absorbed dose of the sample; E_{abs} (eV): Total radiant energy absorbed.

Taking the Phe-Arg produced under forsterite-present conditions in sample TZ 6 (Table 1) as an example, irradiation with a total dose of 37.75 mGy resulted in a yield of 29.39 pmol. The total mass of the radiated samples was approximately 50 mg, with a corresponding *G*-value of 150 molecules per 100 eV.

G=[N/E_{abs}(eV)]×100

=[29.39×10^{-12}×6.02×10^{23}/37.75×10^{-3}×6.242×10^{18} eV/Kg×50×10^{-3}×10^{-3} Kg]×100

$=[29.39\times6.02\times10^{11}/37.75\times6.242\times50\times10^{9}]\times100$

$=150$

The relatively high *G*-value calculation may be attributed to the reported dose of 37.75 mGy in this reaction system on the CSS (Supplementary Figure 1c**,** lithium 6 or 7 passive detection chips**)** mainly corresponds to low-LET radiation (<10 keV/μm), comprising protons, electrons, thermal neutrons, and X/γ rays without accounting for high-LET heavy ions. Radiolytic yields with heavy ions are complex, and they depend on both LET and nuclear charge (LaVerne JA, *et al. Radiat. Phys. Chem.,* 2002, **60**, 253-257). The space radiation environment is extremely complex. Since the direction of the LET in space is random and there is no definite incident direction, current detectors cannot measure in all directions. Instead, they can only provide measurements for certain directions, and these measurements are incomplete.

Given that the *G* - value here does not represent a completely accurate situation, we just took one product as an example to make a preliminary estimate of the *G* - value in our reaction system and did not provide the *G* - value for each product.
